# CeleST: Computer Vision Software for Quantitative Analysis of *C. elegans* Swim Behavior Reveals Novel Features of Locomotion

**DOI:** 10.1371/journal.pcbi.1003702

**Published:** 2014-07-17

**Authors:** Christophe Restif, Carolina Ibáñez-Ventoso, Mehul M. Vora, Suzhen Guo, Dimitris Metaxas, Monica Driscoll

**Affiliations:** 1 Center for Computational Biomedicine Imaging and Modeling, Rutgers, The State University of New Jersey, Piscataway, New Jersey, United States of America; 2 Department of Molecular Biology and Biochemistry, Nelson Biological Laboratories, Rutgers, The State University of New Jersey, Piscataway, New Jersey, United States of America; UCSD, United States of America

## Abstract

In the effort to define genes and specific neuronal circuits that control behavior and plasticity, the capacity for high-precision automated analysis of behavior is essential. We report on comprehensive computer vision software for analysis of swimming locomotion of *C. elegans*, a simple animal model initially developed to facilitate elaboration of genetic influences on behavior. *C. elegans* swim test software CeleST tracks swimming of multiple animals, measures 10 novel parameters of swim behavior that can fully report dynamic changes in posture and speed, and generates data in several analysis formats, complete with statistics. Our measures of swim locomotion utilize a deformable model approach and a novel mathematical analysis of curvature maps that enable even irregular patterns and dynamic changes to be scored without need for thresholding or dropping outlier swimmers from study. Operation of CeleST is mostly automated and only requires minimal investigator interventions, such as the selection of videotaped swim trials and choice of data output format. Data can be analyzed from the level of the single animal to populations of thousands. We document how the CeleST program reveals unexpected preferences for specific swim “gaits” in wild-type *C. elegans*, uncovers previously unknown mutant phenotypes, efficiently tracks changes in aging populations, and distinguishes “graceful” from poor aging. The sensitivity, dynamic range, and comprehensive nature of CeleST measures elevate swim locomotion analysis to a new level of ease, economy, and detail that enables behavioral plasticity resulting from genetic, cellular, or experience manipulation to be analyzed in ways not previously possible.

**This is a *PLOS Computational Biology* Software Article**

## Introduction

Understanding how neuronal circuits are assembled, function, and change in response to environmental cues is a major challenge in current neuroscience. Much insight into these issues has been gleaned from studies in model systems that feature relatively simple nervous systems amenable to experimental manipulation [1]. For example, in the nematode *Caenorhabditis elegans*, researchers can place specific gene activities or individual neurons into behavioral circuits to explain the molecular, cellular, and physiological bases of locomotory control, proprioception, and environmental influence [2], [3].

Deciphering genetic and epigenetic specification of functional circuits relies on high precision behavioral analysis. In *C. elegans*, powerful programs for tracking and measuring crawling on a solid surface have markedly enhanced the resolution and throughput with which linear trajectories and turns can be analyzed and by which subtleties in mutant behaviors can be quantitated [4]–[11]. Less extensive analysis has been accomplished for locomotion in liquid, a behavior likely also important in natural environments. Swimming involves more degrees of freedom of body movement than crawling, and might utilize circuitry distinct from crawling [12]–[19]. In addition, swimming behavior can change to alternation between swim and quiescent states after long efforts (2 hr) [12], and swim vigor diminishes markedly as animals age, in part due to sarcopenic deterioration of muscle [20], [21]. Thus, a wealth of fundamentally interesting aspects of swimming behavior could benefit from quantitative high-throughput analysis in *C. elegans*.

Computational analyses of swim behavior that are both comprehensive and automated are missing from the toolbox of behavioral analyses for *C. elegans*. The pioneering work on *C. elegans* swim analysis, although greatly informative [14], [15], [22]–[25], has not yet reached a level of automation and mathematical precision required for full exploitation of the model. For example, published analyses have mostly used tracking programs to generate data on individual swimmers that are interpreted using measurements that involve manual examination. Scoring often involves evaluation of only part of the body movement (such as a head bends) and measures focus on evaluation of regular patterns because arithmetic approaches used are unable to represent dynamic irregularities in swim patterns such as reversals and changes in intensity of effort. The consequence of limited swim locomotion analysis is that important aspects of swim behavior are missed or not scored, and high-throughput applications have not been implemented.

To address the need for comprehensive, fully automated, and biology-driven analyses of *C. elegans* swim motion, we developed the program CeleST (*C. elegans* Swim Test), which accomplishes multi-animal tracking, measurement, and data analysis without need for investigator intervention. Key to the measurement parameters we define are the mathematically-based analyses of individual curvature maps of swimming nematodes over time. Evaluating swim posture using this approach enables calculation of instantaneous measures that extensively describe behavior, with applications for both single animals and large sample sets. Here we document examples of CeleST utility by identifying previously unknown features of age-related wild-type and mutant swim behavior. We envision that the CeleST behavioral analysis package (Dataset S1) will be an invaluable tool for evaluation of mutant phenotypes, dissection of neuronal circuitry, probing of behavior and plasticity, and screening for chemical/genetic activities ranging from anti-helminthic to anti-aging effects.

## Design and Implementation

**Figure 1:**
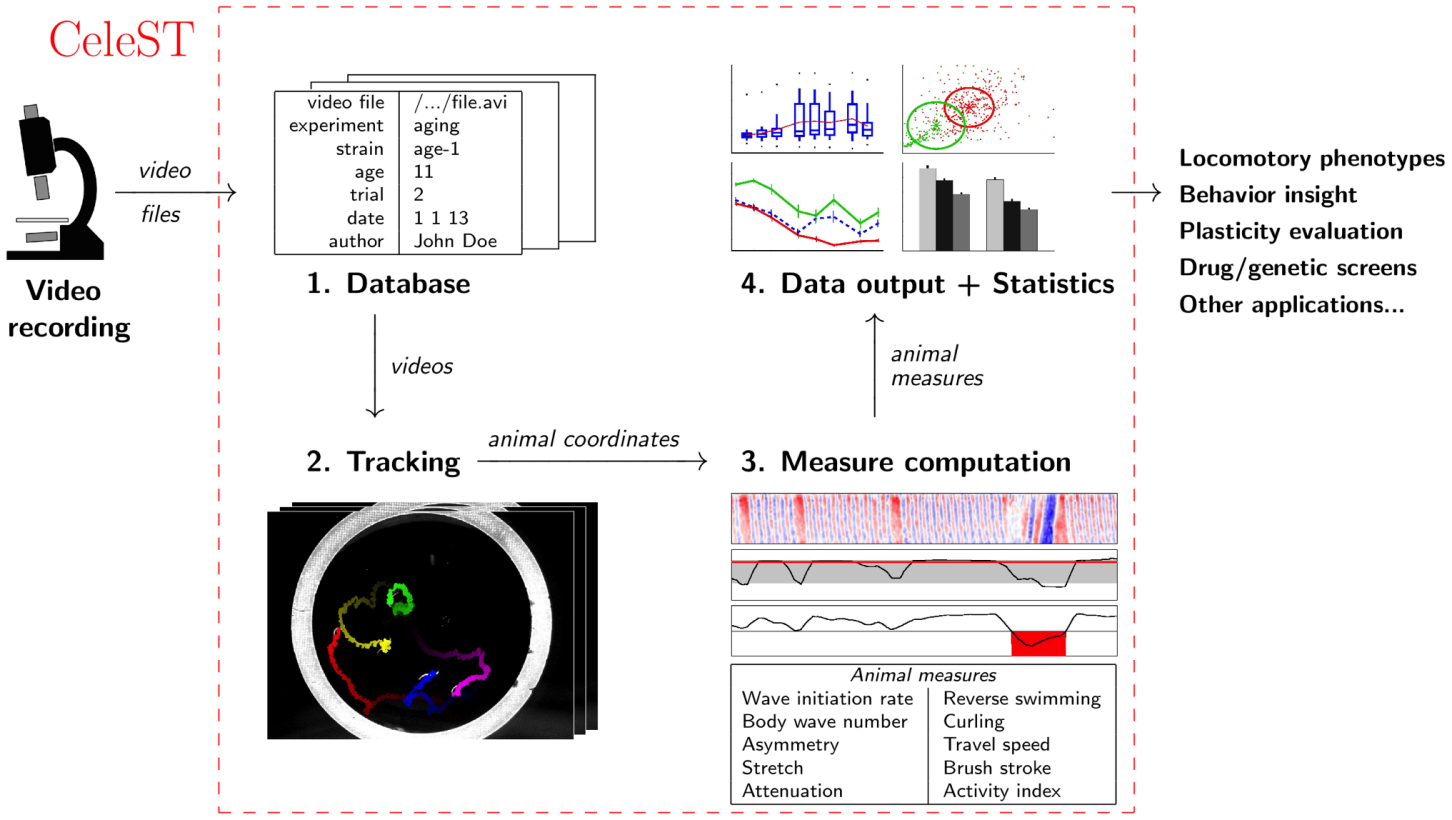
Summary of CeleST components and usage. Input files are videos of multiple swimming *C. elegans*. Files are stored in a database that records identifying features (strain, date, etc.) to permit easy selection of animals to be compared by analysis. After selection of animals to be compared, swimmers are automatically tracked from videos, and computation from curvature data or posture is used to score ten swim measures in 30 second swim trials (see description in text). Measures from the scored animals are compiled and can be exported in several alternative data analysis formats, including dot plots, line graphs, histograms and two dimensional comparison (ellipses indicate the principal directions and the standard deviations of the data). Statistical analysis is automated. A dynamic demonstration of CeleST tracking and computing of measures can be found in Video S1 1–4 on http://celest.mbb.rutgers.edu.

Software tool CeleST provides an automated, quantitative, and detailed description of *C. elegans* swimming (Figure 1). The full package includes: 1) a database, 2) a multi-animal tracking algorithm, 3) a set of ten automated measures of swim features, and 4) a plotting tool through which the user can group and compare all the videos in the database, graphing measures as 1D charts, 2D plots, or histograms (all with statistical treatment). We designed the interface of the software to be a user-friendly platform that facilitates intuitive access to the different CeleST components. Overall, this automated analysis package constitutes a major advance in the efficiency and resolution with which swimming behavior can be analyzed.

### Database

We recorded the locomotion of animals in liquid and created a repository of videos for analysis. The location of each video can then be imported into the CeleST database, which further differentiates the video file with the following tags: sample number (unique identifier), date recorded, investigator, experiment, trial number within the experiment, strain, animal age, number of animals, and time length of the video. We found that these tags were sufficient to identify videos to perform multiple comparisons for a range of purposes, but we note that additional tags can be added to the database at any point to customize to application.

### Tracking

Our videos typically contained four to five adults in the same swimming zone to minimize overlapping of animals, which is a challenge for effective automated tracking. Briefly, the first step of the tracking (called segmentation in computer vision) required locating the animals in a single image. This was achieved by filtering the image with a 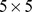 pixel (the typical width of an animal in our videos) standard deviation filter, which enhanced the edges of the animals and reduced much of the visual noise affecting our videos. We then computed a gradient on the filtered output delineating the edges of the animals. We used a greedy line-growing method to find the closed contour that maximized the gradient flow, and the resulting lines gave us the outlines of the animals. We then measured the inner distance transform of these outlines: the inner ridge of the transforms were the center body lines of the animal (lines of lateral symmetry), denoted by 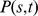, where 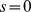 is at the tail and 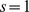 at the head, and 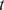 is the time stamp of the image; the value of the distance transform at 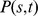 is the half-width of the animal's body at this location, denoted by 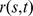. Note that the head-tail differentiation was actually computed later on; if an animal appeared to be reverse swimming more than 50% of the time, its head and tail were switched and all measures were updated. For tracking purposes, the head-tail differentiation had no effect.

Once we segmented an image, we tracked the resulting animals in successive frames. Knowing the location of an animal at time 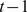 made the location of the same animal at time 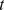 faster. Swimming motions are mostly lateral from one frame to the other, and the frame rate we used (18 images per second) ensured that there was some overlap in the bodies of the animals from 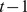 to 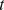. In particular, we found that it was trivial to identify at least two stationary points by comparing the intensities of pairs of consecutive images. We adjusted the rest of the body by a two-step greedy fitting of 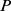 and 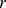 to the new image. Although the tracking was fast, there was always a risk of losing track of parts of an animal. To ensure we did not lose parts of an animal for long, we performed a new segmentation every 20 frames and merged the tracking and segmentation results of these key frames.

We handled contact among animals and self-overlap during the fitting of the points of the tracking step. From the stationary points, we computed the extreme envelope of possible body locations and compared it against all animals. If two animals had envelopes with overlap, there was potential for cross contact of their bodies or self-overlap. For each animal, we first fitted the body parts that were not in the overlapped envelopes. Each new fitting gave us an improved and smaller envelope of possible body locations. If the envelopes still remained in contact after fitting the non-contact points, we fitted the remaining points as normal but the two animals were flagged as being in contact on these frames. We automatically rejected these frames from the computation of measures. However, we enabled the possibility of a human user to check them after tracking to manually validate them if desired.

We then integrated an automatic quality checker step to perform simple tests on tracking results. For instance, if the body length of an animal dropped two standard deviations below the mean body length of the same animal over the entire video (which may happen if an animal gets out of the filming zone), the quality checker flagged this as inappropriate for measures. We chose to automatically reject any animal with over 20% of its video frames flagged as inappropriate, however we saw the value of letting the human user to have the final say on the decision. The recorded swimming of individual animals can be played back visualizing the animal's outline, which is color coded for easy identification of flagged frames. The user can thus review the tracking if desired and override automated decisions by switching the validity of specific frames and/or of the full tracking of an animal.

We performed an evaluation of our tracking method by manually evaluating the tracking results of 2,020 animals from 404 videos. Abiding by our threshold of a maximum of 20% of flagged frames per recorded time, our method only discarded 5.9% out of the 2,020 animals examined and successfully tracked 94.1%. The latter animals had valid outlines within 1-pixel accuracy for 92.1% of the recorded time. With our flag approach, we prevent tracking errors from interfering with the accuracy of the measures. Moreover, we emphasize that our tracking method and our measures are separated. As a result, any existing tracking method that provides body coordinates and width can be used as an input for our measures. Similarly, future measures can be computed on existing results obtained with our tracking method without the need of re-tracking. This capacity for modular applications increases the number of options for use of our software components, making CeleST a particularly valuable toolbox for biologists and software developers. Additional details on the tracking method CeleST uses can be found in refs [26], [27].

### Measures

For each animal, the tracking results provided the coordinates of the central body line as an open polygon at each time frame t. To simplify the notations here, we used the continuous curvilinear coordinate 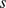 to locate each body point from tail (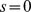) to head (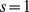). In the practical implementation, we discretized 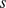 into 12 equally long segments. Each point 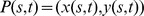 on the central body line is associated with the radius 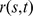 of the body at that location. The curvature 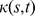 of the body at location 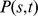 is defined as the derivative of the tangent vector with respect to the curvilinear coordinate, and is computed in Cartesian coordinates as:
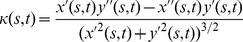
The short-time two-dimensional Fourier transform 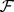 of the curvature over a time interval 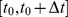 is defined as:
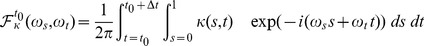
where 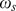 is the spatial frequency and 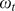, the temporal frequency of the curvature. Since the curvature 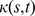 is non-complex, the short-time Fourier transform is symmetric with respect to the origin, and computing it on half of the plane of frequency values is enough for the subsequent analysis. We chose to restrict to positive 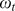. The duration of the time interval 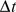 increases the resolution of the time frequency 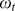 computed by the short-time Fourier transform, but it reduces the amplitude of the mode in 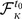 in case the nematode changes its behavior during that time interval. As a tradeoff, we calculated the short-time Fourier transform at every time 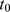, with multiple time intervals 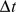, specifically 32, 40, 48, 56 and 64 frames. We then re-sampled these five short-time Fourier transforms so that all had the same time resolution, and averaged them. We note that swimming motions have a single wave component (as opposed to a linear combination of waves), but tend to alter their behaviors over short periods of time. Therefore, a standard static Fourier analysis would not be suitable for the analysis of curvature maps since it would not cope with the swimming motion changes over time.

Based upon computer vision analysis of over 6,000 swimming nematodes, we developed ten distinct measures that score specific features of swim behavior. Together, the ten output measures provide a thorough report on swimming that markedly exceeds analysis capacity of an unaided human observer.

**Figure 2:**
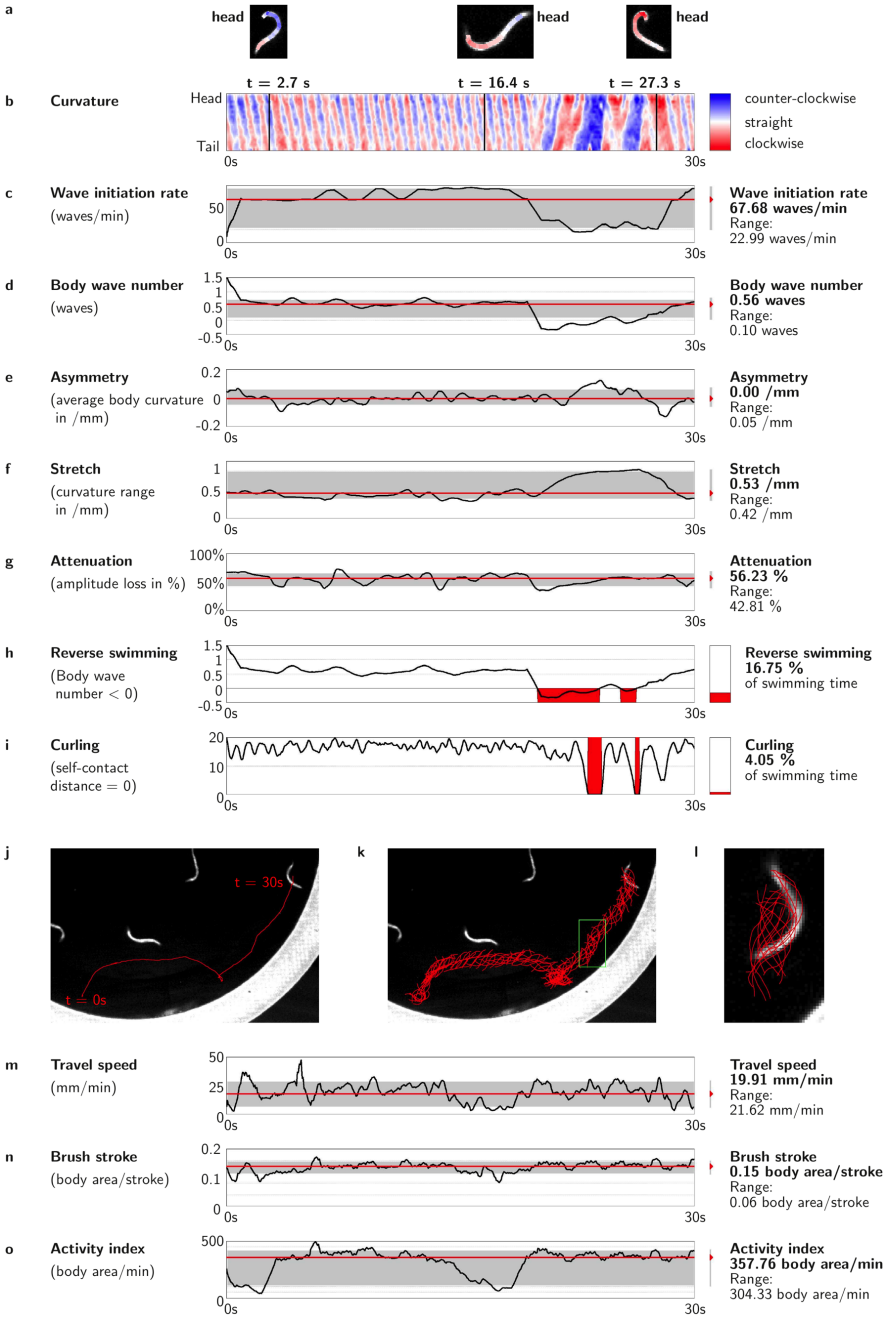
Examples of ten CeleST measure outputs for an individual *C. elegans* swim trial. All measures reflect analysis of a 30/sec, with **C–G** calculated from analysis of radius of curvature over 12 body segments (curvature plot vs. time example is in **B**). For **C–G** and **M–O**, instantaneous values are plotted in black; the median value for each swim is drawn in red, and the 10–90 percentile range of values is shown in gray; median and range over the 30 s trial are listed on the right. Note that although this panel demonstrates analysis of measurements of a single animal swimming, the CeleST program score multiple animals simultaneously and can compile data from thousands of individual swim trials (examples in Figure 3). **A**, Scored animal at three indicated time points in the video, with the curvature measure superimposed on the body; color key shown in **B**. **B**, Curvature heat map of an individual swim trial. Map is of curvature at a given body point (Y axis) as a function of time (X axis), with head curvature score at the top and tail curvature at the bottom on the Y axis, deep bend in one direction dark blue, and in the other direction dark red. Lines indicate posture of the animal depicted at that time point in panel **A**. Note that the posture of an individual at any point in time could be reconstructed from the measure of curvature over body position. Further details on each parameter measurement **C–O** are given in Text S1. Informative videos (Videos S2 01–10) that feature extreme examples for each measure can be found on http://celest.mbb.rutgers.edu.

We demonstrate the ten-parameter evaluations from an individual swim example in Figure 2 (a dynamic demonstration is provided in Video S1 1–4, and extreme measures for each parameter are provided in Video S2 01–10 on http://celest.mbb.rutgers.edu). Six of the ten scored parameters are based exclusively on curvature measurements along body segments. Curvature scores are derived from the inverse of the radius of the best fit circle for a segment corresponding to 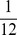 the body length - i.e., small radius is high curvature. 12 body sectors are scored per frame. Note that the curvature calculation relies entirely on local body shape changes within the animal - this self-referential aspect of data collection eliminates the need for threshold determination for those parameters, and facilitates analysis of data derived from any tracking program. In other words, although we recommend use of our powerful multi-tracker, should an investigator prefer to use another tracker, the data could still readily be subjected to the measures that we define, which is a major strength of the CeleST program. We also point out that our metrics are normalized to animal size, so that image resolution, frame rates, or size/shape of the animal do not impact measurement scores.

**Figure 3:**
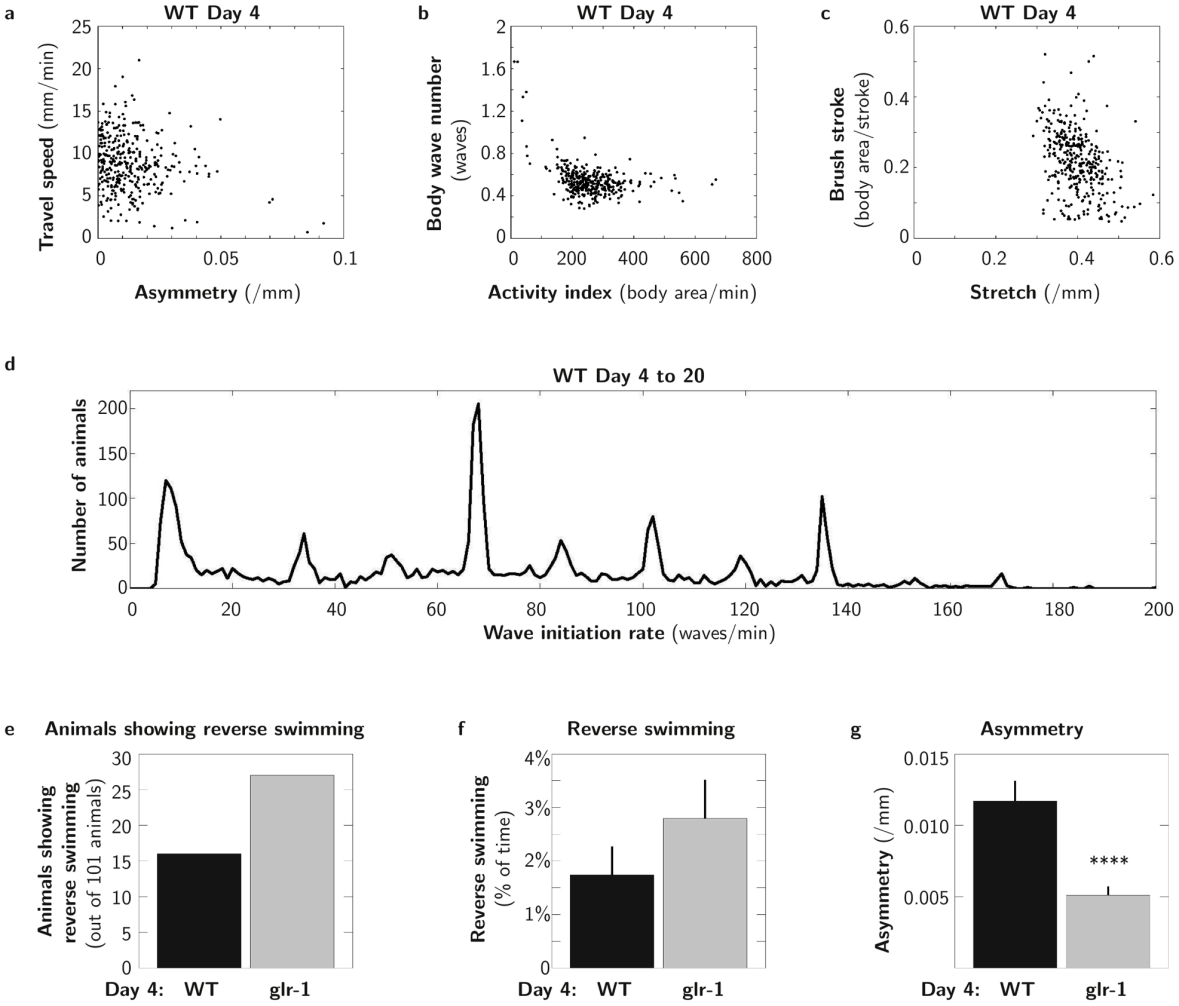
CeleST analysis reveals features of *C. elegans* swimming 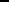 considerable individuality, gait preference, and reverse swimming. **A–C**, *C. elegans* exhibit diverse swimming abilities, despite genetic and environmental homogeneity. CeleST can plot scores for two parameters against each other, for example: **A**, Travel speed vs. Asymmetry; **B**, Body wave number vs. Activity index; **C**, Brush stroke vs. Stretch. Data for 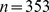 WT 4-day old animals from 9 independent trials are plotted. **D**, *C. elegans* swim at specific wave initiation rates. We plotted in the form of line histograms the distribution of median Wave initiation rates (WIR) in wild-type animals as occurs over a 30 second interval. WIR values are binned to integers and the plot line delineates the contour of the bins in the histogram. X axis is median WIR, Y axis is the number of individuals exhibiting the indicated WIR. Data in this panel are combined to represent 3,372 animals ranging from 4 to 20 days old from 9 independent trials to emphasize the peaked distribution. Although animals in this large population do swim over the range of possible median WIRs (see Figure S1F for example), CeleST analysis reveals an unexpected bias for particular “gaits” in a subset of the population (about 14% total appear in favored WIRs). Older animals swim at lower median WIRs than young adult animals, but the preferred WIRs remain. WIR distributions for specific individual ages are depicted in Figure S1. Note that mean WIR rates do not exhibit a distribution bias (Figure S1F), so this study emphasizes the value of also considering median scores in swim behavioral analysis. **E–G**, For brief periods, swimming animals reverse, with the tail initiating the body wave. Reverse swimming is illustrated in Videos S3 and S4 on http://celest.mbb.rutgers.edu. In 4-day old animals, the *glr-1(ky176)* mutant, lacking a neuronal glutamate receptor, reversal frequency is increased relative to WT (**E**), although the trend to increased time spent in reverse is not statistically significant (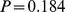) (**F**). Unexpectedly, *glr-1* mutants swim more symmetrically than WT at day 4 (**G**). 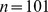 from 3 independent trials for each strain. Data for all 10 measures, young and old age are shown in Figure S2. Error bars show SEM, **** 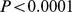.

We plotted local curvature scores over time for one swim trial example in heat map format (Figure 2B). Figure 2C–I and 2M–O graph the individual measure score vs. time in black, with median value for the 30 second trial in red. (Note that median scores are less subject to outlier impact than mean scores, and better reflect the dynamic aspect of a swim trial.) The ten different measures that add up to a comprehensive and detailed analysis of *C. elegans* swimming are as follows:

1. **Wave initiation rate** (Figure 2C) is the number of body waves initiated from either the head or tail per minute (the latter is a rare occurrence, see Figures 2H, 3E). This parameter is akin to the rate of body bends/time that scorers manually record (see Table S1 for comparison of human vs. computer scores), and can be thought of as analogous to the stroke rate of a human swimmer. We calculate wave initiation rate from the time coordinate in the short-time Fourier analysis of the curvature heat map plots. Because of the undulatory nature of the nematode swimming motions, the norm of the short-time Fourier transform 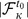 is a single-mode two-dimensional real function. The mode is located at coordinates noted 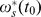 and 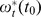. The Wave initiation rate at frame 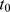 is defined as 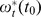. The inverse of that frequency gives the duration of a stroke 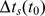 at that time, which is used for several of the remaining measures. In essence, our approach scores the number of stripe occurrences in the heat map/time. Strokes propagating from head to tail (left slant) and tail to head (right slant) have opposite sign in the short-time Fourier analysis. The overall score we use for the wave initiation rate measure ignores the sign, and thus indicates all strokes.
2. **Body wave number** (Figure 2D) measures the number of waves in transit through the body at a point in time, and hence provides a snapshot of the “waviness” of the body posture. We score body wave number as the spatial coordinate of the mode in short-time Fourier analysis, counting how many wave repeat cycles are present in the body at a given moment. A high score (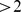) would reflect several body bends per animal; a low score (between 0 and 1) would indicate that a single wave is traveling down the body or that the wave initiated by an extremity reaches the other extremity before the next wave is initiated. A negative score indicates a reverse direction wave. Body wave number is the absolute value of 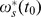 defined above.
3. **Asymmetry** (Figure 2E) evaluates how balanced the swim posture is per stroke, as measured in the focal plane perpendicular to the camera. In effect, wave asymmetry reports on whether the animal bends more toward one side or the other. We score asymmetry by computing the average curvature per stroke, which we derive from the average curvature over the body during the interval covering two strokes at the current stroke duration 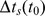 defined above:
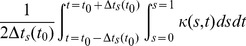
An animal bent clockwise from tail to head will have a positive score, and the stronger the preference for one side, the higher the absolute value of the score. A perfectly symmetrical animal, which would bend exactly as much clockwise and counter-clockwise, would have a score of 0. Note that for the group score, we use the absolute value of the asymmetry to avoid canceling out scores for animals that randomly favor the left or right in relation to the camera.
4. **Stretch** (Figure 2F) measures the maximum differences in curvature that occur between the two most extreme curvature scores at any part of an animal during a given stroke, providing a sense of whether body bends are deep or flat and how much “stretching” effort occurs in a stroke. Thus, we identify the point of largest range in curvature and plot the range over time. We calculate the largest range in curvature at any given time (stretch) from the maximum range of curvature values from any one body part over the course of two strokes:
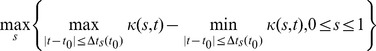

5. **Attenuation** (Figure 2G) measures how well the depth of a wave is maintained as it propagates down the body. We compare the range that the tail covers to the range that the head covered when that wave was initiated. The measure compares head-to-head deformation to the corresponding tail-to-tail deformation as movement occurs over time. An attenuation value of 0% has equal head and tail amplitudes (no attenuation), a value of 100% corresponds to an animal in which the head was active but the tail did not move. This measure gives an idea of how coordinated movement is from head to tail, and might be particularly valuable for locomotory mutant analysis. We have noticed that negative attenuation, or amplification, can be observed during episodes of reverse swimming. Mathematically, attenuation is the residual ratio of the maximum range of curve of the tail (defined as the lower quarter of the body) to that of the head (defined as the upper quarter) over the course of two strokes (equation below), which is later converted to percentage.
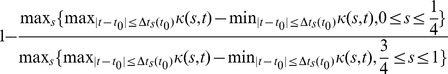

6. **Reverse swimming** (Figure 2H) measures the percentage of time that an animal swims in reverse, initiating a body wave from the tail that propagates toward the head (Videos S3 and S4 on http://celest.mbb.rutgers.edu). On the curvature vs. time heat map, a reversal is evident as a switch in direction from a leftward slant to a rightward slant. This measure detects the precise time frames when reversing occurs in contrast to a human observer (Table S1), and summarizes them as a percentage of swimming time. Reverse swimming is detected as a disagreement in sign between 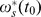 and 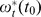. Since we restricted the computations of the Fourier transform to positive 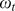, it is detected as a negative 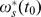 (below). The resulting value is later converted to percentage.
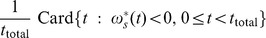

7. **Curling** (Figure 2I) measures the relative percentage of time that an animal spends bent around so far that it overlaps with itself. Swimming *C. elegans* can sometimes stop and curl up into a shape that nearly resembles the letter “O” or the number “6”. We detect a curl by computing the distance from either extremity to the opposite side of the body; “O”, “6”, and shapes in between are counted. (We define a 6-shape type of curling when the distance between opposing extremities is a third of the animal body length.)
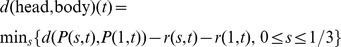


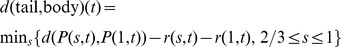
We then calculate curling as follows and present the value as percentage.
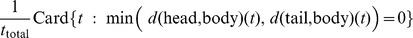
The sensitivity of CeleST in detecting curling is illustrated in Video S2 7of10 on http://celest.mbb.rutgers.edu, and contrasted to that of an aided observer in Table S1.
8. **Travel speed** (Figure 2M) reflects the distance that an animal travels during a defined time. Some animals move over significant distances when they swim, whereas others remain in one place even though they exhibit considerable bending activity. To measure travel speed, we identify the animal's body center over a two-stroke interval and track its trajectory:
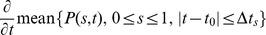
This measurement approach cancels out the small back-and-forth lateral motions that are caused by stroking—lateral movements do not contribute to directional longitudinal traveling score.
9. **Brush stroke** (Figure 2N) reports on the area that the body of the animal would “paint” (the number of pixels covered) in a single complete stroke, giving an indication of the depth of the movement and the extent to which the animal has flexed in a given stroke. Specifically, we derive this measurement from the residual ratio of the number of pixels covered during two strokes to the number of pixels of the animal body (below), with averages of successive pairs of strokes over time represented in the graphic output.
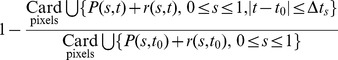
The brush stroke measure is complementary to the curvature-based measures above. Curvature-based measures consider local body deformations independently of their effects on the animal's location; the brush stroke measures the local body locations independently of which body part was actually deforming.
10. **Activity index** (Figure 2O) sums up the number of pixels that are painted by the body during the time that it takes an animal to do two strokes to provide a sense of how vigorously the animal bends while swimming over time. Thus, the Activity index is the Brush stroke normalized by the time taken to perform the two strokes:
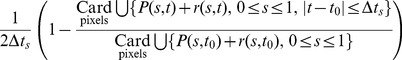
Note that two measures, Reverse swimming and Curling, are different from other measures in that scores correspond to binary categorical data (in reverse or not, curled or not) over time; we summarize these measures as the percentage of time during which an animal displayed either behavior. Moreover, we emphasize that for each measure except Reverse swimming and Curling, the values are computed per frame, producing time series reflecting the swimming behaviors and their changes “as-they-happen”. We summarize these time series using temporal medians because dynamic changes during swim trials impact mean scores in a way to skew mean values away from the most common behavior. The median score provides a more faithful picture of the animals' behaviors.

As is evident from Figure 2, the ten measures are computed continuously over time, for each frame of the video, so that the trace record reflects the behavior of an individual animal “as-it-happens”. We noticed that many animals are not consistent during 30 s for several of the measures. For example, the Wave initiation rate might drop for a few seconds and go back up again (Figure 2C), or the Activity index might oscillate (Figure 2O). We anticipate that the variations detectable in CeleST single animal reports may provide the basis for novel insight into temporal patterns of swimming behaviors. The range of values reached during a swimming trial reflects the consistency of an individual's behavior, which we illustrate with the 10–90 percentile range in light gray in swim trial reports (exemplified in Figure 2). The temporal variation report format also makes possible visual comparison across measures. For example, we have observed cases of simultaneous lower attenuation, lower wave initiation rate, and negative amplitude during reverse swimming. For quantitative population studies, we focus on the median value swim behavior of each animal over a 30 second period.

## Results

### Wild-type exhibits a broad range of dynamic swimming abilities

In the course of CeleST development, we examined single and combined parameter scores for thousands of animal swims (examples in Figure 3A–C). (Animal growth conditions and swim analysis are detailed in Text S2.) Two-dimensional plots that reflect scores of two distinct measures of individual swimmers emphasize that individual *C. elegans* swim with a striking diversity of styles, which was initially unexpected given the genetic homogeneity of *C. elegans* and the uniform environment in which they are raised. Despite these differences in swim skill level or vigor of effort, however, we find that wild-type (WT) animals exhibit a definitive preference for specific median Wave initiation rates (WIR) (Figure 3D). We plotted the median WIR vs. the number of animals that adopted that given WIR to note that rather than swimming over the full range of possible WIRs, animals disproportionately adopt specific WIRs, revealing a previously unknown preference for particular swim “gaits”. At young ages, animals more often select high WIRs, and at older ages, they more often swim at slower WIRs, although distinct WIR preferences, rather than a full range of WIRs, are apparent through most of adult life (Figure S1). Interestingly, the median WIR is the only parameter for which we noted distinct preferences—other parameters exhibit graded scores, and this trend is not apparent when mean WIR is visualized (Figure S1F). The existence of different gaits between crawling and swimming [17], [18], [28], [29], or for different food availability conditions [30], [31] have been previously debated, but the finding of selective swim WIRs is novel.

### *C. elegans* can reverse and briefly swim backwards, albeit infrequently

*C. elegans* have been noted to swim “backwards”, initiating a body wave from the tail that travels toward the head [32], but quantitative scoring of swim reversals has not been reported. Our data analyses firmly establish that WT animals do swim backwards for brief periods (Figures 2H, 3E,F; Videos S3 and S4 on http://celest.mbb.rutgers.edu). In young adult *C. elegans*, 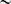
15% of 30s swim trials include a brief reverse (Figure 3E), although on average only 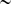
1–2% of swim time is spent in reverse (Figure 3F). Backward swimming may not have been previously analyzed in locomotion analyses because existing programs were not written to accommodate irregularities in swim behavior. We emphasize that CeleST has the capacity to evaluate all possible curvatures and thus can detect any specific locomotion pattern of interest, even if irregular. Thus, the CeleST program could be exploited to select for, or identify, highly specific deficits in swim pattern in genetic or pharmacological screens.

### New phenotypes of a neuronal signaling mutant

Our expectation is that CeleST will uncover subtle phenotypes not readily apparent to the observer. For example, we studied *glr-1(ky176)* (disrupted for a neuronal glutamate receptor subunit), for which modest changes in crawling reversal frequency in response to food [30] and swim turning [32] have been reported. We find that in young adults, *glr-1* mutants swim similarly to WT except for an unexpected increase in how symmetric the swim behavior is (Figure 3G). We also detect an increase in the number of *glr-1* mutants that exhibit a reverse while swimming (Figure 3E), but the overall difference in percent of time spent swimming in reverse is not statistically significant (Figure 3F). Differences in multiple parameters, however, become evident later in life (day 11), suggesting that *glr-1*, defective for neuronal signaling, has accelerated ageing decline (Figure S2). Our data suggest *glr-1* promotes a swim-side preference in young adults, reveal that *glr-1* (and presumably the accompanying glutamate-driven neuronal activity) is important for adult maintenance of behavioral integrity, and support that CeleST can point out differences that may be subtle and transient.

### Details of age-associated behavioral changes in swimming prowess

**Figure 4:**
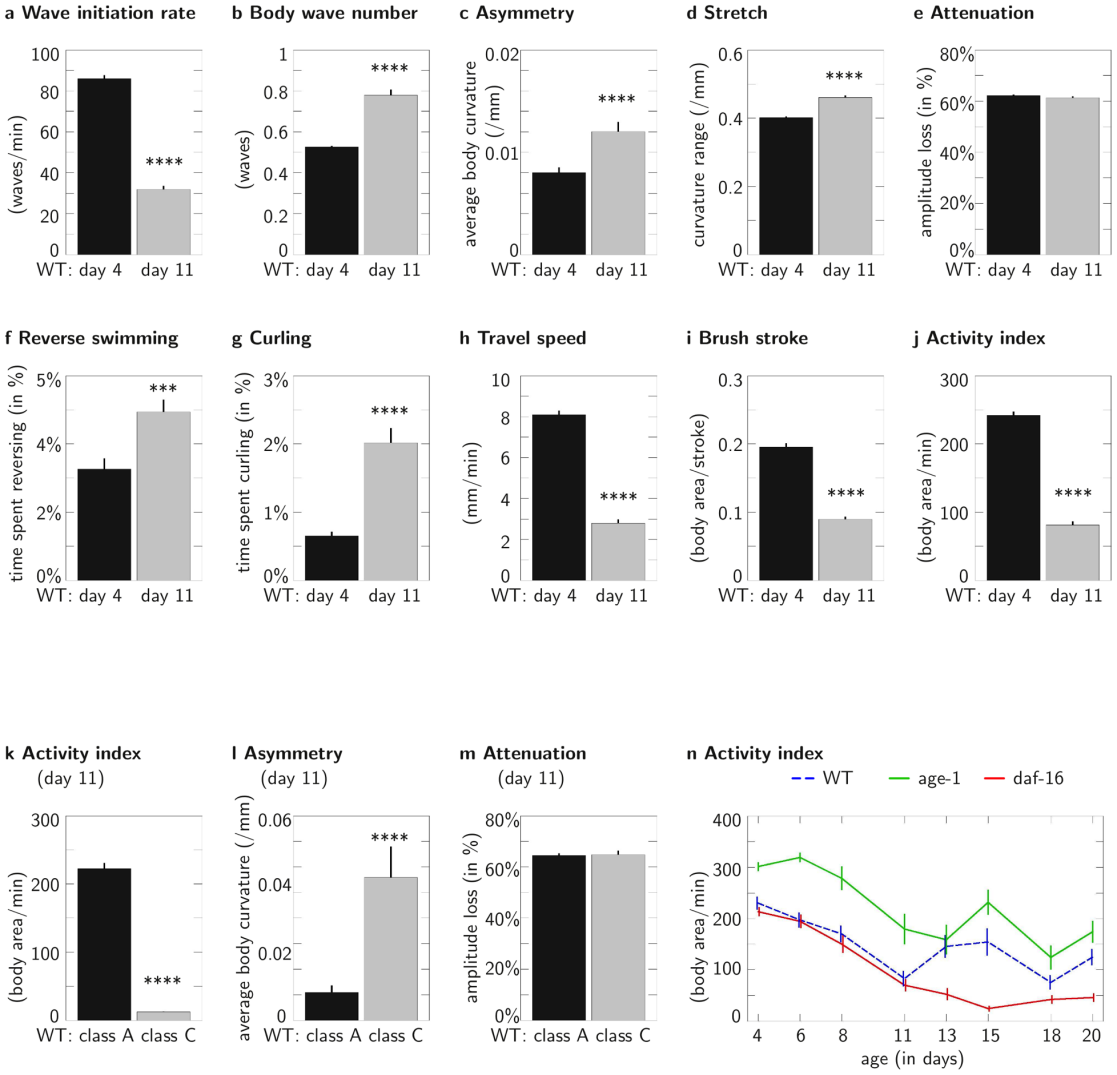
CeleST reveals novel information on aging phenotypes. **A–J**, Age-associated changes in swimming parameters in wild-type adults. **A**, Wave initiation rate; **B**, Body wave number; **C**, Asymmetry; **D**, Stretch; **E**, Attenuation; **F**, Reverse swimming; **G**, Curling; **H**, Travel speed; **I**, Brush stroke; and **J**, Activity index. 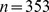 from 9 independent trials, for each age day 4 and day 11. Data for ages ranging from day 4 to day 20 are presented in Figure S4. **K–M**, CeleST reports great differences in graceful agers vs. poor agers for measures that change with age. We selected animals that appeared to have robust crawling capacity (Class A, graceful agers) and those that had decrepit crawling capacity (Class C, poor agers) at day 11 and compared swim behavior. **K**, Activity index; **L**, Asymmetry; **M**, Attenuation. 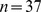 from 3 independent trials, for each class. Data for all ten measures in this series are shown in Figure S5. **N**, Locomotory changes under life-extending and progeric insulin signaling pathway manipulation suggest complex influences of signaling over the lifetime. Activity index, WT: blue line (middle dashed line); long lived *age-1(hx546)*: green (top line); short-lived *daf-16(mgDf50)*: red (bottom line). Note that the *age-1* mutant has a higher activity index in young adult life as compared to WT, which suggests differences in swim performance are not limited to aging. Also, at day 15, WT and *age-1* scores appear increased, which we suggest reflects the preferential death of the poorest swimmers, rather than an actual increase in average swimming of individuals. 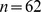 in each data point from 4 independent trials. Data on all measures are presented in Figure S6. Error bars show SEM, *** 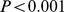, **** 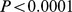.

As in the debilitating human sarcopenia [33], [34], *C. elegans* diminish in locomotory vigor as they age [20], [35], [36], a phenomenon that tracks with physical deterioration of muscle [20], [37] and synapses [38]. To further test how robustly CeleST programs could reveal subtle changes in swimming behavior, we quantitated behavioral changes over lifespan (Figures 4, S4, S5). We find that Wave initiation rate (Figure 4A), Travel speed (Figure 4H), Brush stroke (Figure 4I), and Activity index (Figure 4J) decline to a large degree with age, whereas Body wave number (Figure 4B), Asymmetry (Figure 4C) and Curling (Figure 4G) increase with age. Stretch (Figure 4D) and Reverse swimming (Figure 4F) increase only modestly and Attenuation (Figure 4E) changes relatively little over adult life. These data identify specific measures as having the greatest dynamic range for measurement of age-associated locomotory decline.

We also compared same chronological age *C. elegans* populations judged to have aged well (by crawling assessment and life expectancy tests; Class A [20]) to those judged to have aged badly (Class C) (Figure 4K–M; full study in Figure S5). We find that Class A animals maintain youthful profiles for Activity index (Figure 4K), Wave initiation rate, Brush stroke, Travel speed, Asymmetry (Figure 4L), Body wave number and Curling, but are not greatly changed for Attenuation (Figure 4M). Class C animals score similarly to extremely aged animals for these parameters. Our data validate the use of specific CeleST measures as indicators of quality of locomotory healthspan.

We also examined swim behavior in insulin pathway mutants that are long-lived (reduction-of-function mutations in the *age-1* PI3 kinase) or progeric (*daf-16*/FOXO) [39]–[44]. CeleST analysis supports that *age-1* mutants maintain swimming prowess later into lifespan than WT and *daf-16* mutants are diminished in swim features, especially as compared later in life (Figure 4N, full study in Figure S6). At the same time, data also reveal previously unappreciated details of locomotory phenotypes. For example, by several measures the *age-1* mutant swims more robustly than WT in early adult life—indicating that this mutant begins adulthood with more overall vigor than WT. Since *age-1* enters adult life at a higher capacity level, part of its apparent high maintenance phenotype during aging may derive from its high starting point, a mechanistic aspect that has not previously been considered.

## Availability and Future Directions

We report mathematically-based, automated, high-resolution, high-throughput measures for quantitative description of *C. elegans* swim behavior, which convey novel information on wild-type and mutant swimming locomotion, and report on behavioral change. We describe ten measures that are each designed to quantify a specific aspect of swimming behavior, with a mathematical justification for each. These measures operate over a large range of scales, and report from instantaneous behavior to large temporal patterns, and from single animal analysis to the statistical comparison of thousands of animals throughout their lifespans. The source code together with demonstrations of CeleST is available as Dataset S1 and on http://celest.mbb.rutgers.edu. The source code is distributed under the MIT license as open source. It was developed under MATLAB 2011 with Statistics toolbox. There is no additional pre-requisite to run our program on a computer other than the availability of MATLAB for the specific operating system.

### Advances in analysis of swim motion

While crawling motions have been successfully defined and measured [4]–[11], broadly applied approaches for more complex swimming motions are not well described in the literature. Previous work based on pixel counts [25]–[27] or curvature measures [14], [15] have definitively advanced capacity to evaluate swim locomotion. However, the most sophisticated approaches available to date stop short of automated analysis programs that are applicable to all animal postures and lack measurements that have a precise meaning in terms of behavior. A current common practice is to manually screen and reject videos or data from animals with irregular swim patterns because existing measures cannot address changes within a behavior [14], [22], [23]. By calculating curvature scores along the body over time and using short-time Fourier transform to describe the range of body movements that occur during swims, we have mathematically defined a set of measures that can be directly interpreted in terms of phenotype and swimming behavior. Using curvature score-based calculations, we can evaluate irregular swim patterns in the same way as regular ones, and we can score a broad range of body changes as they occur during a swim trial. This introduces the capacity to track dynamics of a swim as well as changes in pattern, which have not been features of previous programs.

### A design for broad application in research labs

An important feature of CeleST is that the program is designed to run with work-horse instrumentation already on hand in many labs. We captured images at 18 frames/second with image resolution of 0.02 mm/pixel, which does not require an expensive camera. Higher image resolution might improve the robustness of the tracking, but would have no impact on measure analysis, as scores are normalized to worm pixel imprint. The tracking program measures animals as they swim in an area limited by a buffer drop on an inexpensive slide, although scoring in a microfluidic chamber can readily be accomplished. CeleST can track multiple animals simultaneously (we use 5/drop but more are possible), and can import data from other trackers to score measures. Moreover, the program can easily run on long timeframes (minutes to hours, only limited by computer memory), as might be of interest for scoring activity - quiescence patterns that initiate after 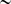
1.5 hours of continuous swimming [12]. Patterns of activity - lower activity/complete inactivity are also readily identified if short-term video is used after a 1.5 hours time point (CIV, data not shown).

### Extendibility

Although we have established a broad baseline for monitoring swim behaviors, we note that the nature of our measures permits custom building of scoring rubrics according to experimental design or phenotype sought. Modest changes could adapt the CeleST program for crawling analysis. Pattern recognition applications and modeling can be added in the future, for example, for in-depth temporal pattern analysis [12] or for social interaction analysis. The CeleST software can be used as a platform, and with application-specific tracking methods and measures, other cellular or animal behaviors can be analyzed (sperm mobility, zebrafish swimming, etc.) for any application that can use video (mating, sleeping, feeding, foraging, etc.).

Overall, the CeleST program constitutes an accessible and comprehensive approach for *C. elegans* locomotory quantitation that translates analysis of complex swimming patterns to a new level of resolution and efficiency. As such, CeleST should be a powerful tool for a high-throughput, high-precision analysis of molecules, neuronal circuits, behavior, and plasticity to advance the effort toward understanding dynamic control of behavior.

## Supporting Information

Figure S1 **Distributions of Wave initiation rates.** We plotted in the form of line histograms the distribution of median Wave initiation rates (WIR) in wild-type (WT) animals as occurs over a 30 second interval. WIR values are binned to integers and the plot line delineates the contour of the bins in the histogram. X axis is median WIR, Y axis is the number of individuals exhibiting the indicated WIR. Data are for age-specific adults: day 4 (A), day 8 (B), day 11 (C), day 15 (D) and day 18 (E), as measured from the hatch. Peaks are positioned at the same median WIR scores over much of adult life. Although the mean WIR (in blue) encompasses a continuum of scores (F), the median (in red) exhibits “preferred” peaks at specific WIR values, unexpectedly revealing that a disproportionate number of animals swim at similar median WIR.
(DOCX)

Figure S2
**Locomotory behavior of** ***glr-1(ky176)*** **adults on days 4 and 11.** Error bars, s.e.m. 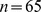 in each data point from 2 independent trials. **A**, Wave initiation rate; **B**, Body wave number; **C**, Asymmetry; **D**, Stretch; **E**, Attenuation; **F**, Reverse swimming; **G**, Curling; **H**, Travel speed; **I**, Brush stroke; and **J**, Activity index. Statistical analysis on pages 2 and 3 follows set of graphs. **ns** indicates not significant; * 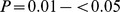; ** 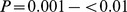; *** 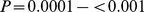; **** 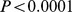.(DOCX)

Figure S3
**Representative lifespan of wild-type control (N2),** ***age-1(hx546)*** **and** ***daf-16 (mgDf50)*** **aging mutants.** Wild type in blue (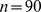, 1 censored), *age-1(hx546)* in green (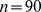, 0 censored) and *daf-16(mgDf50)* in red (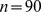, 6 censored). Censored animals were removed from the lifespan data set as they were lost from the plate via desiccation or early-age bursting.(DOCX)

Figure S4
**Age-related locomotory changes in wild-type adults.** Error bars, s.e.m. (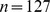 in each data point from 8 independent trials). **A**, Wave initiation rate; **B**, Body wave number; **C**, Asymmetry; **D**, Stretch; **E**, Attenuation; **F**, Reverse swimming; **G**, Curling; **H**, Travel speed; **I**, Brush stroke; and **J**, Activity index. Statistical analysis follows set of graphs. **ns** indicates not significant; * 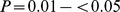; ** 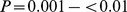; *** 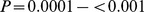; **** 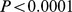.(DOCX)

Figure S5
**Locomotory patterns of same age A and C class adults.** Error bars, s.e.m. (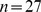 in each data point from two independent trials). **A**, Wave initiation rate; **B**, Body wave number; **C**, Asymmetry; **D**, Stretch; **E**, Attenuation; **F**, Reverse swimming; **G**, Curling; **H**, Travel speed; **I**, Brush stroke; and **J**, Activity index. Statistical analysis follows set of graphs. **ns** indicates not significant; * 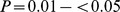; *** 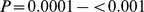; **** 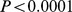. **n/a**, non applicable in cases for curling and reverse swimming parameters that only one animal out of 27 curled or swam in reverse.(DOCX)

Figure S6
**Age-related locomotory changes in wild-type adults (in blue), and in aging mutants** ***age-1(hx546)*** **(in green) and** ***daf-16(mgDf50)*** **(in red).** Error bars, s.e.m. (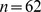 in each data point from four independent trials). **A**, Wave initiation rate; **B**, Body wave number; **C**, Asymmetry; **D**, Stretch; **E**, Attenuation; **F**, Reverse swimming; **G**, Curling; **H**, Travel speed; **I**, Brush stroke; and **J**, Activity index. Statistical analysis follows set of graphs. **ns** indicates not significant; * 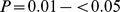; ** 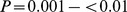; *** 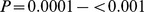; **** 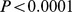.(DOCX)

Table S1
**Comparison of manual and CeleST scores.** **A**, Average of manually scored head bends is analogous to CeleST Wave initiation rate. Sample size (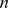) is 108 day 4 adults for manual score of head bends and 101 for automated analysis with CeleST. **B**, CeleST is more sensitive scoring Reverse swimming and Curling, especially for brief events. 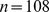 for manual score of Reverse swimming, 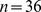 for manual score of Curling, and 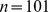 for CeleST automated analysis of Reverse swimming and Curling. All manual scores were executed on a computer screen using recorded videos of the same animals recorded by CeleST. * CeleST automatically measures 10 parameters in a single analysis of one video in about 5 min. The 13.3 min is an estimate of the time CeleST takes to analyze one parameter for 101 animals in 27 videos.(DOCX)

Dataset S1 **Zip package containing source code and demos of the software CeleST.**
(ZIP)

Text S1 **Further details on each of the ten parameter measurements extracted with CeleST (panels C–O of** **Figure 2** **).**
(DOCX)

Text S2 ***C. elegans*** **strains, growth and swim analysis methods.**
(DOCX)
