# Supplementary material for: CeleST: Computer Vision Software for Quantitative Analysis of C. elegans Swim Behavior Reveals Novel Features of Locomotion
Source: PLoS Comput Biol. 2014 Jul 17;10(7):e1003702. doi: 10.1371/journal.pcbi.1003702 (PMC4102393; doi:10.1371/journal.pcbi.1003702)
Supplement: Figure S1 — Distributions of Wave initiation rates. We plotted in the form of line histograms the distribution of median Wave initiation rates (WIR) in wild-type (WT) animals as occurs over a 30 second interval. WIR values are binned to integers and the plot line delineates the contour of the bins in the histogram. X axis is median WIR, Y axis is the number of individuals exhibiting the indicated WIR. Data are for age-specific adults: day 4 (A), day 8 (B), day 11 (C), day 15 (D) and day 18 (E), as measured from the hatch. Peaks are positioned at the same median WIR scores over much of adult life. Although the mean WIR (in blue) encompasses a continuum of scores (F), the median (in red) exhibits “preferred” peaks at specific WIR values, unexpectedly revealing that a disproportionate number of animals swim at similar median WIR. (DOCX) [file pcbi.1003702.s001.docx]

**Figure S1. Distributions of Wave initiation rates.** We plotted in the form of line histograms the distribution of median Wave initiation rates (WIR) in wild-type (WT) animals as occurs over a 30 second interval. WIR values are binned to integers and the plot line delineates the contour of the bins in the histogram. X axis is median WIR, Y axis is the number of individuals exhibiting the indicated WIR. Data are for age-specific adults: day 4 (**A**), day 8 (**B**), day 11 (**C**), day 15 (**D**) and day 18 (**E**), as measured from the hatch. Peaks are positioned at the same median WIR scores over much of adult life. Although the mean WIR (in blue) encompasses a continuum of scores (**F**), the median (in red) exhibits “preferred” peaks at specific WIR values, unexpectedly revealing that a disproportionate number of animals swim at similar median WIR.

**
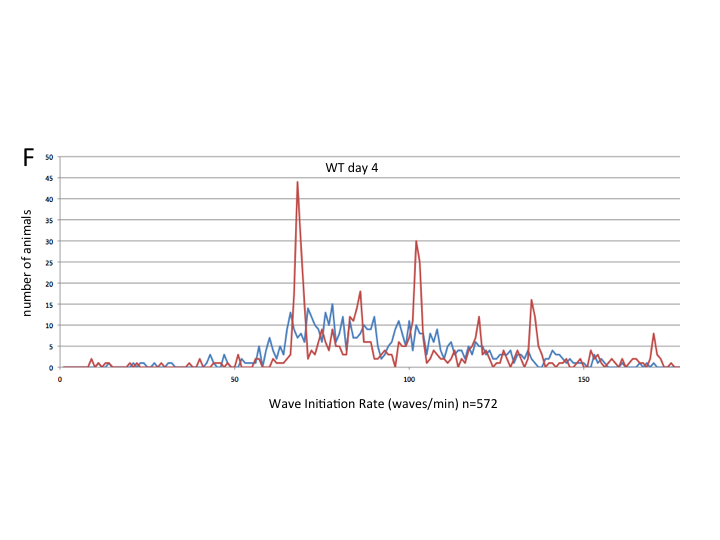
**
