# Supplementary material for: CeleST: Computer Vision Software for Quantitative Analysis of C. elegans Swim Behavior Reveals Novel Features of Locomotion
Source: PLoS Comput Biol. 2014 Jul 17;10(7):e1003702. doi: 10.1371/journal.pcbi.1003702 (PMC4102393; doi:10.1371/journal.pcbi.1003702)
Supplement: Figure S2 — Locomotory behavior of glr-1(ky176) adults on days 4 and 11. Error bars, s.e.m. in each data point from 2 independent trials. A, Wave initiation rate; B, Body wave number; C, Asymmetry; D, Stretch; E, Attenuation; F, Reverse swimming; G, Curling; H, Travel speed; I, Brush stroke; and J, Activity index. Statistical analysis on pages 2 and 3 follows set of graphs. ns indicates not significant; * ; ** ; *** ; **** . (DOCX) [file pcbi.1003702.s002.docx]

**Figure S2. Locomotory behavior of *glr-1(ky176)* adults on days 4 and 11.** Error bars, s.e.m. n=65 in each data point from 2 independent trials. **A**, Wave initiation rate; **B**, Body wave number; **C**, Asymmetry; **D**, Stretch; **E**, Attenuation; **F**, Reverse swimming; **G**, Curling; **H**, Travel speed; **I**, Brush stroke; and **J**, Activity index. Statistical analysis on pages 2 and 3 follows set of graphs. **ns** indicates non significance; *****, *P* = 0.01 – <0.05; ******, *P* = 0.001 – <0.01; *******, *P* = 0.0001 – <0.001; ********, *P* < 0.0001.

**I**

**C**

**A**

**D**

**G**

**J**

**B**

**E**

**F**

**H**

**A Wave initiation rate**

- One-way ANOVA followed by Bonferroni’s multiple comparison test, selected pairs

| **Wave initiation rate** |  |
| --- | --- |
| day 4 WT vs day 4 *glr-1(ky176)* | ns |
| day 11 WT vs day 11 *glr-1(ky176)* | *** |
| day 4 WT vs day 11 WT | **** |
| day 4 *glr-1(ky176)* vs day 11 *glr-1(ky176)* | **** |

**B Body wave wumber**

- One-way ANOVA followed by Bonferroni’s multiple comparison test, selected pairs

| **Body wave number** |  |
| --- | --- |
| day 4 WT vs day 4 *glr-1(ky176)* | ns |
| day 11 WT vs day 11 *glr-1(ky176)* | ns |
| day 4 WT vs day 11 WT | **** |
| day 4 *glr-1(ky176)* vs day 11 *glr-1(ky176)* | **** |

**C Asymmetry**

- One-way ANOVA followed by Bonferroni’s multiple comparison test, selected pairs

| **Asymmetry** |  |
| --- | --- |
| day 4 WT vs day 4 *glr-1(ky176)* | *** |
| day 11 WT vs day 11 *glr-1(ky176)* | ns |
| day 4 WT vs day 11 WT | ns |
| day 4 *glr-1(ky176)* vs day 11 *glr-1(ky176)* | *** |

**D Stretch**

- One-way ANOVA followed by Bonferroni’s multiple comparison test, selected pairs

| **Stretch** |  |
| --- | --- |
| day 4 WT vs day 4 *glr-1(ky176)* | ns |
| day 4 WT vs day 11 WT | ns |
| day 4 *glr-1(ky176)* vs day 11 *glr-1(ky176)* | ns |
| day 11 WT vs day 11 *glr-1(ky176)* | ns |

**E Attenuation**

- One-way ANOVA followed by Bonferroni’s multiple comparison test, selected pairs

| **Attenuation** |  |
| --- | --- |
| day 4 WT vs day 4 *glr-1(ky176)* | *** |
| day 11 WT vs day 11 *glr-1(ky176)* | ns |
| day 4 WT vs day 11 WT | ns |
| day 4 *glr-1(ky176)* vs day 11 *glr-1(ky176)* | * |

**F Reverse swimming**

- One-way ANOVA followed by Bonferroni’s multiple comparison test, selected pairs

| **Reverse swimming** |  |
| --- | --- |
| day 4 WT vs day 4 *glr-1(ky176)* | ns |
| day 11 WT vs day 11 *glr-1(ky176)* | ns |
| day 4 WT vs day 11 WT | ns |
| day 4 *glr-1(ky176)* vs day 11 *glr-1(ky176)* | ns |

**G Curling**

- One-way ANOVA followed by Bonferroni’s multiple comparison test, selected pairs

| **Curling** |  |
| --- | --- |
| day 4 WT vs day 4 *glr-1(ky176)* | ns |
| day 11 WT vs day 11 *glr-1(ky176)* | ns |
| day 4 WT vs day 11 WT | ** |
| day 4 *glr-1(ky176)* vs day 11 *glr-1(ky176)* | *** |

**H Travel speed**

- One-way ANOVA followed by Bonferroni’s multiple comparison test, selected pairs

| **Travel speed** |  |
| --- | --- |
| day 4 WT vs day 4 *glr-1(ky176)* | ns |
| day 11 WT vs day 11 *glr-1(ky176)* | ** |
| day 4 WT vs day 11 WT | **** |
| day 4 *glr-1(ky176)* vs day 11 *glr-1(ky176)* | **** |

**I Brush stroke**

- One-way ANOVA followed by Bonferroni’s multiple comparison test, selected pairs

| **Brush stroke** |  |
| --- | --- |
| day 4 WT vs day 4 *glr-1(ky176)* | ns |
| day 11 WT vs day 11 *glr-1(ky176)* | ** |
| day 4 WT vs day 11 WT | **** |
| day 4 *glr-1(ky176)* vs day 11 *glr-1(ky176)* | **** |

**J Activity index**

- One-way ANOVA followed by Bonferroni’s multiple comparison test, selected pairs

| **Activity index** |  |
| --- | --- |
| day 4 WT vs day 4 *glr-1(ky176)* | ns |
| day 11 WT vs day 11 *glr-1(ky176)* | * |
| day 4 WT vs day 11 WT | **** |
| day 4 *glr-1(ky176)* vs day 11 *glr-1(ky176)* | **** |
