# Supplementary material for: CeleST: Computer Vision Software for Quantitative Analysis of C. elegans Swim Behavior Reveals Novel Features of Locomotion
Source: PLoS Comput Biol. 2014 Jul 17;10(7):e1003702. doi: 10.1371/journal.pcbi.1003702 (PMC4102393; doi:10.1371/journal.pcbi.1003702)
Supplement: Figure S3 — Representative lifespan of wild-type control (N2), age-1(hx546) and daf-16 (mgDf50) aging mutants. Wild type in blue (, 1 censored), age-1(hx546) in green (, 0 censored) and daf-16(mgDf50) in red (, 6 censored). Censored animals were removed from the lifespan data set as they were lost from the plate via desiccation or early-age bursting. (DOCX) [file pcbi.1003702.s003.docx]

**Figure S3. Representative lifespan of wild-type control (N2), *age-1 (hx546)* and *daf-16 (mgDf50)* aging mutants.** Wild type in blue (n=90, 1 censored), *age-1(hx546)* in green (n=90, 0 censored) and *daf-16(mgDf50)* in red (n= 90, 6 censored). Censored animals were removed from the lifespan data set as they were lost from the plate via desiccation or early-age bursting.
