# Supplementary material for: CeleST: Computer Vision Software for Quantitative Analysis of C. elegans Swim Behavior Reveals Novel Features of Locomotion
Source: PLoS Comput Biol. 2014 Jul 17;10(7):e1003702. doi: 10.1371/journal.pcbi.1003702 (PMC4102393; doi:10.1371/journal.pcbi.1003702)
Supplement: Figure S4 — Age-related locomotory changes in wild-type adults. Error bars, s.e.m. ( in each data point from 8 independent trials). A, Wave initiation rate; B, Body wave number; C, Asymmetry; D, Stretch; E, Attenuation; F, Reverse swimming; G, Curling; H, Travel speed; I, Brush stroke; and J, Activity index. Statistical analysis follows set of graphs. ns indicates not significant; * ; ** ; *** ; **** . (DOCX) [file pcbi.1003702.s004.docx]

**Figure S4. Age-related locomotory changes in wild-type adults.** Error bars, s.e.m. (n=127 in each data point from 8 independent trials). **A**, Wave initiation rate; **B**, Body wave number; **C**, Asymmetry; **D**, Stretch; **E**, Attenuation; **F**, Reverse swimming; **G**, Curling; **H**, Travel speed; **I**, Brush stroke; and **J**, Activity index. Statistical analysis follows set of graphs. **ns** indicates non significance; *****, *P* = 0.01 – <0.05; ******, *P* = 0.001 – <0.01; *******, *P* = 0.0001 – <0.001; ********, *P* < 0.0001.

**I**

**C**

**A**

**D**

**G**

**J**

**B**

**E**

**F**

**H**

**A Wave initiation rate**

- One-way ANOVA, followed by Tukey’s multiple comparison test

| **Wave initiation rate** | day 6 | day 8 | day 11 | day 13 | day 15 | day 18 | day 20 |
| --- | --- | --- | --- | --- | --- | --- | --- |
| day 4 | ns | *** | *** | *** | *** | *** | *** |
| day 6 |  | ns | *** | *** | *** | *** | *** |
| day 8 |  |  | *** | *** | *** | *** | *** |
| day 11 |  |  |  | ns | ns | ns | ns |
| day 13 |  |  |  |  | ns | ns | ns |
| day 15 |  |  |  |  |  | ns | ns |
| day 18 |  |  |  |  |  |  | ns |

- One-way ANOVA followed by Bonferroni’s multiple comparison test, selected pairs

| **Wave initiation rate** |  |
| --- | --- |
| day 4 vs day 6 | ns |
| day 6 vs day 8 | ns |
| day 8 vs day 11 | **** |
| day 11 vs day 13 | ns |
| day 13 vs day 15 | ns |
| day 15 vs day 18 | * |
| day 18 vs day 20 | ns |

**B Body wave number**

- One-way ANOVA followed by Tukey’s multiple comparison test

| **Body wave number** | day 6 | day 8 | day 11 | day 13 | day 15 | day 18 | day 20 |
| --- | --- | --- | --- | --- | --- | --- | --- |
| day 4 | ns | ns | *** | *** | *** | *** | *** |
| day 6 |  | ns | *** | *** | *** | *** | *** |
| day 8 |  |  | *** | *** | *** | *** | *** |
| day 11 |  |  |  | ns | ns | * | ns |
| day 13 |  |  |  |  | ns | ns | ns |
| day 15 |  |  |  |  |  | ns | ns |
| day 18 |  |  |  |  |  |  | * |

- One-way ANOVA followed by Bonferroni’s multiple comparison test, selected pairs

| **Body wave number** |  |
| --- | --- |
| day 4 vs day 6 | ns |
| day 6 vs day 8 | ns |
| day 8 vs day 11 | **** |
| day 11 vs day 13 | ns |
| day 13 vs day 15 | ns |
| day 15 vs day 18 | ns |
| day 18 vs day 20 | ** |

**C Asymmetry**

- One-way ANOVA followed by Tukey’s multiple comparison test

| **Asymmetry** | day 6 | day 8 | day 11 | day 13 | day 15 | day 18 | day 20 |
| --- | --- | --- | --- | --- | --- | --- | --- |
| day 4 | ns | ns | ** | * | ns | ** | *** |
| day 6 |  | ns | * | ns | ns | * | *** |
| day 8 |  |  | ns | ns | ns | ns | ** |
| day 11 |  |  |  | ns | ns | ns | ns |
| day 13 |  |  |  |  | ns | ns | ns |
| day 15 |  |  |  |  |  | ns | * |
| day 18 |  |  |  |  |  |  | ns |

- One-way ANOVA followed by Bonferroni’s multiple comparison test, selected pairs

| **Asymmetry** |  |
| --- | --- |
| day 4 vs day 6 | ns |
| day 6 vs day 8 | ns |
| day 8 vs day 11 | ns |
| day 11 vs day 13 | ns |
| day 13 vs day 15 | ns |
| day 15 vs day 18 | ns |
| day 18 vs day 20 | ns |

**D Stretch**

- One-way ANOVA followed by Tukey’s multiple comparison test

| **Stretch** | day 6 | day 8 | day 11 | day 13 | day 15 | day 18 | day 20 |
| --- | --- | --- | --- | --- | --- | --- | --- |
| day 4 | ns | ns | ns | ns | ** | ** | *** |
| day 6 |  | ns | ns | ns | ns | ns | *** |
| day 8 |  |  | ns | ns | ns | ns | * |
| day 11 |  |  |  | ns | ** | ** | *** |
| day 13 |  |  |  |  | ns | ns | *** |
| day 15 |  |  |  |  |  | ns | ns |
| day 18 |  |  |  |  |  |  | ns |

- One-way ANOVA followed by Bonferroni’s multiple comparison test, selected pairs

| **Stretch** |  |
| --- | --- |
| day 4 vs day 6 | ns |
| day 6 vs day 8 | ns |
| day 8 vs day 11 | ns |
| day 11 vs day 13 | ns |
| day 13 vs day 15 | ns |
| day 15 vs day 18 | ns |
| day 18 vs day 20 | ns |

**E Attenuation**

- One-way ANOVA followed by Tukey’s multiple comparison test

| **Attenuation** | day 6 | day 8 | day 11 | day 13 | day 15 | day 18 | day 20 |
| --- | --- | --- | --- | --- | --- | --- | --- |
| day 4 | ns | ns | ns | ns | ns | ns | ns |
| day 6 |  | ns | ns | ns | ns | ns | ns |
| day 8 |  |  | ns | ns | ns | ns | ns |
| day 11 |  |  |  | ns | ns | ns | ns |
| day 13 |  |  |  |  | ns | ns | ns |
| day 15 |  |  |  |  |  | ns | ns |
| day 18 |  |  |  |  |  |  | ns |

- One-way ANOVA followed by Bonferroni’s multiple comparison test, selected pairs

| **Attenuation** |  |
| --- | --- |
| day 4 vs day 6 | ns |
| day 6 vs day 8 | ns |
| day 8 vs day 11 | ns |
| day 11 vs day 13 | ns |
| day 13 vs day 15 | ns |
| day 15 vs day 18 | ns |
| day 18 vs day 20 | ns |

**F Reverse swimming**

- One-way ANOVA followed by Tukey’s multiple comparison test

| **Reverse swimming** | day 6 | day 8 | day 11 | day 13 | day 15 | day 18 | day 20 |
| --- | --- | --- | --- | --- | --- | --- | --- |
| day 4 | ns | ns | ns | ns | ns | ns | ns |
| day 6 |  | ns | ns | ns | ns | ns | ns |
| day 8 |  |  | ns | ns | ns | ns | ns |
| day 11 |  |  |  | ns | ns | ns | ns |
| day 13 |  |  |  |  | ns | ns | ns |
| day 15 |  |  |  |  |  | ns | ns |
| day 18 |  |  |  |  |  |  | ns |

- One-way ANOVA followed by Bonferroni’s multiple comparison test, selected pairs

| **Reserve swimming** |  |
| --- | --- |
| day 4 vs day 6 | ns |
| day 6 vs day 8 | ns |
| day 8 vs day 11 | ns |
| day 11 vs day 13 | ns |
| day 13 vs day 15 | ns |
| day 15 vs day 18 | ns |
| day 18 vs day 20 | ns |

**G Curling**

- One-way ANOVA followed by Tukey’s multiple comparison test

| **Curling** | day 6 | day 8 | day 11 | day 13 | day 15 | day 18 | day 20 |
| --- | --- | --- | --- | --- | --- | --- | --- |
| day 4 | ns | ns | ** | * | ** | ns | *** |
| day 6 |  | ns | ns | ns | ns | ns | *** |
| day 8 |  |  | ns | ns | ns | ns | *** |
| day 11 |  |  |  | ns | ns | ns | ns |
| day 13 |  |  |  |  | ns | ns | ** |
| day 15 |  |  |  |  |  | ns | ** |
| day 18 |  |  |  |  |  |  | ** |

- One-way ANOVA followed by Bonferroni’s multiple comparison test, selected pairs

| **Curling** |  |
| --- | --- |
| day 4 vs day 6 | ns |
| day 6 vs day 8 | ns |
| day 8 vs day 11 | ns |
| day 11 vs day 13 | ns |
| day 13 vs day 15 | ns |
| day 15 vs day 18 | ns |
| day 18 vs day 20 | *** |

**H Travel speed**

- One-way ANOVA followed by Tukey’s multiple comparison test

| **Travel speed** | day 6 | day 8 | day 11 | day 13 | day 15 | day 18 | day 20 |
| --- | --- | --- | --- | --- | --- | --- | --- |
| day 4 | ns | * | *** | *** | *** | *** | *** |
| day 6 |  | ns | *** | *** | *** | *** | *** |
| day 8 |  |  | *** | *** | *** | *** | *** |
| day 11 |  |  |  | ns | ns | ns | ns |
| day 13 |  |  |  |  | ns | ns | ns |
| day 15 |  |  |  |  |  | * | ns |
| day 18 |  |  |  |  |  |  | ns |

- One-way ANOVA followed by Bonferroni’s multiple comparison test, selected pairs

| **Travel speed** |  |
| --- | --- |
| day 4 vs day 6 | ns |
| day 6 vs day 8 | ns |
| day 8 vs day 11 | **** |
| day 11 vs day 13 | ns |
| day 13 vs day 15 | ns |
| day 15 vs day 18 | * |
| day 18 vs day 20 | ns |

**I Brush stroke**

- One-way ANOVA followed by Tukey’s multiple comparison test

| **Brush stroke** | day 6 | day 8 | day 11 | day 13 | day 15 | day 18 | day 20 |
| --- | --- | --- | --- | --- | --- | --- | --- |
| day 4 | ns | *** | *** | *** | *** | *** | *** |
| day 6 |  | ns | *** | *** | *** | *** | *** |
| day 8 |  |  | *** | *** | *** | *** | *** |
| day 11 |  |  |  | ns | ns | ns | ns |
| day 13 |  |  |  |  | ns | ns | ns |
| day 15 |  |  |  |  |  | * | ns |
| day 18 |  |  |  |  |  |  | ns |

- One-way ANOVA followed by Bonferroni’s multiple comparison test, selected pairs

| **Brush stroke** |  |
| --- | --- |
| day 4 vs day 6 | ns |
| day 6 vs day 8 | * |
| day 8 vs day 11 | **** |
| day 11 vs day 13 | ns |
| day 13 vs day 15 | ns |
| day 15 vs day 18 | ** |
| day 18 vs day 20 | ns |

**J** **Activity index**

- One-way ANOVA followed by Tukey’s multiple comparison test

| **Activity index** | day 6 | day 8 | day 11 | day 13 | day 15 | day 18 | day 20 |
| --- | --- | --- | --- | --- | --- | --- | --- |
| day 4 | ns | * | *** | *** | *** | *** | *** |
| day 6 |  | ns | *** | *** | *** | *** | *** |
| day 8 |  |  | *** | *** | *** | *** | *** |
| day 11 |  |  |  | ns | ns | ns | ns |
| day 13 |  |  |  |  | ns | ns | ns |
| day 15 |  |  |  |  |  | ns | ns |
| day 18 |  |  |  |  |  |  | ns |

- One-way ANOVA followed by Bonferroni’s multiple comparison test, selected pairs

| **Activity index** |  |
| --- | --- |
| day 4 vs day 6 | ns |
| day 6 vs day 8 | ns |
| day 8 vs day 11 | **** |
| day 11 vs day 13 | ns |
| day 13 vs day 15 | ns |
| day 15 vs day 18 | ns |
| day 18 vs day 20 | ns |
