# Supplementary material for: CeleST: Computer Vision Software for Quantitative Analysis of C. elegans Swim Behavior Reveals Novel Features of Locomotion
Source: PLoS Comput Biol. 2014 Jul 17;10(7):e1003702. doi: 10.1371/journal.pcbi.1003702 (PMC4102393; doi:10.1371/journal.pcbi.1003702)
Supplement: Figure S5 — Locomotory patterns of same age A and C class adults. Error bars, s.e.m. ( in each data point from two independent trials). A, Wave initiation rate; B, Body wave number; C, Asymmetry; D, Stretch; E, Attenuation; F, Reverse swimming; G, Curling; H, Travel speed; I, Brush stroke; and J, Activity index. Statistical analysis follows set of graphs. ns indicates not significant; * ; *** ; **** . n/a, non applicable in cases for curling and reverse swimming parameters that only one animal out of 27 curled or swam in reverse. (DOCX) [file pcbi.1003702.s005.docx]

**Figure S5. Locomotory patterns of same age A and C class adults.** Error bars, s.e.m. (n=27 in each data point from two independent trials). (A) Wave initiation rate, (B) Body wave number, (C) Asymmetry, (D) Stretch, (E) Attenuation, (F) Reverse swimming, (G) Curling, (H) Travel speed, (I) Brush stroke and (J) Activity index. Statistical analysis follows set of graphs. **ns** indicates non significance; *****, *P* = 0.01 – <0.05; *******, *P* = 0.0001 – <0.001; ********, *P* < 0.0001. **n/a**, non applicable in cases for curling and reverse swimming parameters that only one animal out of 27 curled or swam reversing.

**I**

**C**

**A**

**D**

**G**

**J**

**B**

**E**

**F**

**H**

**A Wave initiation rate**

- One-way ANOVA, followed by Dunnett’s multiple comparison test

| **Wave initiation rate** |  |
| --- | --- |
| day 4 vs class A_day 10 | ns |
| day 4 vs class A_day 11 | ns |
| day 4 vs class C_day 10 | *** |
| day 4 vs class C_day 11 | *** |

- Unpaired t test with Welch’s correction

| **Wave initiation rate** |  |
| --- | --- |
| class A_day 10 vs class C_day 10 | **** |
| class A_day 11 vs class C_day 11 | **** |

**B Body wave number**

- One-way ANOVA, followed by Dunnett’s multiple comparison test

| **Body wave number** |  |
| --- | --- |
| day 4 vs class A_day 10 | *** |
| day 4 vs class A_day 11 | *** |
| day 4 vs class C_day 10 | *** |
| day 4 vs class C_day 11 | *** |

- Unpaired t test with Welch’s correction

| **Body wave number** |  |
| --- | --- |
| class A_day 10 vs class C_day 10 | **** |
| class A_day 11 vs class C_day 11 | **** |

**C Asymmetry**

- One-way ANOVA, followed by Dunnett’s multiple comparison test

| **Asymmetry** |  |
| --- | --- |
| day 4 vs class A_day 10 | ns |
| day 4 vs class A_day 11 | ns |
| day 4 vs class C_day 10 | *** |
| day 4 vs class C_day 11 | *** |

- Unpaired t test with Welch’s correction

| **Asymmetry** |  |
| --- | --- |
| class A_day 10 vs class C_day 10 | **** |
| class A_day 11 vs class C_day 11 | **** |

**D Stretch**

- One-way ANOVA, followed by Dunnett’s multiple comparison test

| **Stretch** |  |
| --- | --- |
| day 4 vs class A_day 10 | ns |
| day 4 vs class A_day 11 | ns |
| day 4 vs class C_day 10 | ns |
| day 4 vs class C_day 11 | ns |

- Unpaired t test with Welch’s correction

| **Stretch** |  |
| --- | --- |
| class A_day 10 vs class C_day 10 | ns |
| class A_day 11 vs class C_day 11 | ns |

**E Attenuation**

- One-way ANOVA, followed by Dunnett’s multiple comparison test

| **Attenuation** |  |
| --- | --- |
| day 4 vs class A_day 10 | *** |
| day 4 vs class A_day 11 | *** |
| day 4 vs class C_day 10 | ns |
| day 4 vs class C_day 11 | ns |

- Unpaired t test with Welch’s correction

| **Attenuation** |  |
| --- | --- |
| class A_day 10 vs class C_day 10 | ns |
| class A_day 11 vs class C_day 11 | ns |

**F Reverse swimming**

- One-way ANOVA, followed by Dunnett’s multiple comparison test

| **Reverse swimming** |  |
| --- | --- |
| day 4 vs class A_day 10 | ns |
| day 4 vs class A_day 11 | ns |
| day 4 vs class C_day 10 | n/a |
| day 4 vs class C_day 11 | n/a |

- Unpaired t test with Welch’s correction

| **Reverse swimming** |  |
| --- | --- |
| class A_day 10 vs class C_day 10 | ns |
| class A_day 11 vs class C_day 11 | n/a |

**G Curling**

- One-way ANOVA, followed by Dunnett’s multiple comparison test

| **Curling** |  |
| --- | --- |
| day 4 vs class A_day 10 | ns |
| day 4 vs class A_day 11 | ns |
| day 4 vs class C_day 10 |  |
| day 4 vs class C_day 11 | n/a |

- Unpaired t test with Welch’s correction

| **Curling** |  |
| --- | --- |
| class A_day 10 vs class C_day 10 | ** |
| class A_day 11 vs class C_day 11 | n/a |

**H Travel speed**

- One-way ANOVA, followed by Dunnett’s multiple comparison test

| **Travel speed** |  |
| --- | --- |
| day 4 vs class A_day 10 | ns |
| day 4 vs class A_day 11 | * |
| day 4 vs class C_day 10 | *** |
| day 4 vs class C_day 11 | *** |

- Unpaired t test with Welch’s correction

| **Travel speed** |  |
| --- | --- |
| class A_day 10 vs class C_day 10 | **** |
| class A_day 11 vs class C_day 11 | **** |

**I Brush stroke**

- One-way ANOVA, followed by Dunnett’s multiple comparison test

| **Brush stroke** |  |
| --- | --- |
| day 4 vs class A_day 10 | ns |
| day 4 vs class A_day 11 | ns |
| day 4 vs class C_day 10 | *** |
| day 4 vs class C_day 11 | *** |

- Unpaired t test with Welch’s correction

| **Brush stroke** |  |
| --- | --- |
| class A_day 10 vs class C_day 10 | **** |
| class A_day 11 vs class C_day 11 | **** |

**J Activity index**

- One-way ANOVA, followed by Dunnett’s multiple comparison test

| **Activity index** |  |
| --- | --- |
| day 4 vs class A_day 10 | ns |
| day 4 vs class A_day 11 | ns |
| day 4 vs class C_day 10 | *** |
| day 4 vs class C_day 11 | *** |

- Unpaired t test with Welch’s correction

| **Activity index** |  |
| --- | --- |
| class A_day 10 vs class C_day 10 | **** |
| class A_day 11 vs class C_day 11 | **** |
