# Supplementary material for: CeleST: Computer Vision Software for Quantitative Analysis of C. elegans Swim Behavior Reveals Novel Features of Locomotion
Source: PLoS Comput Biol. 2014 Jul 17;10(7):e1003702. doi: 10.1371/journal.pcbi.1003702 (PMC4102393; doi:10.1371/journal.pcbi.1003702)
Supplement: Figure S6 — Age-related locomotory changes in wild-type adults (in blue), and in aging mutants age-1(hx546) (in green) and daf-16(mgDf50) (in red). Error bars, s.e.m. ( in each data point from four independent trials). A, Wave initiation rate; B, Body wave number; C, Asymmetry; D, Stretch; E, Attenuation; F, Reverse swimming; G, Curling; H, Travel speed; I, Brush stroke; and J, Activity index. Statistical analysis follows set of graphs. ns indicates not significant; * ; ** ; *** ; **** . (DOCX) [file pcbi.1003702.s006.docx]

**Figure S6. Age-related locomotory changes in wild-type adults (in blue), and in aging mutants *age-1(hx546)* (in green) and *daf-16(mgDf50)* (in red).** Error bars, s.e.m. (n=62 in each data point from four independent trials). (A) Wave initiation rate, (B) Body wave number, (C) Asymmetry, (D) Stretch, (E) Attenuation, (F) Reverse swimming, (G) Curling, (H) Travel speed, (I) Brush stroke and (J) Activity index. Statistical analysis follows set of graphs. **ns** indicates non significance; *, *P* = 0.01 – <0.05; ******, *P* = 0.001 – <0.01; *******, *P* = 0.0001 – <0.001; ********, *P* < 0.0001.

**I**

**C**

**A**

**D**

**G**

**J**

**B**

**E**

**F**

**H**

**A Wave initiation rate**

- One-way ANOVA, followed by Dunnett’s multiple comparison test

| **Wave initiation rate** | |  |
| --- | --- | --- |
| day 4 | WT vs *age-1(hx546)* | *** |
|  | WT vs *daf-16(mgDf50)* | ns |
| day 6 | WT vs *age-1(hx546)* | *** |
|  | WT vs *daf-16(mgDf50)* | ns |
| day 8 | WT vs *age-1(hx546)* | *** |
|  | WT vs *daf-16(mgDf50)* | ns |
| day 11 | WT vs *age-1(hx546)* | *** |
|  | WT vs *daf-16(mgDf50)* | ns |
| day 13 | WT vs *age-1(hx546)* | ns |
|  | WT vs *daf-16(mgDf50)* | *** |
| day 15 | WT vs *age-1(hx546)* | * |
|  | WT vs *daf-16(mgDf50)* | *** |
| day 18 | WT vs *age-1(hx546)* | ns |
|  | WT vs *daf-16(mgDf50)* | * |
| day 20 | WT vs *age-1(hx546)* | ns |
|  | WT vs *daf-16(mgDf50)* | *** |

- One-way ANOVA, followed by Bonferroni’s multiple comparison test

| **Wave initiation rate** |  |
| --- | --- |
| day 4 WT vs day 6 WT | ns |
| day 6 WT vs day 8 WT | ns |
| day 8 WT vs day 11 WT | ** |
| day 11 WT vs day 13 WT | ** |
| day 13 WT vs day 15 WT | ns |
| day 15 WT vs day 18 WT | *** |
| day 18 WT vs day 20 WT | ns |
| day 4 *age-1(hx546)* vs *day 6 age-1(hx546)* | ns |
| day 6 *age-1(hx546)* vs day 8 *age-1(hx546)* | ns |
| day 8 *age-1(hx546)* vs day 11 *age-1(hx546)* | ns |
| day 11 *age-1(hx546)* vs day 13 *age-1(hx546)* | ns |
| day 13 *age-1(hx546)* vs day 15 *age-1(hx546)* | * |
| day 15 *age-1(hx546)* vs day 18 *age-1(hx546)* | **** |
| day 18 *age-1(hx546)* vs day 20 *age-1(hx546)* | ns |
| day 4 *daf-16(mgDf50)* vs day 6 *daf-16(mgDf50)* | ns |
| day 6 *daf-16(mgDf50)* vs day 8 *daf-16(mgDf50)* | ns |
| day 8 *daf-16(mgDf50)* vs day 11 *daf-16(mgDf50)* | *** |
| day 11 *daf-16(mgDf50)* vs day 13 *daf-16(mgDf50)* | ns |
| day 13 *daf-16(mgDf50)* vs day 15 *daf-16(mgDf50)* | *** |
| day 15 *daf-16(mgDf50)* vs day 18 *daf-16(mgDf50)* | ns |
| day 18 *daf-16(mgDf50)* vs day 20 *daf-16(mgDf50)* | ns |

**B Body wave number**

- One-way ANOVA, followed by Dunnett’s multiple comparison test

| **Body wave number** | |  |
| --- | --- | --- |
| day 4 | WT vs *age-1(hx546)* | ns |
|  | WT vs *daf-16(mgDf50)* | ns |
| day 6 | WT vs *age-1(hx546)* | ns |
|  | WT vs *daf-16(mgDf50)* | ns |
| day 8 | WT vs *age-1(hx546)* | ns |
|  | WT vs *daf-16(mgDf50)* | ns |
| day 11 | WT vs *age-1(hx546)* | * |
|  | WT vs *daf-16(mgDf50)* | ns |
| day 13 | WT vs *age-1(hx546)* | ns |
|  | WT vs *daf-16(mgDf50)* | ns |
| day 15 | WT vs *age-1(hx546)* | ns |
|  | WT vs *daf-16(mgDf50)* | *** |
| day 18 | WT vs *age-1(hx546)* | * |
|  | WT vs *daf-16(mgDf50)* | ns |
| day 20 | WT vs *age-1(hx546)* | ns |
|  | WT vs *daf-16(mgDf50)* | *** |

- One-way ANOVA, followed by Bonferroni’s multiple comparison test

| **Body wave number** |  |
| --- | --- |
| day 4 WT vs day 6 WT | ns |
| day 6 WT vs day 8 WT | ns |
| day 8 WT vs day 11 WT | **** |
| day 11 WT vs day 13 WT | ns |
| day 13 WT vs day 15 WT | ns |
| day 15 WT vs day 18 WT | *** |
| day 18 WT vs day 20 WT | **** |
| day 4 *age-1(hx546)* vs day 6 *age-1(hx546)* | ns |
| day 6 *age-1(hx546)* vs day 8 *age-1(hx546)* | ns |
| day 8 *age-1(hx546)* vs day 11 *age-1(hx546)* | ns |
| day 11 *age-1(hx546)* vs day 13 *age-1(hx546)* | ** |
| day 13 *age-1(hx546)* vs day 15 *age-1(hx546)* | ns |
| day 15 *age-1(hx546)* vs day 18 *age-1(hx546)* | ns |
| day 18 *age-1(hx546)* vs day 20 *age-1(hx546)* | ns |
| day 4 *daf-16(mgDf50)* vs day 6 *daf-16(mgDf50)* | ns |
| day 6 *daf-16(mgDf50)* vs day 8 *daf-16(mgDf50)* | ns |
| day 8 *daf-16(mgDf50)* vs day 11 *daf-16(mgDf50)* | ** |
| day 11 *daf-16(mgDf50)* vs day 13 *daf-16(mgDf50)* | ns |
| day 13 *daf-16(mgDf50)* vs day 15 *daf-16(mgDf50)* | **** |
| day 15 *daf-16(mgDf50)* vs day 18 *daf-16(mgDf50)* | ns |
| day 18 *daf-16(mgDf50)* vs day 20 *daf-16(mgDf50)* | ns |

**C Asymmetry**

- One-way ANOVA, followed by Dunnett’s multiple comparison test

| **Asymmetry** | |  |
| --- | --- | --- |
| day 4 | WT vs *age-1(hx546)* | ns |
|  | WT vs *daf-16(mgDf50)* | * |
| day 6 | WT vs *age-1(hx546)* | ns |
|  | WT vs *daf-16(mgDf50)* | ns |
| day 8 | WT vs *age-1(hx546)* | *** |
|  | WT vs *daf-16(mgDf50)* | ** |
| day 11 | WT vs *age-1(hx546)* | ns |
|  | WT vs *daf-16(mgDf50)* | ns |
| day 13 | WT vs *age-1(hx546)* | ns |
|  | WT vs *daf-16(mgDf50)* | ns |
| day 15 | WT vs *age-1(hx546)* | ns |
|  | WT vs *daf-16(mgDf50)* | *** |
| day 18 | WT vs *age-1(hx546)* | ns |
|  | WT vs *daf-16(mgDf50)* | ns |
| day 20 | WT vs *age-1(hx546)* | ** |
|  | WT vs *daf-16(mgDf50)* | ns |

- One-way ANOVA, followed by Bonferroni’s multiple comparison test

| **Asymmetry** |  |
| --- | --- |
| day 4 WT vs day 6 WT | ns |
| day 6 WT vs day 8 WT | ns |
| day 8 WT vs day 11 WT | ns |
| day 11 WT vs day 13 WT | ns |
| day 13 WT vs day 15 WT | ns |
| day 15 WT vs day 18 WT | ns |
| day 18 WT vs day 20 WT | ns |
| day 4 *age-1(hx546)* vs day 6 *age-1(hx546)* | ns |
| day 6 *age-1(hx546)* vs day 8 *age-1(hx546)* | ns |
| day 8 *age-1(hx546)* vs day 11 *age-1(hx546)* | ns |
| day 11 *age-1(hx546)* vs day 13 *age-1(hx546)* | ns |
| day 13 *age-1(hx546)* vs day 15 *age-1(hx546)* | ns |
| day 15 *age-1(hx546)* vs day 18 *age-1(hx546)* | ns |
| day 18 *age-1(hx546)* vs day 20 *age-1(hx546)* | ns |
| day 4 *daf-16(mgDf50)* vs day 6 *daf-16(mgDf50)* | ns |
| day 6 *daf-16(mgDf50)* vs day 8 *daf-16(mgDf50)* | ns |
| day 8 *daf-16(mgDf50)* vs day 11 *daf-16(mgDf50)* | ns |
| day 11 *daf-16(mgDf50)* vs day 13 *daf-16(mgDf50)* | ns |
| day 13 *daf-16(mgDf50)* vs day 15 *daf-16(mgDf50)* | * |
| day 15 *daf-16(mgDf50)* vs day 18 *daf-16(mgDf50)* | ns |
| day 18 *daf-16(mgDf50)* vs day 20 *daf-16(mgDf50)* | ns |

**D Stretch**

- One-way ANOVA, followed by Dunnett’s multiple comparison test

| **Stretch** | |  |
| --- | --- | --- |
| day 4 | WT vs *age-1(hx546)* | * |
|  | WT vs *daf-16(mgDf50)* | ns |
| day 6 | WT vs *age-1(hx546)* | *** |
|  | WT vs *daf-16(mgDf50)* | ns |
| day 8 | WT vs *age-1(hx546)* | *** |
|  | WT vs *daf-16(mgDf50)* | ** |
| day 11 | WT vs *age-1(hx546)* | * |
|  | WT *vs daf-16(mgDf50)* | ns |
| day 13 | WT vs *age-1(hx546)* | ns |
|  | WT vs *daf-16(mgDf50)* | * |
| day 15 | WT vs *age-1(hx546)* | ns |
|  | WT vs *daf-16(mgDf50)* | * |
| day 18 | WT vs *age-1(hx546)* | ns |
|  | WT vs *daf-16(mgDf50)* | ns |
| day 20 | WT vs *age-1(hx546)* | ns |
|  | WT vs *daf-16(mgDf50)* | *** |

- One-way ANOVA, followed by Bonferroni’s multiple comparison test

| **Stretch** |  |
| --- | --- |
| day 4 WT vs day 6 WT | ns |
| day 6 WT vs day 8 WT | ns |
| day 8 WT vs day 11 WT | ns |
| day 11 WT vs day 13 WT | ns |
| day 13 WT vs day 15 WT | ns |
| day 15 WT vs day 18 WT | * |
| day 18 WT vs day 20 WT | **** |
| day 4 *age-1(hx546)* vs day 6 *age-1(hx546)* | ns |
| day 6 *age-1(hx546)* vs day 8 *age-1(hx546)* | ns |
| day 8 *age-1(hx546)* vs day 11 *age-1(hx546)* | ns |
| day 11 *age-1(hx546)* vs day 13 *age-1(hx546)* | *** |
| day 13 *age-1(hx546)* vs day 15 *age-1(hx546)* | ns |
| day 15 *age-1(hx546)* vs day 18 *age-1(hx546)* | **** |
| day 18 *age-1(hx546)* vs day 20 *age-1(hx546)* | * |
| day 4 *daf-16(mgDf50)* vs day 6 *daf-16(mgDf50)* | ns |
| day 6 *daf-16(mgDf50)* vs day 8 *daf-16(mgDf50)* | ns |
| day 8 *daf-16(mgDf50)* vs day 11 *daf-16(mgDf50)* | ns |
| day 11 *daf-16(mgDf50)* vs day 13 *daf-16(mgDf50)* | ns |
| day 13 *daf-16(mgDf50)* vs day 15 *daf-16(mgDf50)* | ns |
| day 15 *daf-16(mgDf50)* vs day 18 *daf-16(mgDf50)* | ** |
| day 18 *daf-16(mgDf50)* vs day 20 *daf-16(mgDf50)* | ns |

**E Attenuation**

- One-way ANOVA, followed by Dunnett’s multiple comparison test

| **Attenuation** | |  |
| --- | --- | --- |
| day 4 | WT vs *age-1(hx546)* | ns |
|  | WT vs *daf-16(mgDf50)* | *** |
| day 6 | WT vs *age-1(hx546)* | *** |
|  | WT vs *daf-16(mgDf50)* | *** |
| day 8 | WT vs *age-1(hx546)* | *** |
|  | WT vs *daf-16(mgDf50)* | *** |
| day 11 | WT vs *age-1(hx546)* | ** |
|  | WT vs *daf-16(mgDf50)* | ns |
| day 13 | WT vs *age-1(hx546)* | ** |
|  | WT vs *daf-16(mgDf50)* | * |
| day 15 | WT vs *age-1(hx546)* | ns |
|  | WT vs *daf-16(mgDf50)* | ns |
| day 18 | WT vs *age-1(hx546)* | ns |
|  | WT vs *daf-16(mgDf50)* | *** |
| day 20 | WT vs *age-1(hx546)* | ns |
|  | WT vs *daf-16(mgDf50)* | ** |

- One-way ANOVA, followed by Bonferroni’s multiple comparison test

| **Attenuation** |  |
| --- | --- |
| day 4 WT vs day 6 WT | ns |
| day 6 WT vs day 8 WT | ns |
| day 8 WT vs day 11 WT | ns |
| day 11 WT vs day 13 WT | ns |
| day 13 WT vs day 15 WT | **** |
| day 15 WT vs day 18 WT | ** |
| day 18 WT vs day 20 WT | ns |
| day 4 *age-1(hx546)* vs day 6 *age-1(hx546)* | ns |
| day 6 *age-1(hx546)* vs day 8 *age-1(hx546)* | **** |
| day 8 *age-1(hx546)* vs day 11 *age-1(hx546)* | ns |
| day 11 *age-1(hx546)* vs day 13 *age-1(hx546)* | ns |
| day 13 *age-1(hx546)* vs day 15 *age-1(hx546)* | ns |
| day 15 *age-1(hx546)* vs day 18 *age-1(hx546)* | ns |
| day 18 *age-1(hx546)* vs day 20 *age-1(hx546)* | *** |
| day 4 *daf-16(mgDf50)* vs day 6 *daf-16(mgDf50)* | ns |
| day 6 *daf-16(mgDf50)* vs day 8 *daf-16(mgDf50)* | ns |
| day 8 *daf-16(mgDf50)* vs day 11 *daf-16(mgDf50)* | ns |
| day 11 *daf-16(mgDf50)* vs day 13 *daf-16(mgDf50)* | ns |
| day 13 *daf-16(mgDf50)* vs day 15 *daf-16(mgDf50)* | **** |
| day 15 *daf-16(mgDf50)* vs day 18 *daf-16(mgDf50)* | ns |
| day 18 *daf-16(mgDf50)* vs day 20 *daf-16(mgDf50)* | ns |

**F Reverse swimming**

- One-way ANOVA, followed by Dunnett’s multiple comparison test

| **Reverse swimming** | |  |
| --- | --- | --- |
| day 4 | WT vs *age-1(hx546)* | ns |
|  | WT vs *daf-16(mgDf50)* | ns |
| day 6 | WT vs *age-1(hx546)* | ns |
|  | WT vs *daf-16(mgDf50)* | ns |
| day 8 | WT vs *age-1(hx546)* | ** |
|  | WT vs *daf-16(mgDf50)* | ns |
| day 11 | WT vs *age-1(hx546)* | ns |
|  | WT vs *daf-16(mgDf50)* | ns |
| day 13 | WT vs *age-1(hx546)* | ns |
|  | WT vs *daf-16(mgDf50)* | ns |
| day 15 | WT vs *age-1(hx546)* | ns |
|  | WT vs *daf-16(mgDf50)* | ns |
| day 18 | WT vs *age-1(hx546)* | ns |
|  | WT vs *daf-16(mgDf50)* | ns |
| day 20 | WT vs age-1(hx546) | ns |
|  | WT vs daf-16(mgDf50) | ns |

- One-way ANOVA, followed by Bonferroni’s multiple comparison test

| **Reverse swimming** |  |
| --- | --- |
| day 4 WT vs day 6 WT | ns |
| day 6 WT vs day 8 WT | ns |
| day 8 WT vs day 11 WT | ns |
| day 11 WT vs day 13 WT | ns |
| day 13 WT vs day 15 WT | ns |
| day 15 WT vs day 18 WT | ns |
| day 18 WT vs day 20 WT | ns |
| day 4 *age-1(hx546)* vs day 6 *age-1(hx546)* | ns |
| day 6 *age-1(hx546)* vs day 8 *age-1(hx546)* | ns |
| day 8 *age-1(hx546)* vs day 11 *age-1(hx546)* | ns |
| day 11 *age-1(hx546)* vs day 13 *age-1(hx546)* | ns |
| day 13 *age-1(hx546)* vs day 15 *age-1(hx546)* | ns |
| day 15 *age-1(hx546)* vs day 18 *age-1(hx546)* | ns |
| day 18 *age-1(hx546)* vs day 20 *age-1(hx546)* | ns |
| day 4 *daf-16(mgDf50)* vs day 6 *daf-16(mgDf50)* | ns |
| day 6 *daf-16(mgDf50)* vs day 8 *daf-16(mgDf50)* | ns |
| day 8 *daf-16(mgDf50)* vs day 11 *daf-16(mgDf50)* | ns |
| day 11 *daf-16(mgDf50)* vs day 13 *daf-16(mgDf50)* | ns |
| day 13 *daf-16(mgDf50)* vs day 15 *daf-16(mgDf50)* | ns |
| day 15 *daf-16(mgDf50)* vs day 18 *daf-16(mgDf50)* | ns |
| day 18 *daf-16(mgDf50)* vs day 20 *daf-16(mgDf50)* | ns |

**G Curling**

- One-way ANOVA, followed by Dunnett’s multiple comparison test

| **Curling** | |  |
| --- | --- | --- |
| day 4 | WT vs *age-1(hx546)* | ns |
|  | WT vs *daf-16(mgDf50)* | ns |
| day 6 | WT vs *age-1(hx546)* | ns |
|  | WT vs *daf-16(mgDf50)* | ns |
| day 8 | WT vs *age-1(hx546)* | ns |
|  | WT vs *daf-16(mgDf50)* | ns |
| day 11 | WT vs *age-1(hx546)* | ns |
|  | WT vs *daf-16(mgDf50)* | ns |
| day 13 | WT vs *age-1(hx546)* | ns |
|  | WT vs *daf-16(mgDf50)* | ns |
| day 15 | WT vs *age-1(hx546)* | ns |
|  | WT vs *daf-16(mgDf50)* | ns |
| day 18 | WT vs *age-1(hx546)* | ** |
|  | WT vs *daf-16(mgDf50)* | ns |
| day 20 | WT vs *age-1(hx546)* | ns |
|  | WT vs *daf-16(mgDf50)* | ** |

- One-way ANOVA, followed by Bonferroni’s multiple comparison test

| **Curling** |  |
| --- | --- |
| day 4 WT vs day 6 WT | ns |
| day 6 WT vs day 8 WT | ns |
| day 8 WT vs day 11 WT | ns |
| day 11 WT vs day 13 WT | ns |
| day 13 WT vs day 15 WT | ns |
| day 15 WT vs day 18 WT | ns |
| day 18 WT vs day 20 WT | *** |
| day 4 *age-1(hx546)* vs day 6 *age-1(hx546)* | ns |
| day 6 *age-1(hx546)* vs day 8 *age-1(hx546)* | ns |
| day 8 *age-1(hx546)* vs day 11 *age-1(hx546)* | ns |
| day 11 *age-1(hx546)* vs day 13 *age-1(hx546)* | ns |
| day 13 *age-1(hx546)* vs day 15 *age-1(hx546)* | ns |
| day 15 *age-1(hx546)* vs day 18 *age-1(hx546)* | * |
| day 18 *age-1(hx546)* vs day 20 *age-1(hx546)* | ns |
| day 4 *daf-16(mgDf50)* vs day 6 *daf-16(mgDf50)* | ns |
| day 6 *daf-16(mgDf50)* vs day 8 *daf-16(mgDf50)* | ns |
| day 8 *daf-16(mgDf50)* vs day 11 *daf-16(mgDf50)* | ns |
| day 11 *daf-16(mgDf50)* vs day 13 *daf-16(mgDf50)* | ns |
| day 13 *daf-16(mgDf50)* vs day 15 *daf-16(mgDf50)* | ns |
| day 15 *daf-16(mgDf50)* vs day 18 *daf-16(mgDf50)* | ns |
| day 18 *daf-16(mgDf50)* vs day 20 *daf-16(mgDf50)* | ns |

**H Travel speed**

- One-way ANOVA, followed by Dunnett’s multiple comparison test

| **Travel speed** | |  |
| --- | --- | --- |
| day 4 | WT vs *age-1(hx546)* | *** |
|  | WT vs *daf-16(mgDf50)* | ** |
| day 6 | WT vs *age-1(hx546)* | *** |
|  | WT vs *daf-16(mgDf50)* | ns |
| day 8 | WT vs *age-1(hx546)* | *** |
|  | WT vs *daf-16(mgDf50)* | ns |
| day 11 | WT vs *age-1(hx546)* | *** |
|  | WT vs *daf-16(mgDf50)* | ns |
| day 13 | WT vs *age-1(hx546)* | ns |
|  | WT vs *daf-16(mgDf50)* | *** |
| day 15 | WT vs *age-1(hx546)* | * |
|  | WT vs *daf-16(mgDf50)* | *** |
| day 18 | WT vs *age-1(hx546)* | ns |
|  | WT vs *daf-16(mgDf50)* | * |
| day 20 | WT vs *age-1(hx546)* | ns |
|  | WT vs *daf-16(mgDf50)* | *** |

- One-way ANOVA, followed by Bonferroni’s multiple comparison test

| **Travel speed** |  |
| --- | --- |
| day 4 WT vs day 6 WT | ns |
| day 6 WT vs day 8 WT | ns |
| day 8 WT vs day 11 WT | *** |
| day 11 WT vs day 13 WT | ** |
| day 13 WT vs day 15 WT | ns |
| day 15 WT vs day 18 WT | **** |
| day 18 WT vs day 20 WT | ns |
| day 4 *age-1(hx546)* vs day 6 *age-1(hx546)* | ns |
| day 6 *age-1(hx546)* vs day 8 *age-1(hx546)* | ns |
| day 8 *age-1(hx546)* vs day 11 *age-1(hx546)* | * |
| day 11 *age-1(hx546)* vs day 13 *age-1(hx546)* | ns |
| day 13 *age-1(hx546)* vs day 15 *age-1(hx546)* | ns |
| day 15 *age-1(hx546)* vs day 18 *age-1(hx546)* | **** |
| day 18 *age-1(hx546)* vs day 20 *age-1(hx546)* | ns |
| day 4 *daf-16(mgDf50)* vs day 6 *daf-16(mgDf50)* | ns |
| day 6 *daf-16(mgDf50)* vs day 8 *daf-16(mgDf50)* | ns |
| day 8 *daf-16(mgDf50)* vs day 11 *daf-16(mgDf50)* | *** |
| day 11 *daf-16(mgDf50)* vs day 13 *daf-16(mgDf50)* | ns |
| day 13 *daf-16(mgDf50)* vs day 15 *daf-16(mgDf50)* | *** |
| day 15 *daf-16(mgDf50)* vs day 18 *daf-16(mgDf50)* | * |
| day 18 *daf-16(mgDf50)* vs day 20 *daf-16(mgDf50)* | ns |

**I Brush stroke**

- One-way ANOVA, followed by Dunnett’s multiple comparison test

| **Brush stroke** | |  |
| --- | --- | --- |
| day 4 | WT vs *age-1(hx546)* | *** |
|  | WT vs *daf-16(mgDf50)* | ns |
| day 6 | WT vs *age-1(hx546)* | *** |
|  | WT vs *daf-16(mgDf50)* | ns |
| day 8 | WT vs *age-1(hx546)* | *** |
|  | WT vs *daf-16(mgDf50)* | ns |
| day 11 | WT vs *age-1(hx546)* | *** |
|  | WT vs *daf-16(mgDf50)* | ns |
| day 13 | WT vs *age-1(hx546)* | ns |
|  | WT vs *daf-16(mgDf50)* | *** |
| day 15 | WT vs *age-1(hx546)* | ns |
|  | WT vs *daf-16(mgDf50)* | *** |
| day 18 | WT vs *age-1(hx546)* | ns |
|  | WT vs *daf-16(mgDf50)* | ns |
| day 20 | WT vs *age-1(hx546)* | ns |
|  | WT vs *daf-16(mgDf50)* | *** |

- One-way ANOVA, followed by Bonferroni’s multiple comparison test

| **Brush stroke** |  |
| --- | --- |
| day 4 WT vs day 6 WT | ns |
| day 6 WT vs day 8 WT | ns |
| day 8 WT vs day 11 WT | ** |
| day 11 WT vs day 13 WT | ** |
| day 13 WT vs day 15 WT | ns |
| day 15 WT vs day 18 WT | **** |
| day 18 WT vs day 20 WT | ns |
| day 4 *age-1(hx546)* vs day 6 *age-1(hx546)* | ns |
| day 6 *age-1(hx546)* vs day 8 *age-1(hx546)* | ns |
| day 8 *age-1(hx546)* vs day 11 *age-1(hx546)* | * |
| day 11 *age-1(hx546)* vs day 13 *age-1(hx546)* | ns |
| day 13 *age-1(hx546)* vs day 15 *age-1(hx546)* | ns |
| day 15 *age-1(hx546)* vs day 18 *age-1(hx546)* | **** |
| day 18 *age-1(hx546)* vs day 20 *age-1(hx546)* | ns |
| day 4 *daf-16(mgDf50)* vs day 6 *daf-16(mgDf50)* | ns |
| day 6 *daf-16(mgDf50)* vs day 8 *daf-16(mgDf50)* | ns |
| day 8 *daf-16(mgDf50)* vs day 11 *daf-16(mgDf50)* | *** |
| day 11 *daf-16(mgDf50)* vs day 13 *daf-16(mgDf50)* | ns |
| day 13 *daf-16(mgDf50)* vs day 15 *daf-16(mgDf50)* | ** |
| day 15 *daf-16(mgDf50)* vs day 18 *daf-16(mgDf50)* | ns |
| day 18 *daf-16(mgDf50)* vs day 20 *daf-16(mgDf50)* | ns |

**J Activity index**

- One-way ANOVA, followed by Dunnett’s multiple comparison test

| **Activity index** | |  |
| --- | --- | --- |
| day 4 | WT vs *age-1(hx546)* | *** |
|  | WT vs *daf-16(mgDf50)* | ns |
| day 6 | WT vs *age-1(hx546)* | *** |
|  | WT vs *daf-16(mgDf50)* | ns |
| day 8 | WT vs *age-1(hx546)* | *** |
|  | WT vs *daf-16(mgDf50)* | ns |
| day 11 | WT vs *age-1(hx546)* | *** |
|  | WT vs *daf-16(mgDf50)* | ns |
| day 13 | WT vs *age-1(hx546)* | ns |
|  | WT vs *daf-16(mgDf50)* | *** |
| day 15 | WT vs *age-1(hx546)* | ns |
|  | WT vs *daf-16(mgDf50)* | *** |
| day 18 | WT vs *age-1(hx546)* | ns |
|  | WT vs *daf-16(mgDf50)* | * |
| day 20 | WT vs *age-1(hx546)* | ns |
|  | WT vs *daf-16(mgDf50)* | *** |

- One-way ANOVA, followed by Bonferroni’s multiple comparison test

| **Activity index** |  |
| --- | --- |
| day 4 WT vs day 6 WT | ns |
| day 6 WT vs day 8 WT | ns |
| day 8 WT vs day 11 WT | *** |
| day 11 WT vs day 13 WT | ** |
| day 13 WT vs day 15 WT | ns |
| day 15 WT vs day 18 WT | *** |
| day 18 WT vs day 20 WT | * |
| day 4 *age-1(hx546)* vs day 6 *age-1(hx546)* | ns |
| day 6 *age-1(hx546)* vs day 8 *age-1(hx546)* | ns |
| day 8 *age-1(hx546)* vs day 11 *age-1(hx546)* | * |
| day 11 *age-1(hx546)* vs day 13 *age-1(hx546)* | ns |
| day 13 *age-1(hx546)* vs day 15 *age-1(hx546)* | ns |
| day 15 *age-1(hx546)* vs day 18 *age-1(hx546)* | *** |
| day 18 *age-1(hx546)* vs day 20 *age-1(hx546)* | ns |
| day 4 *daf-16(mgDf50)* vs day 6 *daf-16(mgDf50)* | ns |
| day 6 *daf-16(mgDf50)* vs day 8 *daf-16(mgDf50)* | ns |
| day 8 *daf-16(mgDf50)* vs day 11 *daf-16(mgDf50)* | *** |
| day 11 *daf-16(mgDf50)* vs day 13 *daf-16(mgDf50)* | ns |
| day 13 *daf-16(mgDf50)* vs day 15 *daf-16(mgDf50)* | *** |
| day 15 *daf-16(mgDf50)* vs day 18 *daf-16(mgDf50)* | * |
| day 18 *daf-16(mgDf50)* vs day 20 *daf-16(mgDf50)* | ns |
