# Supplementary material for: CeleST: Computer Vision Software for Quantitative Analysis of C. elegans Swim Behavior Reveals Novel Features of Locomotion
Source: PLoS Comput Biol. 2014 Jul 17;10(7):e1003702. doi: 10.1371/journal.pcbi.1003702 (PMC4102393; doi:10.1371/journal.pcbi.1003702)
Supplement: Table S1 — Comparison of manual and CeleST scores. A, Average of manually scored head bends is analogous to CeleST Wave initiation rate. Sample size () is 108 day 4 adults for manual score of head bends and 101 for automated analysis with CeleST. B, CeleST is more sensitive scoring Reverse swimming and Curling, especially for brief events. for manual score of Reverse swimming, for manual score of Curling, and for CeleST automated analysis of Reverse swimming and Curling. All manual scores were executed on a computer screen using recorded videos of the same animals recorded by CeleST. * CeleST automatically measures 10 parameters in a single analysis of one video in about 5 min. The 13.3 min is an estimate of the time CeleST takes to analyze one parameter for 101 animals in 27 videos. (DOCX) [file pcbi.1003702.s007.docx]

**Table S1. Comparison of manual and CeleST scores.**  **A**, Average of manually scored head bends is analogous to CeleST Wave initiation rate. Sample size (n) is 108 day 4 adults for manual score of head bends and 101 for automated analysis with CeleST. **B**, CeleST is more sensitive scoring Reverse swimming and Curling, especially for brief events. n=108 for manual score of Reverse swimming, n=36 for manual score of Curling, and n=101 for CeleST automated analysis of Reverse swimming and Curling. All manual scores were executed on a computer screen using recorded videos of the same animals recorded by CeleST. ***** CeleST automatically measures 10 parameters in a single analysis of one video in about 5min. The 13.3 min is an estimate of the time CeleST takes to analyze one parameter for 101 animals in 27 videos.

**A**

|  | **Wave initiation rate** | |
| --- | --- | --- |
| **Procedure** | **Manual** | **CeleST** |
| **Average** | 75.05 | 76.06 |
| **Total n** | 108 | 101 |
| **Time Spent for Analysis** | 108 min | 13.3 min* |

**B**

|  | **Reverse Swimming** | | **Curling** | |
| --- | --- | --- | --- | --- |
| **Procedure** | **Manual** | **CeleST** | **Manual** | **CeleST** |
| **Percentage of animals Reverse Swimming or Curling** | 3.7 | 20.8 | 30.5 | 43.6 |
| **Total n** | 108 | 101 | 36 | 101 |
| **Range of Percentage of Time Spent Reverse Swimming or Curling in a Swim trial** | 0.92 – 11.21 | 0.18 – 25.39 | 0.37 – 3.68 | 0.36 – 18.98 |
| **Time Spent for Analysis** | 100 min | 13.3 min* | 295 min | 13.3 min* |
