# Supplementary material for: CeleST: Computer Vision Software for Quantitative Analysis of C. elegans Swim Behavior Reveals Novel Features of Locomotion
Source: PLoS Comput Biol. 2014 Jul 17;10(7):e1003702. doi: 10.1371/journal.pcbi.1003702 (PMC4102393; doi:10.1371/journal.pcbi.1003702)
Supplement: Text S1 — Further details on each of the ten parameter measurements extracted with CeleST (panels C–O of Figure 2 ). (DOCX) [file pcbi.1003702.s009.docx]

**Text S1. Further details on each of the ten parameter measurements extracted with CeleST (panels C-O of Figure 2).**

**C**, Wave initiation rate. The number of waves initiated from either the head (most commonly) or the tail (reverse swimming) per unit time are measured as described in the text. Wave initiation rate corresponds approximately to the number of stripes with respect to time in **B**, and is analogous to manually-counted body-bend rate.

**D**, Body wave number. The number of waves in transit through the body at a point in time is scored, as an indicator of the average “waviness” of the body posture. A negative score reflects a wave in reverse, from tail to head.

**E**, Asymmetry. This measure reports on whether the animal bends more toward one side (clockwise, - score) or the other (counterclockwise, + score) in a swim trial. A balanced swimmer would have a score of 0; an asymmetric swimmer would have a positive or negative value that we have empirically observed may reach +/- 0.2 /mm.

**F**, Stretch. The maximum difference in curvature that occurs between the two most extreme curvature scores for any part an animal during a given stroke is recorded over time. A high stretch score indicates that body bends are deep (high stretch), whereas a low score indicates a flat posture. Unlike Attenuation (see below), which compares specifically the head and tail, the Stretch score compares the most extreme curvature differences that occur between any two body points. Note that an animal might still be flexible, but simply not stretch much in a given swim. We have observed that an animal with a high wave initiation rate often appears to swim so fast that a deep body bend does not occur and thus that animal will have a low stretch score.

**G**, Attenuation. How precisely the depth of a wave is maintained as it propagates down the body is measured to address whether the initial amplitude of the head is similar to the amplitude when the wave reaches the tail. For the Attenuation measure we average plotted values for individual records over a sliding frame of 2 strokes to calculate a score that corresponds to 1 - tail/head curvature ratio, and display it as a percentage. 0% attenuation is no change in wave depth from head to tail; 100% attenuation would correspond to an animal that had no tail movement at all even though the head was active. Negative attenuation happens when the tail has a deeper wave than the head.

**H**, Reverse swimming. The Body wave number (from **D**) is plotted as a function of time as the black line; this value is negative when the animal swims in reverse, with tail appearing to drive the wave through the body, as highlighted in red. The final score is the total time spent in reverse swimming during the video as a percentage of the total swimming time. Dynamic examples of Reverse swimming can be viewed in Supplementary Videos on http://celest.mbb.rutgers.edu.

**I**, Curling. The distance between either extremity and the opposite side of the body is plotted as a function of time in black; when this distance reaches 0, the animal is scored as curled and is noted on the plot in red. Note that the curling measure can detect the “O” shape when head and tail overlap, as well as “6” shape in which part of the body stays rigid and part curls back to itself. The final score is the total percentage of time spent curling. Note that this measure is computed on coordinates rather than curvature measures.

**J**, Trajectory of one animal during the video, from t=0s to t=30s. The position center of gravity of the worm computed over two strokes is tracked and plotted. This removes the lateral motions of the animal and focuses on the actual traveling around the swimming zone. Note that for illustration purposes, this is a distinct worm from that used in **A-I**.

**K**, Positions of the worm body during the video, superimposed over the same image as **J**. For clarity, only one in five body screenshots are displayed.

**L**, Zoom-in of green-boxed area in **K** in which all consecutive body screenshots are displayed.

**M**, Travel speed. The trajectory in **J** is used to compute the travel speed for that trial as the total distance traveled over the duration of the video.

**N**, Brush stroke. The area that the body of the animal would cover or “paint” in a complete stroke, normalized to body size, is scored. We subtract the number of pixels covered by the body at rest from the total score so that animals at rest receives a zero score, and the paint score is (number of pixels painted in total/number of pixels of the body) – 1 body pixel covering. A high score reflects a large local coverage, whereas a low score indicates modest deflection as the animal swims. The brush stroke measure thus gives an indication of the depth of the movement the animal has accomplished in a given stroke.

**O**, Activity index. This parameter sums the pixels that are painted by the body during the time that it takes an animal to do two strokes to provide a sense of how vigorously the animal bends while swimming over time. Similar to the individual brush stroke measurement, we normalize pixels/body size and we subtract one body area from the total. Notice how by the middle of the video in **J-O**, the worm pauses its traveling (plateau in **M**) and reduces its activity (drop in **O**), but its brush stroke is relatively unaffected (**N**).
