# Supplementary material for: CeleST: Computer Vision Software for Quantitative Analysis of C. elegans Swim Behavior Reveals Novel Features of Locomotion
Source: PLoS Comput Biol. 2014 Jul 17;10(7):e1003702. doi: 10.1371/journal.pcbi.1003702 (PMC4102393; doi:10.1371/journal.pcbi.1003702)
Supplement: Text S2 — C. elegans strains, growth and swim analysis methods. (DOCX) [file pcbi.1003702.s010.docx]

**Text S2. *C. elegans* strains, growth and swim analysis.**

*C. elegans* were grown on solid media with OP50 bacterial food according to standard culture procedures (Brenner, 1974) . All experiments were performed at 20^o^C. Strains used were N2 Bristol, which is the canonical wild-type strain, and the behavioral mutant OR930 [*glr-1(ky176)* *III*].

Locomotion in liquid (swimming) of *C. elegans* was measured at ages day 4 (young, reproductive adult) and day 11 (post-reproductive adult). Adult nematodes were transferred free of bacteria from solid media to 60 µl of liquid (1xM9 buffer) in groups of four or five. The 60µl drop of M9 buffer was contained in a 1cm circle imprinted on a microscope slide. Care was taken to spread out the group of adults in the encircled liquid drop to avoid overlapping, which can complicate subsequent analysis of locomotion. Adults were visualized with a Stemi 2000-C stereomicroscope (Carl Zeiss), and their swimming was recorded over 30 seconds with the aid of a CCD camera (Rolera-XR Mono Fast 1394, Qimaging) and the software Streamprix 3.17.2 (Norpix, 2002).

We used the following low-end requirements: image size: 696 x 520 pixel; image resolution: 0.02 mm / pixel (1 mm ~ 50 pixel); frame rate: 18 frames / sec. Any improvement on these values will make the tracking easier, but would not affect the measures calculated by the program. Our videos are 30s long, although this is a setup choice and not a technical limitation. Longer videos can be handled in the same way, and are only limited by computer memory. The time to fully process (from upload to display results) a 30 second/544 frames video is about 5 minutes using an iMac mid-2007 2.8GHz Core 2 Duo with 4Gb RAM. It is expected that the same code running on more recent, faster processors with more RAM would reduce this speed. We have also noted (but not measured) that when available, pre-compiled MATLAB code runs faster.

A limitation of a top-down filming system is that an animal swimming upwards towards the camera would appear as a small dot, partly occluding itself and preventing its valid tracking and measuring. Our slide setup diminishes the possibility of animals swimming upwards, and we note that as a general imaging issue, more than one camera would be necessary to record and analyze 3D motions of swimming nematodes.
