# Supplementary material for: CeleST: Computer Vision Software for Quantitative Analysis of C. elegans Swim Behavior Reveals Novel Features of Locomotion
Source: PLoS Comput Biol. 2014 Jul 17;10(7):e1003702. doi: 10.1371/journal.pcbi.1003702 (PMC4102393; doi:10.1371/journal.pcbi.1003702)
Supplement: Dataset S1 — Zip package containing source code and demos of the software CeleST. (ZIP) [file pcbi.1003702.s008.zip › CeleST submission small/demos/CeleST - demo with measures/User guide - measures.pdf]

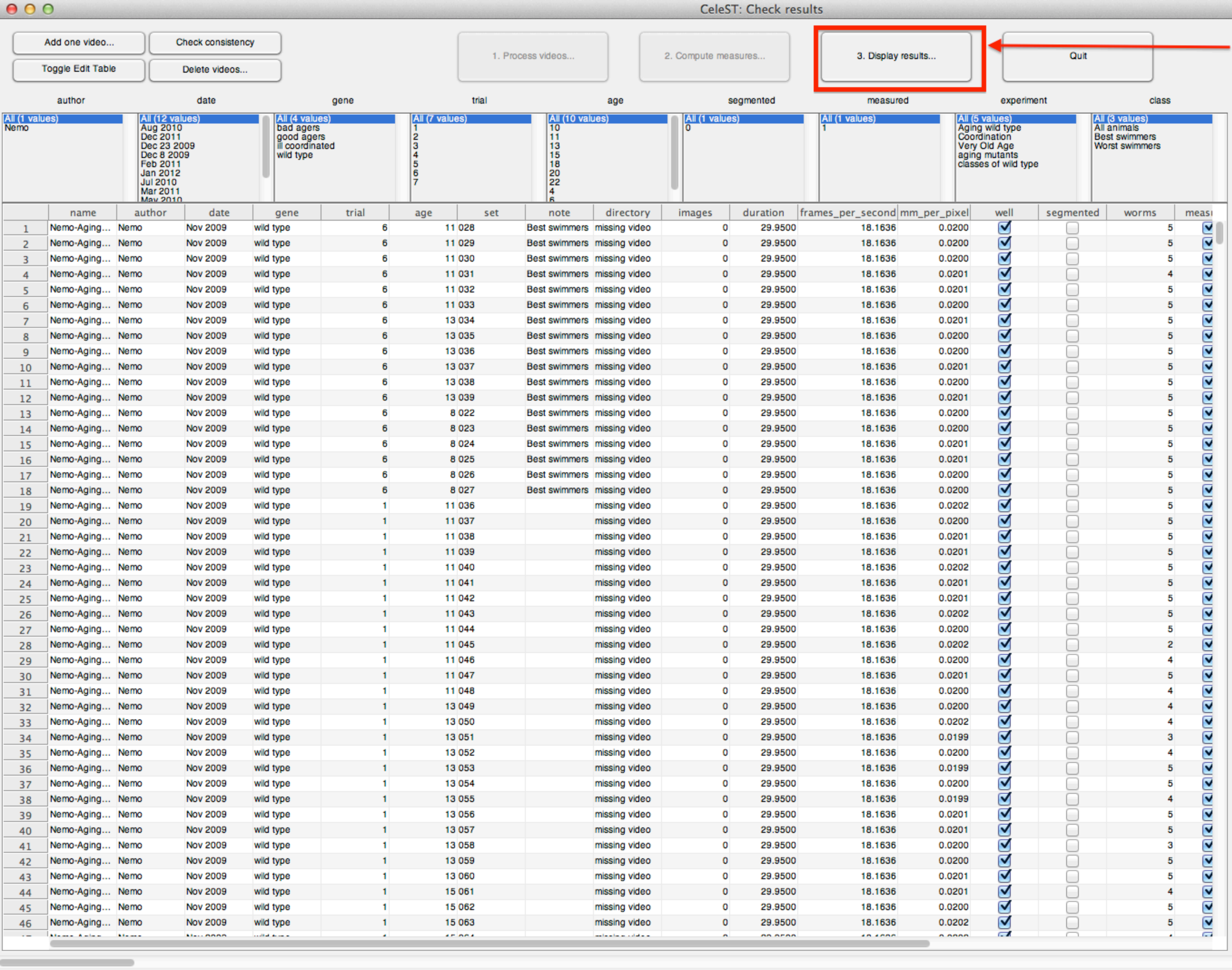


| | name | author | date | gene | trial | age | set | note | directory | images | duration | frames_per_second | mm_per_pixel | well | segmented | worms | meas |
|---|---|---|---|---|---|---|---|---|---|---|---|---|---|---|---|---|---|
| 1 | Nemo-Aging... | Nemo | Nov 2009 | wild type | 6 | 11 | 028 | Best swimmers | missing video | 0 | 29.9500 | 18.1636 | 0.0200 | ☑ | ☐ | 5 | ☑ |
| 2 | Nemo-Aging... | Nemo | Nov 2009 | wild type | 6 | 11 | 029 | Best swimmers | missing video | 0 | 29.9500 | 18.1636 | 0.0200 | ☑ | ☐ | 5 | ☑ |
| 3 | Nemo-Aging... | Nemo | Nov 2009 | wild type | 6 | 11 | 030 | Best swimmers | missing video | 0 | 29.9500 | 18.1636 | 0.0200 | ☑ | ☐ | 5 | ☑ |
| 4 | Nemo-Aging... | Nemo | Nov 2009 | wild type | 6 | 11 | 031 | Best swimmers | missing video | 0 | 29.9500 | 18.1636 | 0.0201 | ☑ | ☐ | 4 | ☑ |
| 5 | Nemo-Aging... | Nemo | Nov 2009 | wild type | 6 | 11 | 032 | Best swimmers | missing video | 0 | 29.9500 | 18.1636 | 0.0201 | ☑ | ☐ | 5 | ☑ |
| 6 | Nemo-Aging... | Nemo | Nov 2009 | wild type | 6 | 11 | 033 | Best swimmers | missing video | 0 | 29.9500 | 18.1636 | 0.0200 | ☑ | ☐ | 5 | ☑ |
| 7 | Nemo-Aging... | Nemo | Nov 2009 | wild type | 6 | 13 | 034 | Best swimmers | missing video | 0 | 29.9500 | 18.1636 | 0.0201 | ☑ | ☐ | 5 | ☑ |
| 8 | Nemo-Aging... | Nemo | Nov 2009 | wild type | 6 | 13 | 035 | Best swimmers | missing video | 0 | 29.9500 | 18.1636 | 0.0200 | ☑ | ☐ | 5 | ☑ |
| 9 | Nemo-Aging... | Nemo | Nov 2009 | wild type | 6 | 13 | 036 | Best swimmers | missing video | 0 | 29.9500 | 18.1636 | 0.0200 | ☑ | ☐ | 5 | ☑ |
| 10 | Nemo-Aging... | Nemo | Nov 2009 | wild type | 6 | 13 | 037 | Best swimmers | missing video | 0 | 29.9500 | 18.1636 | 0.0201 | ☑ | ☐ | 5 | ☑ |
| 11 | Nemo-Aging... | Nemo | Nov 2009 | wild type | 6 | 13 | 038 | Best swimmers | missing video | 0 | 29.9500 | 18.1636 | 0.0200 | ☑ | ☐ | 5 | ☑ |
| 12 | Nemo-Aging... | Nemo | Nov 2009 | wild type | 6 | 13 | 039 | Best swimmers | missing video | 0 | 29.9500 | 18.1636 | 0.0201 | ☑ | ☐ | 5 | ☑ |
| 13 | Nemo-Aging... | Nemo | Nov 2009 | wild type | 6 | 8 | 022 | Best swimmers | missing video | 0 | 29.9500 | 18.1636 | 0.0200 | ☑ | ☐ | 5 | ☑ |
| 14 | Nemo-Aging... | Nemo | Nov 2009 | wild type | 6 | 8 | 023 | Best swimmers | missing video | 0 | 29.9500 | 18.1636 | 0.0200 | ☑ | ☐ | 5 | ☑ |
| 15 | Nemo-Aging... | Nemo | Nov 2009 | wild type | 6 | 8 | 024 | Best swimmers | missing video | 0 | 29.9500 | 18.1636 | 0.0201 | ☑ | ☐ | 5 | ☑ |
| 16 | Nemo-Aging... | Nemo | Nov 2009 | wild type | 6 | 8 | 025 | Best swimmers | missing video | 0 | 29.9500 | 18.1636 | 0.0201 | ☑ | ☐ | 5 | ☑ |
| 17 | Nemo-Aging... | Nemo | Nov 2009 | wild type | 6 | 8 | 026 | Best swimmers | missing video | 0 | 29.9500 | 18.1636 | 0.0200 | ☑ | ☐ | 5 | ☑ |
| 18 | Nemo-Aging... | Nemo | Nov 2009 | wild type | 6 | 8 | 027 | Best swimmers | missing video | 0 | 29.9500 | 18.1636 | 0.0200 | ☑ | ☐ | 5 | ☑ |
| 19 | Nemo-Aging... | Nemo | Nov 2009 | wild type | 1 | 11 | 036 | | missing video | 0 | 29.9500 | 18.1636 | 0.0202 | ☑ | ☐ | 5 | ☑ |
| 20 | Nemo-Aging... | Nemo | Nov 2009 | wild type | 1 | 11 | 037 | | missing video | 0 | 29.9500 | 18.1636 | 0.0200 | ☑ | ☐ | 5 | ☑ |
| 21 | Nemo-Aging... | Nemo | Nov 2009 | wild type | 1 | 11 | 038 | | missing video | 0 | 29.9500 | 18.1636 | 0.0201 | ☑ | ☐ | 5 | ☑ |
| 22 | Nemo-Aging... | Nemo | Nov 2009 | wild type | 1 | 11 | 039 | | missing video | 0 | 29.9500 | 18.1636 | 0.0201 | ☑ | ☐ | 5 | ☑ |
| 23 | Nemo-Aging... | Nemo | Nov 2009 | wild type | 1 | 11 | 040 | | missing video | 0 | 29.9500 | 18.1636 | 0.0202 | ☑ | ☐ | 5 | ☑ |
| 24 | Nemo-Aging... | Nemo | Nov 2009 | wild type | 1 | 11 | 041 | | missing video | 0 | 29.9500 | 18.1636 | 0.0201 | ☑ | ☐ | 5 | ☑ |
| 25 | Nemo-Aging... | Nemo | Nov 2009 | wild type | 1 | 11 | 042 | | missing video | 0 | 29.9500 | 18.1636 | 0.0201 | ☑ | ☐ | 5 | ☑ |
| 26 | Nemo-Aging... | Nemo | Nov 2009 | wild type | 1 | 11 | 043 | | missing video | 0 | 29.9500 | 18.1636 | 0.0202 | ☑ | ☐ | 5 | ☑ |
| 27 | Nemo-Aging... | Nemo | Nov 2009 | wild type | 1 | 11 | 044 | | missing video | 0 | 29.9500 | 18.1636 | 0.0200 | ☑ | ☐ | 5 | ☑ |
| 28 | Nemo-Aging... | Nemo | Nov 2009 | wild type | 1 | 11 | 045 | | missing video | 0 | 29.9500 | 18.1636 | 0.0202 | ☑ | ☐ | 2 | ☑ |
| 29 | Nemo-Aging... | Nemo | Nov 2009 | wild type | 1 | 11 | 046 | | missing video | 0 | 29.9500 | 18.1636 | 0.0200 | ☑ | ☐ | 4 | ☑ |
| 30 | Nemo-Aging... | Nemo | Nov 2009 | wild type | 1 | 11 | 047 | | missing video | 0 | 29.9500 | 18.1636 | 0.0201 | ☑ | ☐ | 5 | ☑ |
| 31 | Nemo-Aging... | Nemo | Nov 2009 | wild type | 1 | 11 | 048 | | missing video | 0 | 29.9500 | 18.1636 | 0.0200 | ☑ | ☐ | 4 | ☑ |
| 32 | Nemo-Aging... | Nemo | Nov 2009 | wild type | 1 | 13 | 049 | | missing video | 0 | 29.9500 | 18.1636 | 0.0200 | ☑ | ☐ | 4 | ☑ |
| 33 | Nemo-Aging... | Nemo | Nov 2009 | wild type | 1 | 13 | 050 | | missing video | 0 | 29.9500 | 18.1636 | 0.0202 | ☑ | ☐ | 4 | ☑ |
| 34 | Nemo-Aging... | Nemo | Nov 2009 | wild type | 1 | 13 | 051 | | missing video | 0 | 29.9500 | 18.1636 | 0.0199 | ☑ | ☐ | 3 | ☑ |
| 35 | Nemo-Aging... | Nemo | Nov 2009 | wild type | 1 | 13 | 052 | | missing video | 0 | 29.9500 | 18.1636 | 0.0200 | ☑ | ☐ | 4 | ☑ |
| 36 | Nemo-Aging... | Nemo | Nov 2009 | wild type | 1 | 13 | 053 | | missing video | 0 | 29.9500 | 18.1636 | 0.0199 | ☑ | ☐ | 5 | ☑ |
| 37 | Nemo-Aging... | Nemo | Nov 2009 | wild type | 1 | 13 | 054 | | missing video | 0 | 29.9500 | 18.1636 | 0.0200 | ☑ | ☐ | 5 | ☑ |
| 38 | Nemo-Aging... | Nemo | Nov 2009 | wild type | 1 | 13 | 055 | | missing video | 0 | 29.9500 | 18.1636 | 0.0199 | ☑ | ☐ | 4 | ☑ |
| 39 | Nemo-Aging... | Nemo | Nov 2009 | wild type | 1 | 13 | 056 | | missing video | 0 | 29.9500 | 18.1636 | 0.0201 | ☑ | ☐ | 5 | ☑ |
| 40 | Nemo-Aging... | Nemo | Nov 2009 | wild type | 1 | 13 | 057 | | missing video | 0 | 29.9500 | 18.1636 | 0.0201 | ☑ | ☐ | 5 | ☑ |
| 41 | Nemo-Aging... | Nemo | Nov 2009 | wild type | 1 | 13 | 058 | | missing video | 0 | 29.9500 | 18.1636 | 0.0200 | ☑ | ☐ | 3 | ☑ |
| 42 | Nemo-Aging... | Nemo | Nov 2009 | wild type | 1 | 13 | 059 | | missing video | 0 | 29.9500 | 18.1636 | 0.0200 | ☑ | ☐ | 5 | ☑ |
| 43 | Nemo-Aging... | Nemo | Nov 2009 | wild type | 1 | 13 | 060 | | missing video | 0 | 29.9500 | 18.1636 | 0.0201 | ☑ | ☐ | 5 | ☑ |
| 44 | Nemo-Aging... | Nemo | Nov 2009 | wild type | 1 | 15 | 061 | | missing video | 0 | 29.9500 | 18.1636 | 0.0201 | ☑ | ☐ | 4 | ☑ |
| 45 | Nemo-Aging... | Nemo | Nov 2009 | wild type | 1 | 15 | 062 | | missing video | 0 | 29.9500 | 18.1636 | 0.0200 | ☑ | ☐ | 5 | ☑ |
| 46 | Nemo-Aging... | Nemo | Nov 2009 | wild type | 1 | 15 | 063 | | missing video | 0 | 29.9500 | 18.1636 | 0.0202 | ☑ | ☐ | 5 | ☑ |

**Since the video files are too large for distribution, the first two functions are disabled on this demonstration.**
**See the other demonstration for details on those functions.**
**For this demonstration, start with the Display function.**

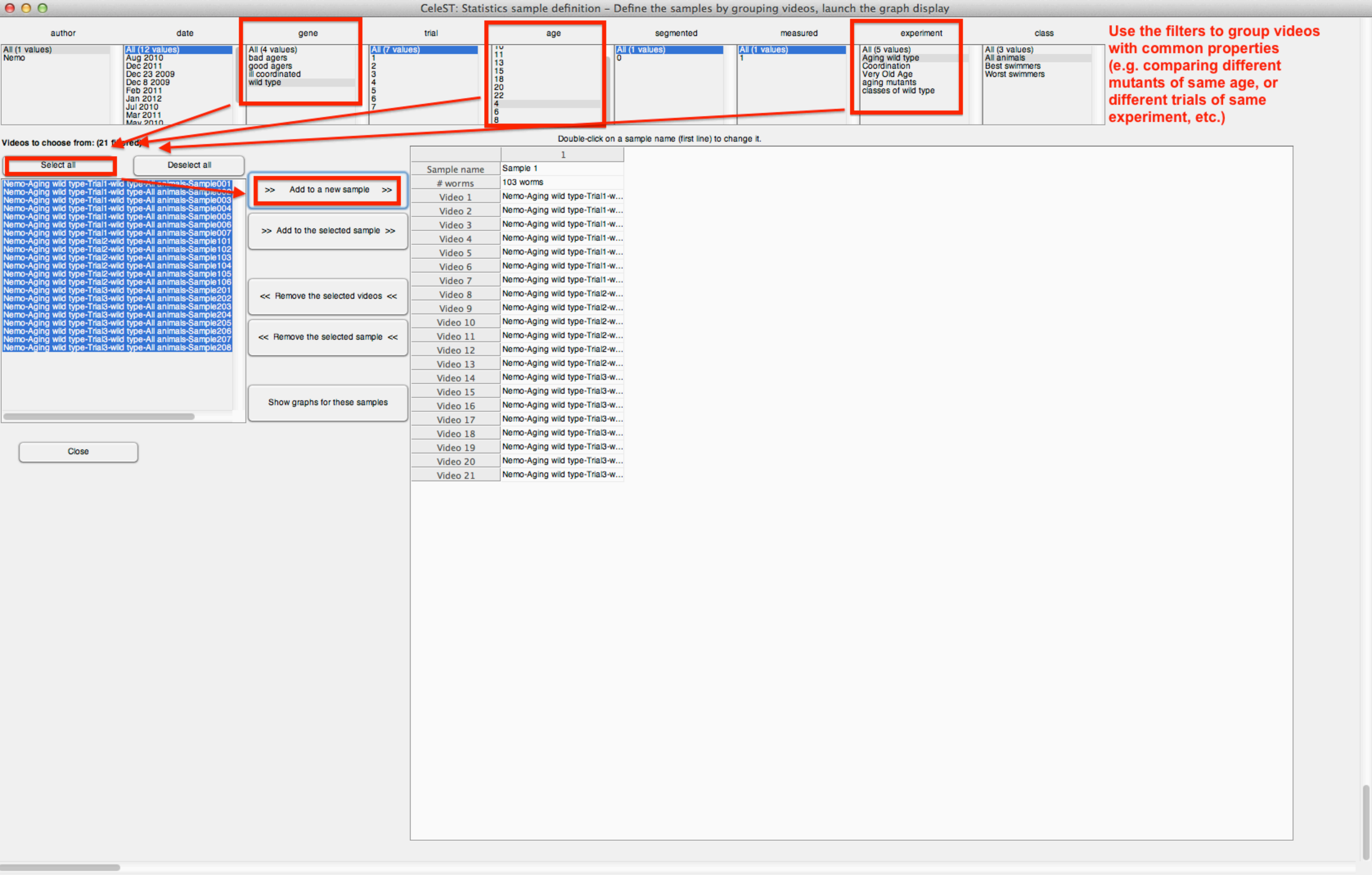
CeleST: Statistics sample definition – Define the samples by grouping videos, launch the graph display
author
date
gene
trial
age
segmented
measured
experiment
class
All (1 values)
Nemo
All (12 values)
Aug 2010
Dec 2011
Dec 23 2009
Dec 8 2009
Feb 2011
Jan 2012
Jul 2010
Mar 2011
May 2010
All (4 values)
bad agers
good agers
ill coordinated
wild type
All (7 values)
1
2
3
4
5
6
7
11
13
15
18
20
22
4
6
8
All (1 values)
0
All (1 values)
1
All (5 values)
Aging wild type
Coordination
Very Old Age
aging mutants
classes of wild type
All (3 values)
All animals
Best swimmers
Worst swimmers
Use the filters to group videos with common properties (e.g. comparing different mutants of same age, or different trials of same experiment, etc.)
Videos to choose from: (21 filtered)
Select all
Deselect all
Nemo-Aging wild type-Trial1-wild type-All animals-Sample001
Nemo-Aging wild type-Trial1-wild type-All animals-Sample002
Nemo-Aging wild type-Trial1-wild type-All animals-Sample003
Nemo-Aging wild type-Trial1-wild type-All animals-Sample004
Nemo-Aging wild type-Trial1-wild type-All animals-Sample005
Nemo-Aging wild type-Trial1-wild type-All animals-Sample006
Nemo-Aging wild type-Trial1-wild type-All animals-Sample007
Nemo-Aging wild type-Trial2-wild type-All animals-Sample101
Nemo-Aging wild type-Trial2-wild type-All animals-Sample102
Nemo-Aging wild type-Trial2-wild type-All animals-Sample103
Nemo-Aging wild type-Trial2-wild type-All animals-Sample104
Nemo-Aging wild type-Trial2-wild type-All animals-Sample105
Nemo-Aging wild type-Trial2-wild type-All animals-Sample106
Nemo-Aging wild type-Trial3-wild type-All animals-Sample201
Nemo-Aging wild type-Trial3-wild type-All animals-Sample202
Nemo-Aging wild type-Trial3-wild type-All animals-Sample203
Nemo-Aging wild type-Trial3-wild type-All animals-Sample204
Nemo-Aging wild type-Trial3-wild type-All animals-Sample205
Nemo-Aging wild type-Trial3-wild type-All animals-Sample206
Nemo-Aging wild type-Trial3-wild type-All animals-Sample207
Nemo-Aging wild type-Trial3-wild type-All animals-Sample208
>> Add to a new sample >>
>> Add to the selected sample >>
<< Remove the selected videos <<
<< Remove the selected sample <<
Show graphs for these samples
Close
Double-click on a sample name (first line) to change it.
1
Sample name
Sample 1
# worms
103 worms
Video 1
Nemo-Aging wild type-Trial1-w...
Video 2
Nemo-Aging wild type-Trial1-w...
Video 3
Nemo-Aging wild type-Trial1-w...
Video 4
Nemo-Aging wild type-Trial1-w...
Video 5
Nemo-Aging wild type-Trial1-w...
Video 6
Nemo-Aging wild type-Trial1-w...
Video 7
Nemo-Aging wild type-Trial1-w...
Video 8
Nemo-Aging wild type-Trial2-w...
Video 9
Nemo-Aging wild type-Trial2-w...
Video 10
Nemo-Aging wild type-Trial2-w...
Video 11
Nemo-Aging wild type-Trial2-w...
Video 12
Nemo-Aging wild type-Trial2-w...
Video 13
Nemo-Aging wild type-Trial2-w...
Video 14
Nemo-Aging wild type-Trial3-w...
Video 15
Nemo-Aging wild type-Trial3-w...
Video 16
Nemo-Aging wild type-Trial3-w...
Video 17
Nemo-Aging wild type-Trial3-w...
Video 18
Nemo-Aging wild type-Trial3-w...
Video 19
Nemo-Aging wild type-Trial3-w...
Video 20
Nemo-Aging wild type-Trial3-w...
Video 21
Nemo-Aging wild type-Trial3-w...

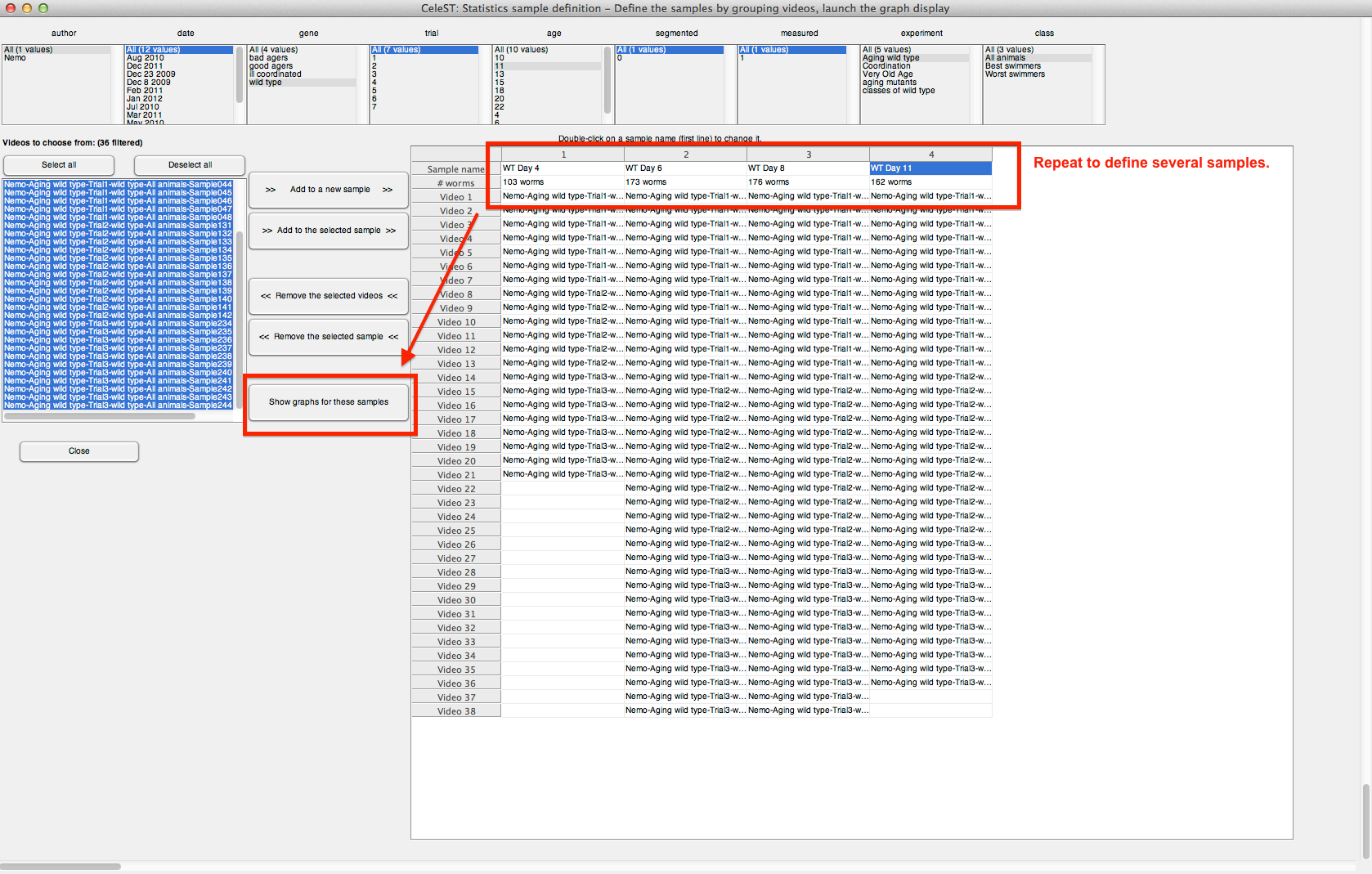
CeleST: Statistics sample definition – Define the samples by grouping videos, launch the graph display
author
date
gene
trial
age
segmented
measured
experiment
class
All (1 values)
Nemo
All (12 values)
Aug 2010
Dec 2011
Dec 23 2009
Dec 8 2009
Feb 2011
Jan 2012
Jul 2010
Mar 2011
All (4 values)
bad agers
good agers
ill coordinated
wild type
All (7 values)
All (10 values)
All (1 values)
All (5 values)
Aging wild type
Coordination
Very Old Age
aging mutants
classes of wild type
All (3 values)
All animals
Best swimmers
Worst swimmers
Videos to choose from: (36 filtered)
Select all
Deselect all
Nemo-Aging wild type-Trial1-wild type-All animals-Sample044
>> Add to a new sample >>
>> Add to the selected sample >>
<< Remove the selected videos <<
<< Remove the selected sample <<
Show graphs for these samples
Close
Double-click on a sample name (first line) to change it.
Sample name
# worms
WT Day 4
WT Day 6
WT Day 8
WT Day 11
103 worms
173 worms
176 worms
162 worms
Nemo-Aging wild type-Trial1-w...
Repeat to define several samples.

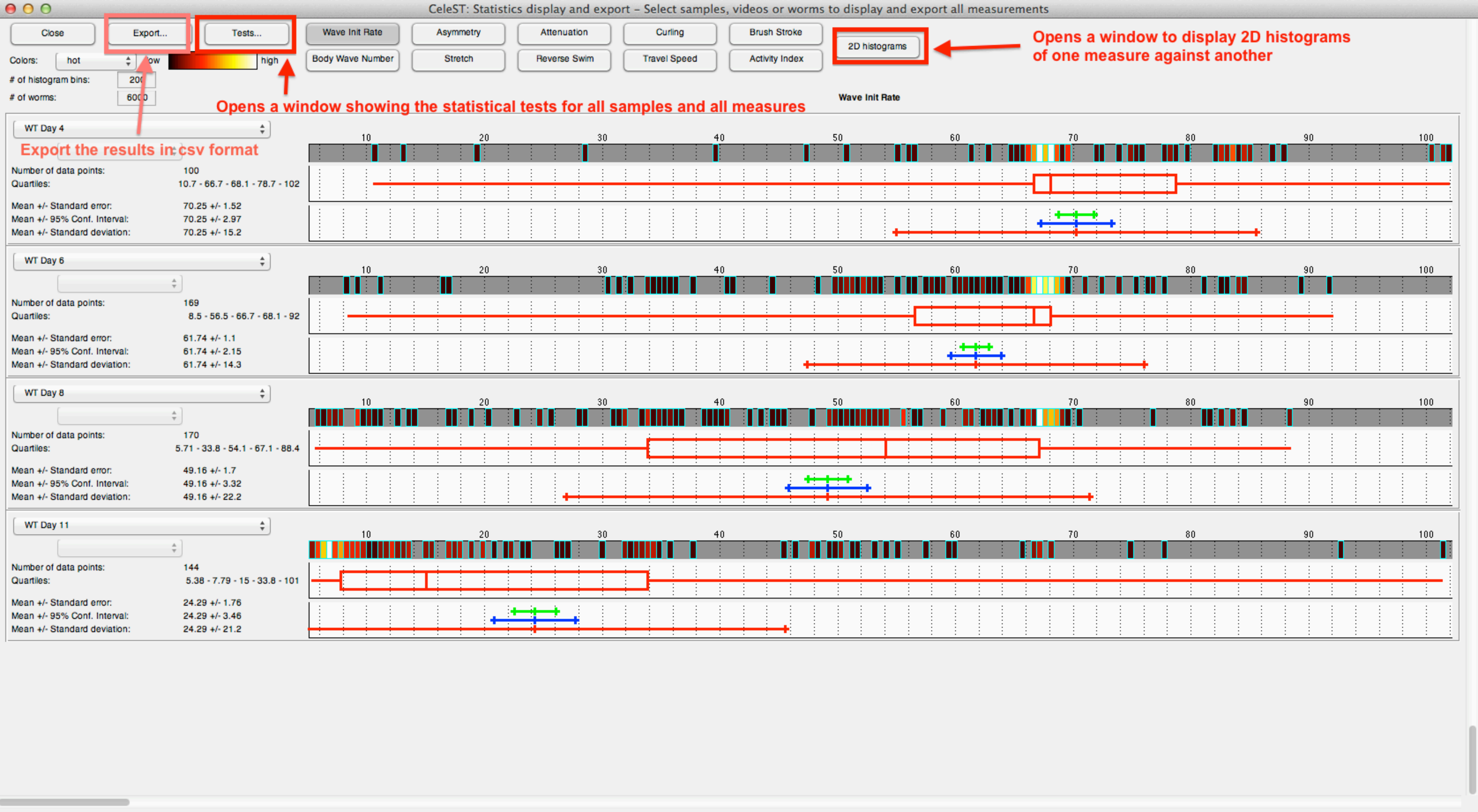
CeleST: Statistics display and export – Select samples, videos or worms to display and export all measurements
Close
Export...
Tests...
Wave Init Rate
Asymmetry
Attenuation
Curling
Brush Stroke
2D histograms
Opens a window to display 2D histograms of one measure against another
Colors:
hot
low
high
Body Wave Number
Stretch
Reverse Swim
Travel Speed
Activity Index
# of histogram bins:
# of worms:
Opens a window showing the statistical tests for all samples and all measures
Wave Init Rate
Export the results in csv format
WT Day 4
Number of data points: 100
Quartiles: 10.7 - 66.7 - 68.1 - 78.7 - 102
Mean +/- Standard error: 70.25 +/- 1.52
Mean +/- 95% Conf. Interval: 70.25 +/- 2.97
Mean +/- Standard deviation: 70.25 +/- 15.2
WT Day 6
Number of data points: 169
Quartiles: 8.5 - 56.5 - 66.7 - 68.1 - 92
Mean +/- Standard error: 61.74 +/- 1.1
Mean +/- 95% Conf. Interval: 61.74 +/- 2.15
Mean +/- Standard deviation: 61.74 +/- 14.3
WT Day 8
Number of data points: 170
Quartiles: 5.71 - 33.8 - 54.1 - 67.1 - 88.4
Mean +/- Standard error: 49.16 +/- 1.7
Mean +/- 95% Conf. Interval: 49.16 +/- 3.32
Mean +/- Standard deviation: 49.16 +/- 22.2
WT Day 11
Number of data points: 144
Quartiles: 5.38 - 7.79 - 15 - 33.8 - 101
Mean +/- Standard error: 24.29 +/- 1.76
Mean +/- 95% Conf. Interval: 24.29 +/- 3.46
Mean +/- Standard deviation: 24.29 +/- 21.2
10
20
30
40
50
60
70
80
90
100

CeleST: Statistics tests display and export

Close

| | | | | | | | | | | |
|---|---|---|---|---|---|---|---|---|---|---|
| p values | | WT Day 6 | | | WT Day 8 | | | WT Day 11 | | |
| WT Day 4 | | F test | t test equal | t test diff. | F test | t test equal | t test diff. | F test | t test equal | t test diff. |
| | Body Wave N... | 0.0141 | 0.0384 | 0.0284 | 0.0000 | 0.0000 | 0.0000 | 0.0000 | 0.0000 | 0.0000 |
| | Asymmetry | 0.1427 | 0.0345 | 0.0288 | 0.0000 | 0.7052 | 0.6687 | 0.0000 | 0.3698 | 0.2940 |
| | Stretch | 0.0091 | 0.8988 | 0.8924 | 0.0000 | 0.0000 | 0.0000 | 0.0000 | 0.0000 | 0.0000 |
| | Attenuation | 0.3507 | 0.5903 | 0.5822 | 0.0000 | 0.0000 | 0.0000 | 0.0000 | 0.0021 | 0.0007 |
| | Reverse Swim | 0.9010 | 0.3314 | 0.3301 | 0.0000 | 0.0001 | 0.0000 | 0.0000 | 0.0000 | 0.0000 |
| | Curling | 0.0000 | 0.1851 | 0.2488 | 0.0000 | 0.0014 | 0.0002 | 0.0000 | 0.0004 | 0.0001 |
| | Travel Speed | 0.0126 | 0.0889 | 0.1081 | 0.1211 | 0.0000 | 0.0000 | 0.0000 | 0.0000 | 0.0000 |
| | Brush Stroke | 0.0666 | 0.4349 | 0.4539 | 0.5577 | 0.0000 | 0.0000 | 0.0012 | 0.0000 | 0.0000 |
| | Activity Index | 0.0004 | 0.0014 | 0.0033 | 0.2000 | 0.0000 | 0.0000 | 0.9424 | 0.0000 | 0.0000 |
| | | | | | | | | | | |
| WT Day 6 | | | | | F test | t test equal | t test diff. | F test | t test equal | t test diff. |
| | Body Wave N... | | | | 0.0000 | 0.0000 | 0.0000 | 0.0000 | 0.0000 | 0.0000 |
| | Asymmetry | | | | 0.0000 | 0.1518 | 0.1514 | 0.0000 | 0.0365 | 0.0510 |
| | Stretch | | | | 0.0000 | 0.0000 | 0.0000 | 0.0000 | 0.0000 | 0.0000 |
| | Attenuation | | | | 0.0000 | 0.0000 | 0.0000 | 0.0000 | 0.0009 | 0.0015 |
| | Reverse Swim | | | | 0.0000 | 0.0002 | 0.0002 | 0.0000 | 0.0000 | 0.0001 |
| | Curling | | | | 0.0000 | 0.0000 | 0.0000 | 0.0000 | 0.0000 | 0.0000 |
| | Travel Speed | | | | 0.2818 | 0.0000 | 0.0000 | 0.0013 | 0.0000 | 0.0000 |
| | Brush Stroke | | | | 0.0054 | 0.0000 | 0.0000 | 0.0975 | 0.0000 | 0.0000 |
| | Activity Index | | | | 0.0000 | 0.0000 | 0.0000 | 0.0001 | 0.0000 | 0.0000 |
| | | | | | | | | | | |
| WT Day 8 | | | | | | | | F test | t test equal | t test diff. |
| | Body Wave N... | | | | | | | 0.0130 | 0.0000 | 0.0000 |
| | Asymmetry | | | | | | | 0.0000 | 0.1949 | 0.2207 |
| | Stretch | | | | | | | 0.0008 | 0.1706 | 0.1801 |
| | Attenuation | | | | | | | 0.0118 | 0.6789 | 0.6839 |
| | Reverse Swim | | | | | | | 0.7135 | 0.6529 | 0.6537 |
| | Curling | | | | | | | 0.0000 | 0.1039 | 0.1172 |
| | Travel Speed | | | | | | | 0.0000 | 0.0000 | 0.0000 |
| | Brush Stroke | | | | | | | 0.0000 | 0.0000 | 0.0000 |
| | Activity Index | | | | | | | 0.1284 | 0.0000 | 0.0000 |

**This table lists the p-values of the three standard statistical tests:**

- **F test (for equal variance)**
- **t-test assuming equal variance (for equal mean)**
- **t-test assuming unequal variance (for equal mean)**

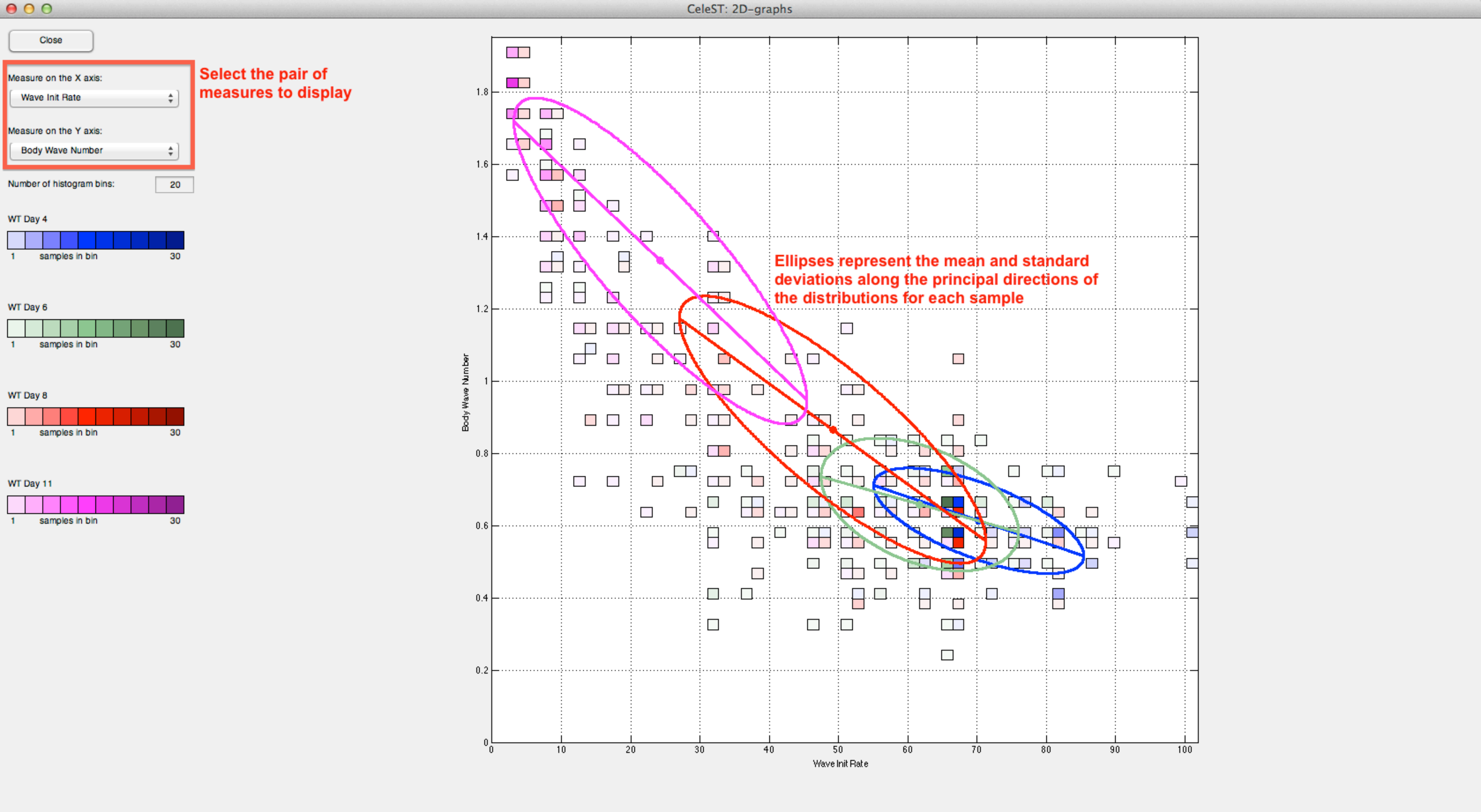
CeleST: 2D-graphs
Close
Measure on the X axis:
Wave Init Rate
Measure on the Y axis:
Body Wave Number
Select the pair of measures to display
Number of histogram bins:
20
WT Day 4
1 samples in bin 30
WT Day 6
1 samples in bin 30
WT Day 8
1 samples in bin 30
WT Day 11
1 samples in bin 30
Ellipses represent the mean and standard deviations along the principal directions of the distributions for each sample
Body Wave Number
Wave Init Rate
0
0.2
0.4
0.6
0.8
1
1.2
1.4
1.6
1.8
0
10
20
30
40
50
60
70
80
90
100

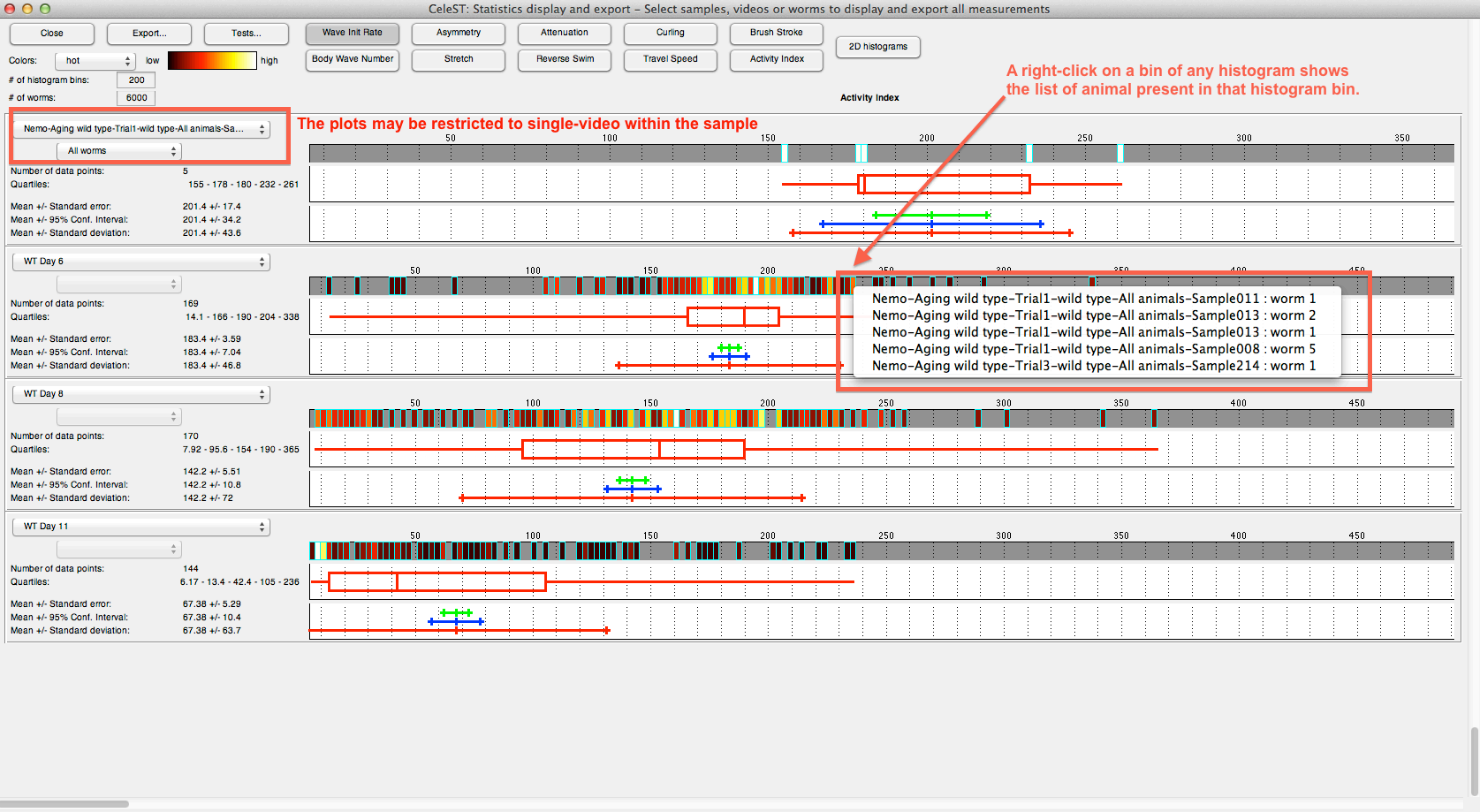

CeleST: Statistics display and export – Select samples, videos or worms to display and export all measurements
Close
Export...
Tests...
Wave Init Rate
Asymmetry
Attenuation
Curling
Brush Stroke
2D histograms
Body Wave Number
Stretch
Reverse Swim
Travel Speed
Activity Index
Colors: hot
low
high
# of histogram bins: 200
# of worms: 6000
A right-click on a bin of any histogram shows the list of animal present in that histogram bin.
Activity Index
Nemo-Aging wild type-Trial1-wild type-All animals-Sa...
All worms
The plots may be restricted to single-video within the sample
Number of data points: 5
Quartiles: 155 - 178 - 180 - 232 - 261
Mean +/- Standard error: 201.4 +/- 17.4
Mean +/- 95% Conf. Interval: 201.4 +/- 34.2
Mean +/- Standard deviation: 201.4 +/- 43.6
WT Day 6
Number of data points: 169
Quartiles: 14.1 - 166 - 190 - 204 - 338
Mean +/- Standard error: 183.4 +/- 3.59
Mean +/- 95% Conf. Interval: 183.4 +/- 7.04
Mean +/- Standard deviation: 183.4 +/- 46.8
Nemo-Aging wild type-Trial1-wild type-All animals-Sample011 : worm 1
Nemo-Aging wild type-Trial1-wild type-All animals-Sample013 : worm 2
Nemo-Aging wild type-Trial1-wild type-All animals-Sample013 : worm 1
Nemo-Aging wild type-Trial1-wild type-All animals-Sample008 : worm 5
Nemo-Aging wild type-Trial3-wild type-All animals-Sample214 : worm 1
WT Day 8
Number of data points: 170
Quartiles: 7.92 - 95.6 - 154 - 190 - 365
Mean +/- Standard error: 142.2 +/- 5.51
Mean +/- 95% Conf. Interval: 142.2 +/- 10.8
Mean +/- Standard deviation: 142.2 +/- 72
WT Day 11
Number of data points: 144
Quartiles: 6.17 - 13.4 - 42.4 - 105 - 236
Mean +/- Standard error: 67.38 +/- 5.29
Mean +/- 95% Conf. Interval: 67.38 +/- 10.4
Mean +/- Standard deviation: 67.38 +/- 63.7
