# Supplementary material for: CeleST: Computer Vision Software for Quantitative Analysis of C. elegans Swim Behavior Reveals Novel Features of Locomotion
Source: PLoS Comput Biol. 2014 Jul 17;10(7):e1003702. doi: 10.1371/journal.pcbi.1003702 (PMC4102393; doi:10.1371/journal.pcbi.1003702)
Supplement: Dataset S1 — Zip package containing source code and demos of the software CeleST. (ZIP) [file pcbi.1003702.s008.zip › CeleST submission small/demos/CeleST - demo with videos/User guide - videos.pdf]

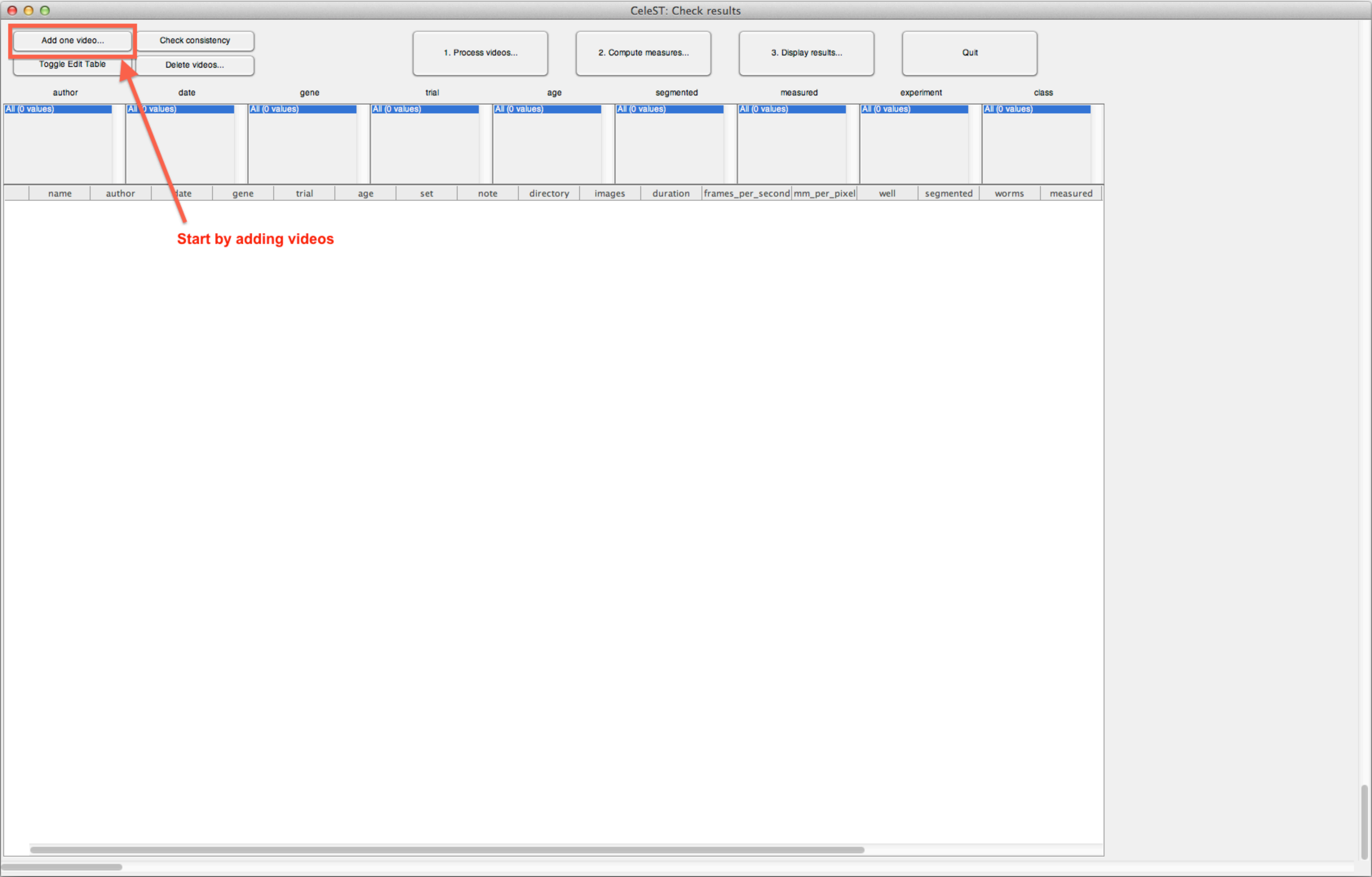
CeleST: Check results
Add one video...
Check consistency
Toggle Edit Table
Delete videos...
1. Process videos...
2. Compute measures...
3. Display results...
Quit
author
date
gene
trial
age
segmented
measured
experiment
class
All (0 values)
name
author
date
gene
trial
age
set
note
directory
images
duration
frames_per_second
mm_per_pixel
well
segmented
worms
measured
Start by adding videos

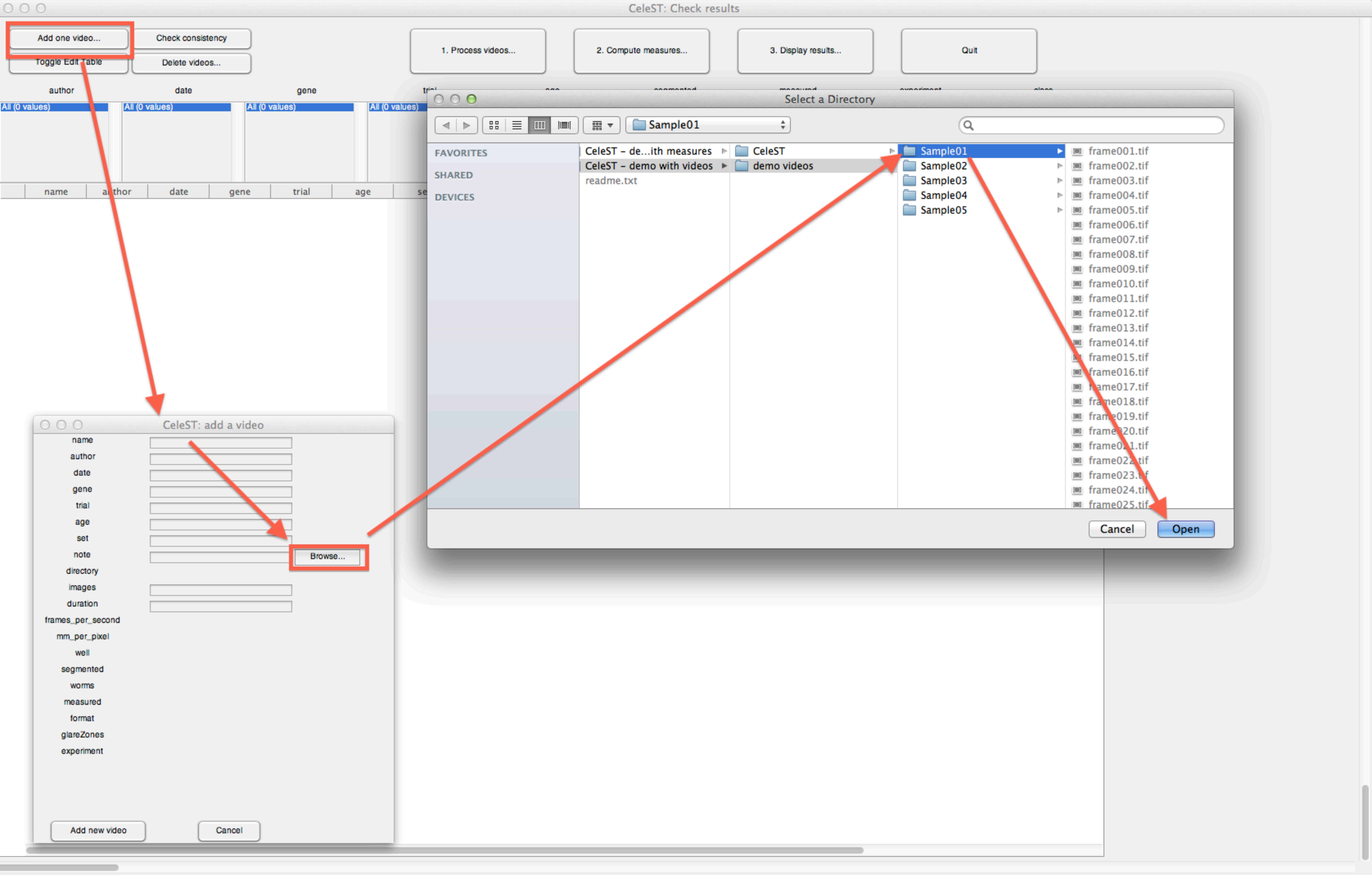
CeleST: Check results
Add one video...
Check consistency
Toggle Edit Table
Delete videos...
1. Process videos...
2. Compute measures...
3. Display results...
Quit
author
date
gene
All (0 values)
name
author
date
gene
trial
age
Select a Directory
Sample01
FAVORITES
SHARED
DEVICES
CeleST - de...ith measures
CeleST - demo with videos
readme.txt
CeleST
demo videos
Sample01
Sample02
Sample03
Sample04
Sample05
frame001.tif
frame002.tif
frame003.tif
frame004.tif
frame005.tif
frame006.tif
frame007.tif
frame008.tif
frame009.tif
frame010.tif
frame011.tif
frame012.tif
frame013.tif
frame014.tif
frame015.tif
frame016.tif
Cancel
Open
CeleST: add a video
name
author
date
gene
trial
age
set
note
Browse...
directory
images
duration
frames_per_second
mm_per_pixel
well
segmented
worms
measured
format
glareZones
experiment
Add new video
Cancel

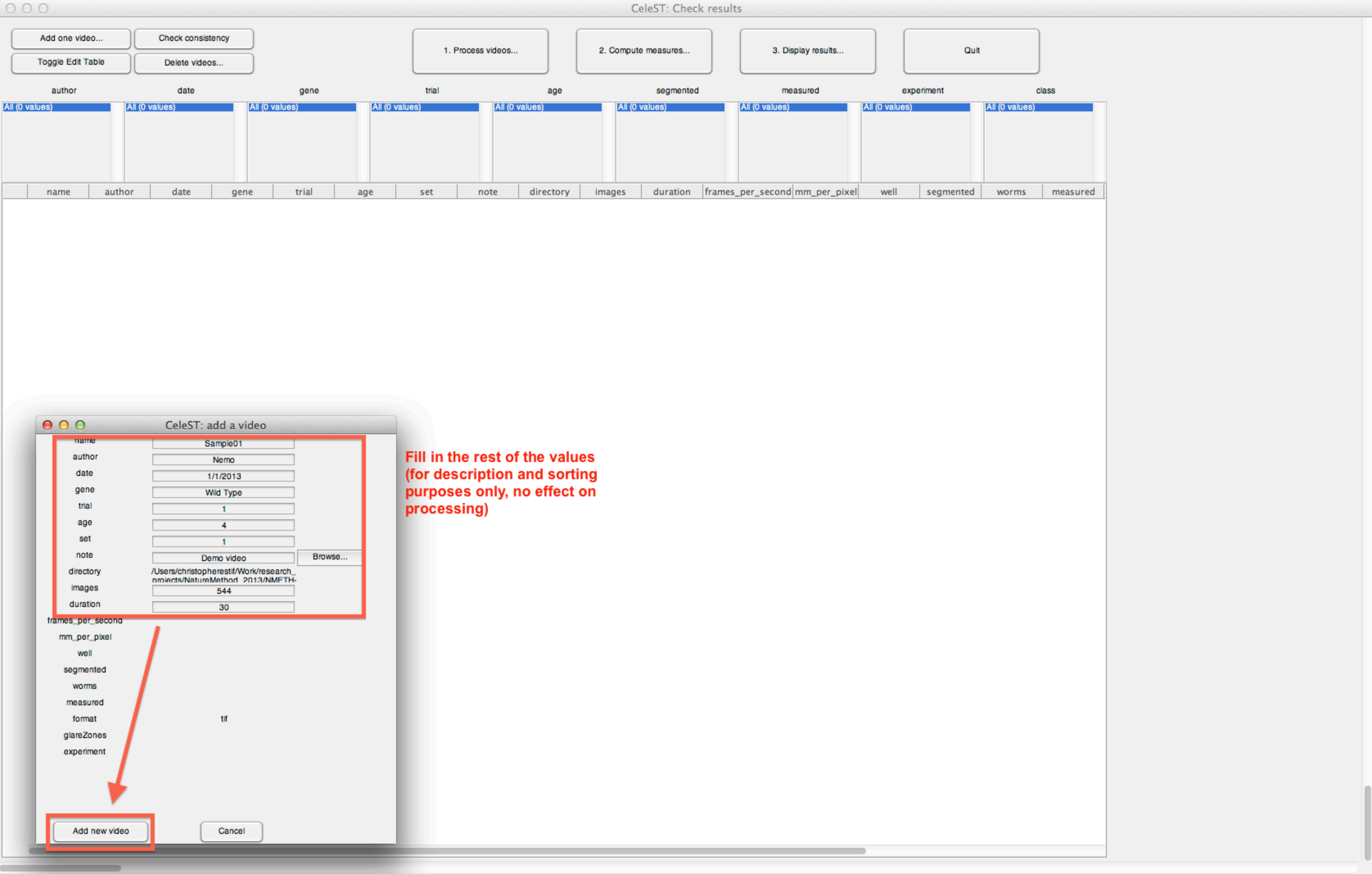
CeleST: Check results
Add one video...
Check consistency
Toggle Edit Table
Delete videos...
1. Process videos...
2. Compute measures...
3. Display results...
Quit
author
date
gene
trial
age
segmented
measured
experiment
class
All (0 values)
name
author
date
gene
trial
age
set
note
directory
images
duration
frames_per_second
mm_per_pixel
well
segmented
worms
measured
CeleST: add a video
name
Sample01
author
Nemo
date
1/1/2013
gene
Wild Type
trial
1
age
4
set
1
note
Demo video
Browse...
directory
/Users/christopherestif/Work/research_
images
544
duration
30
frames_per_second
mm_per_pixel
well
segmented
worms
measured
format
tif
glareZones
experiment
Add new video
Cancel
Fill in the rest of the values (for description and sorting purposes only, no effect on processing)

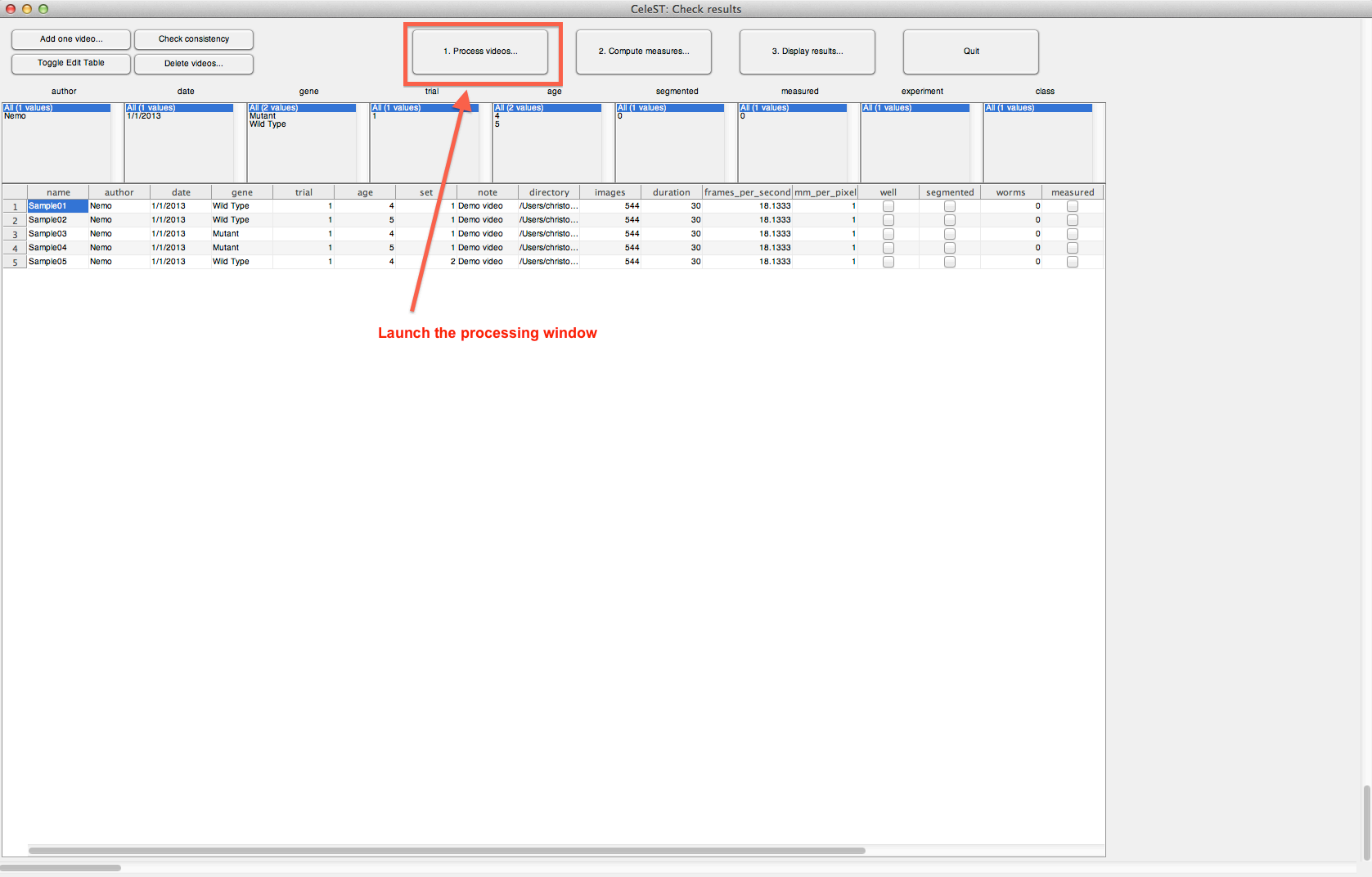
CeleST: Check results
Add one video...
Check consistency
Toggle Edit Table
Delete videos...
1. Process videos...
2. Compute measures...
3. Display results...
Quit
author
date
gene
trial
age
segmented
measured
experiment
class
All (1 values)
Nemo
1/1/2013
All (2 values)
Mutant
Wild Type
name
author
date
gene
trial
age
set
note
directory
images
duration
frames_per_second
mm_per_pixel
well
segmented
worms
measured
1 Sample01 Nemo 1/1/2013 Wild Type 1 4 1 Demo video /Users/christo... 544 30 18.1333 1 0
2 Sample02 Nemo 1/1/2013 Wild Type 1 5 1 Demo video /Users/christo... 544 30 18.1333 1 0
3 Sample03 Nemo 1/1/2013 Mutant 1 4 1 Demo video /Users/christo... 544 30 18.1333 1 0
4 Sample04 Nemo 1/1/2013 Mutant 1 5 1 Demo video /Users/christo... 544 30 18.1333 1 0
5 Sample05 Nemo 1/1/2013 Wild Type 1 4 2 Demo video /Users/christo... 544 30 18.1333 1 0
Launch the processing window

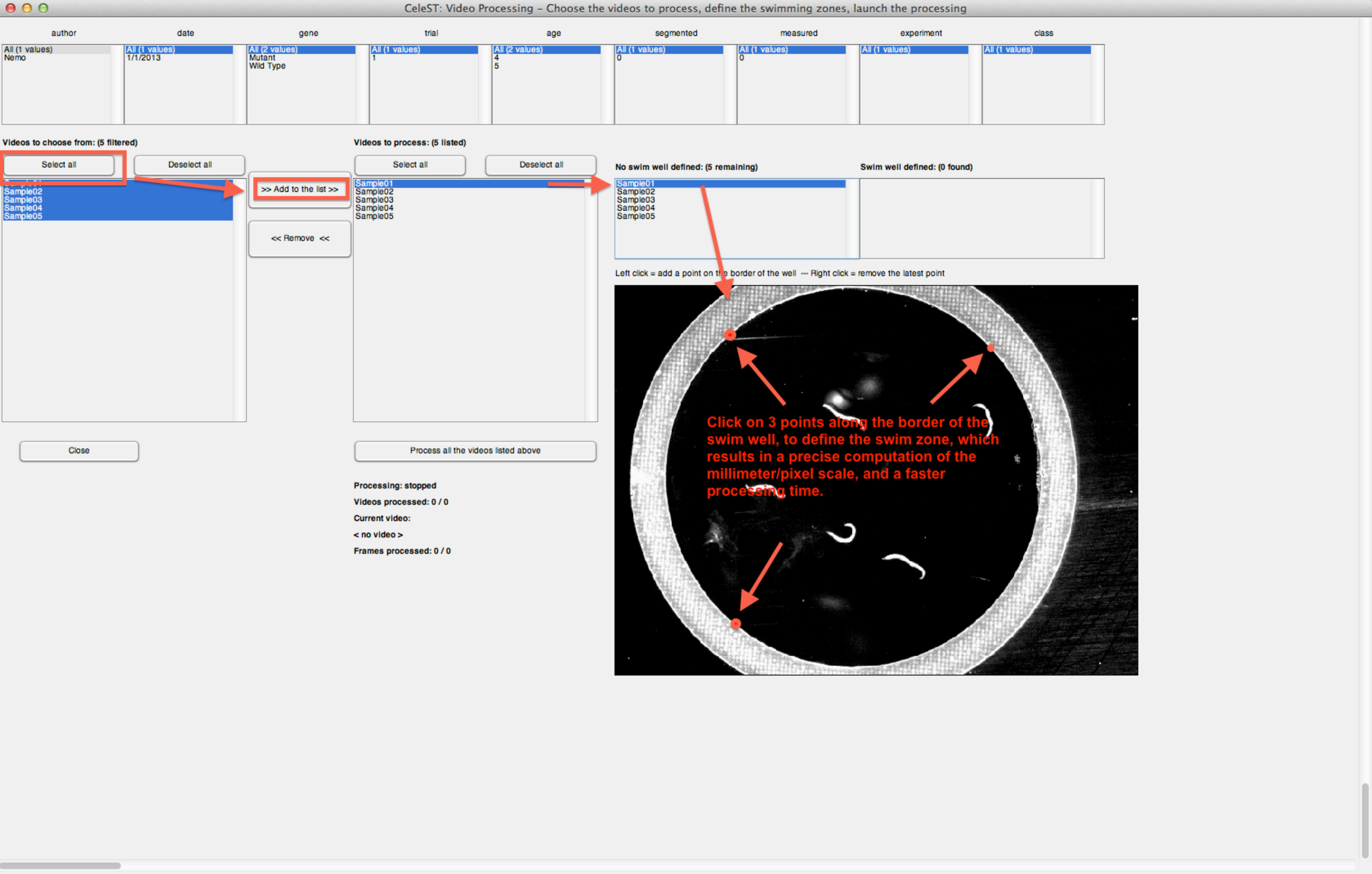
CeleST: Video Processing – Choose the videos to process, define the swimming zones, launch the processing
author
date
gene
trial
age
segmented
measured
experiment
class
All (1 values)
Nemo
All (1 values)
1/1/2013
All (2 values)
Mutant
Wild Type
All (1 values)
1
All (2 values)
4
5
All (1 values)
0
All (1 values)
0
All (1 values)
All (1 values)
Videos to choose from: (5 filtered)
Select all
Deselect all
Sample02
Sample03
Sample04
Sample05
>> Add to the list >>
<< Remove <<
Videos to process: (5 listed)
Select all
Deselect all
Sample01
Sample02
Sample03
Sample04
Sample05
No swim well defined: (5 remaining)
Swim well defined: (0 found)
Sample01
Sample02
Sample03
Sample04
Sample05
Left click = add a point on the border of the well --- Right click = remove the latest point
Click on 3 points along the border of the swim well, to define the swim zone, which results in a precise computation of the millimeter/pixel scale, and a faster processing time.
Close
Process all the videos listed above
Processing: stopped
Videos processed: 0 / 0
Current video:
< no video >
Frames processed: 0 / 0

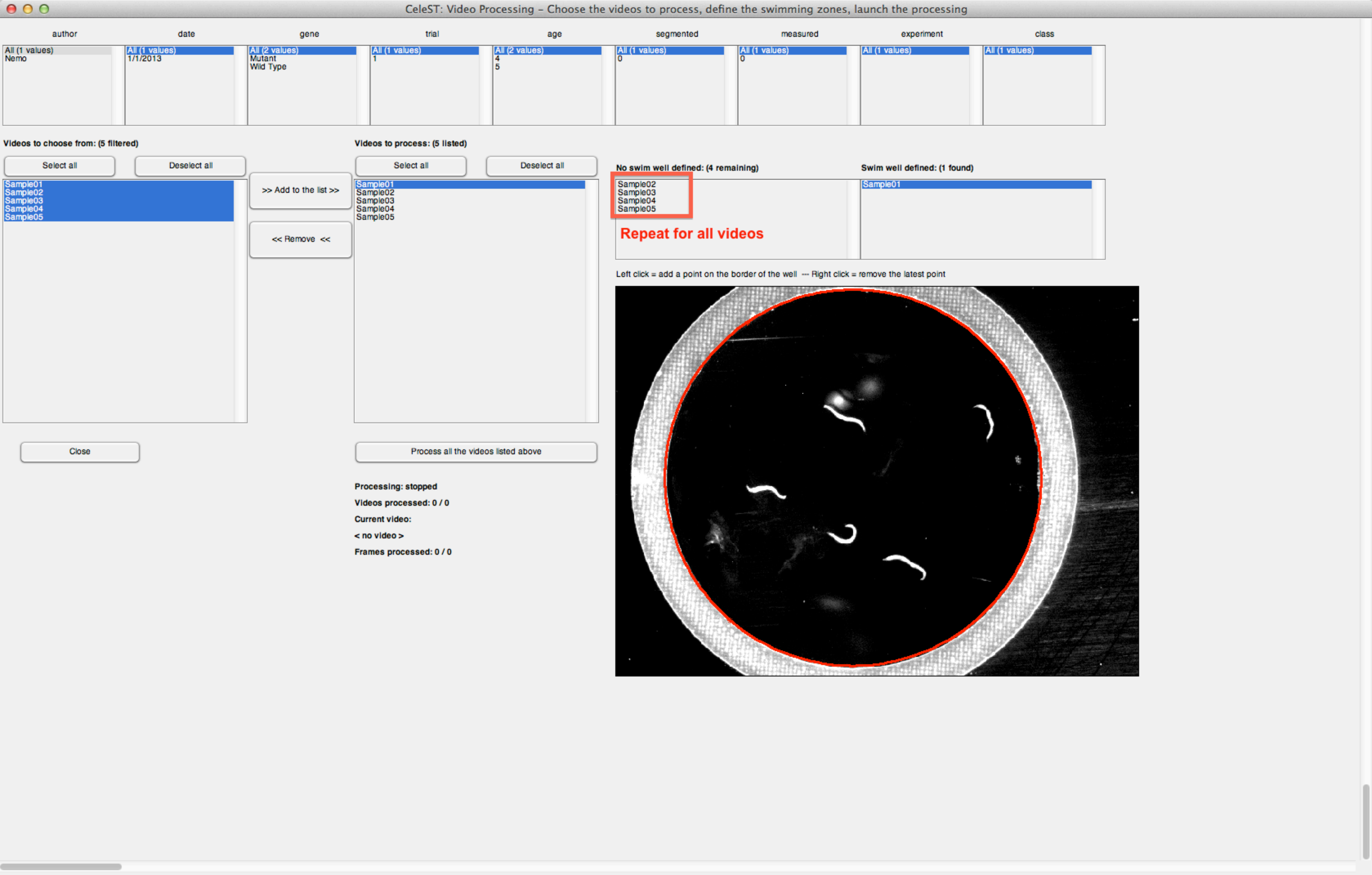

CeleST: Video Processing – Choose the videos to process, define the swimming zones, launch the processing
author
date
gene
trial
age
segmented
measured
experiment
class
All (1 values)
Nemo
All (1 values)
1/1/2013
All (2 values)
Mutant
Wild Type
All (1 values)
1
All (2 values)
4
5
All (1 values)
0
All (1 values)
0
All (1 values)
All (1 values)
Videos to choose from: (5 filtered)
Select all
Deselect all
Sample01
Sample02
Sample03
Sample04
Sample05
>> Add to the list >>
<< Remove <<
Videos to process: (5 listed)
Select all
Deselect all
Sample01
Sample02
Sample03
Sample04
Sample05
No swim well defined: (4 remaining)
Sample02
Sample03
Sample04
Sample05
Repeat for all videos
Swim well defined: (1 found)
Sample01
Left click = add a point on the border of the well --- Right click = remove the latest point
Close
Process all the videos listed above
Processing: stopped
Videos processed: 0 / 0
Current video:
< no video >
Frames processed: 0 / 0

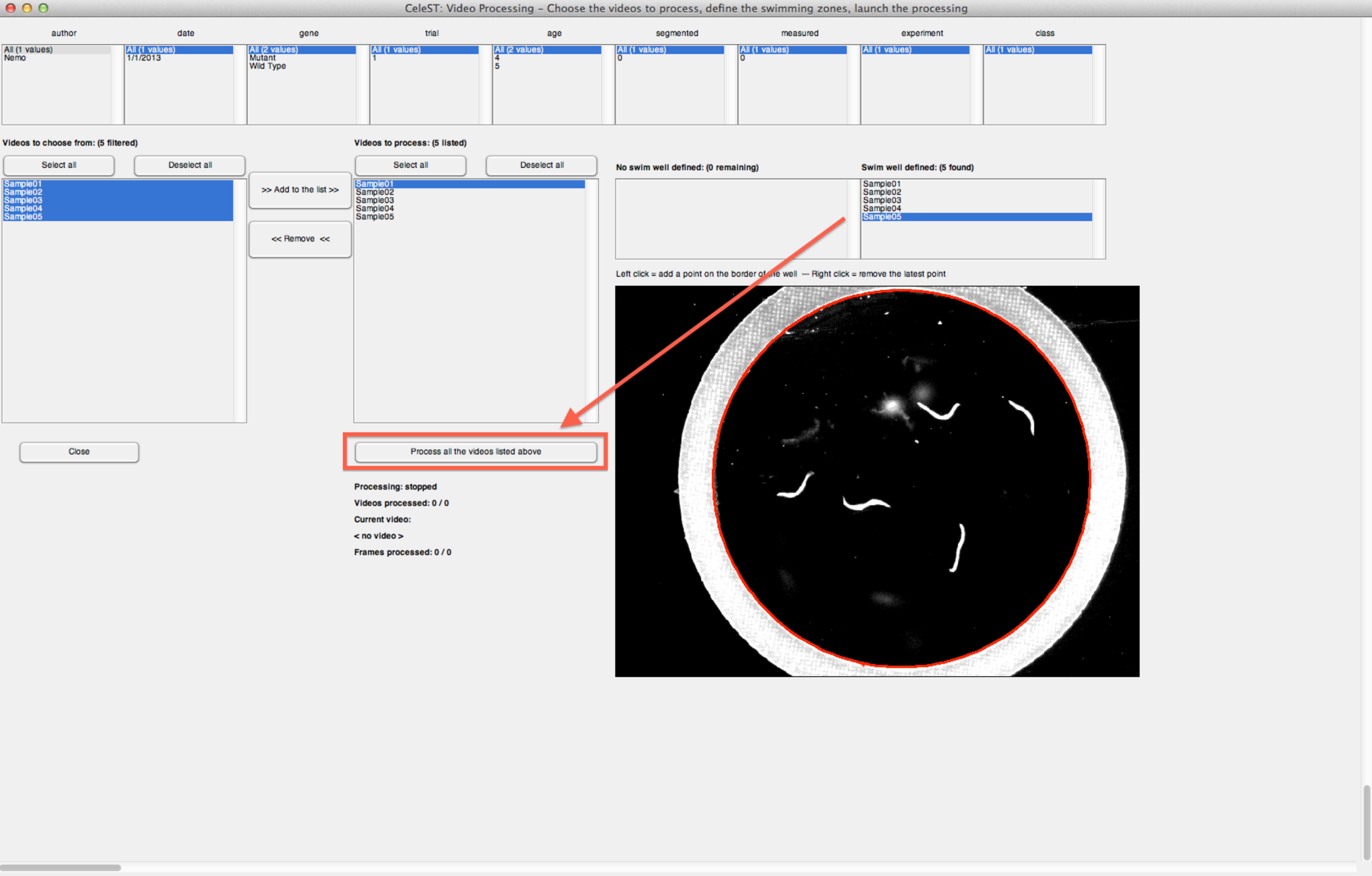
CeleST: Video Processing – Choose the videos to process, define the swimming zones, launch the processing
author
date
gene
trial
age
segmented
measured
experiment
class
All (1 values)
Nemo
All (1 values)
1/1/2013
All (2 values)
Mutant
Wild Type
All (1 values)
1
All (2 values)
4
5
All (1 values)
0
All (1 values)
0
All (1 values)
All (1 values)
Videos to choose from: (5 filtered)
Select all
Deselect all
Sample01
Sample02
Sample03
Sample04
Sample05
>> Add to the list >>
<< Remove <<
Videos to process: (5 listed)
Select all
Deselect all
Sample01
Sample02
Sample03
Sample04
Sample05
No swim well defined: (0 remaining)
Swim well defined: (5 found)
Sample01
Sample02
Sample03
Sample04
Sample05
Left click = add a point on the border of the well --- Right click = remove the latest point
Close
Process all the videos listed above
Processing: stopped
Videos processed: 0 / 0
Current video:
< no video >
Frames processed: 0 / 0

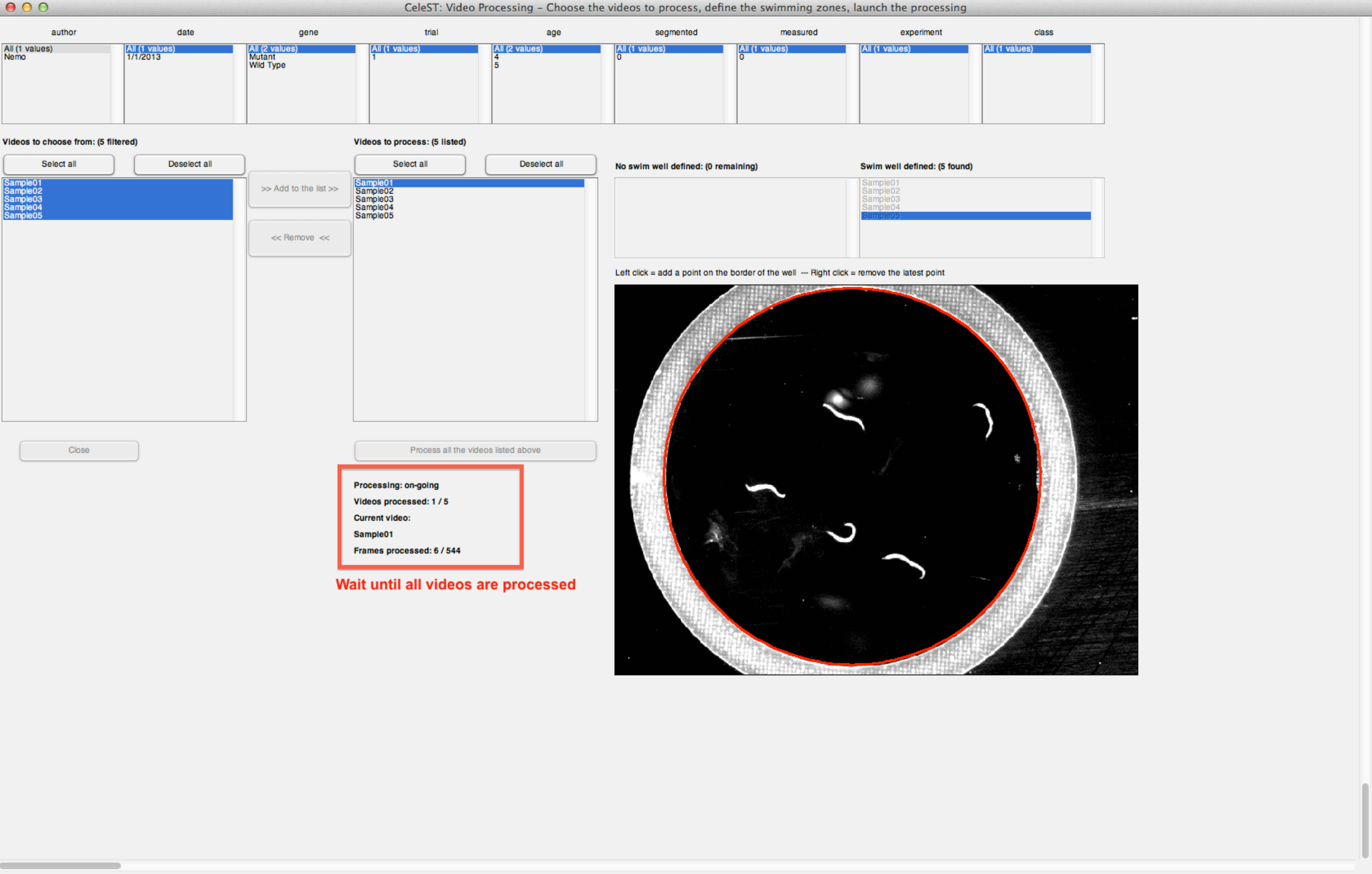
CeleST: Video Processing – Choose the videos to process, define the swimming zones, launch the processing
author
date
gene
trial
age
segmented
measured
experiment
class
All (1 values)
Nemo
All (1 values)
1/1/2013
All (2 values)
Mutant
Wild Type
All (1 values)
1
All (2 values)
4
5
All (1 values)
0
All (1 values)
0
All (1 values)
All (1 values)
Videos to choose from: (5 filtered)
Select all
Deselect all
Sample01
Sample02
Sample03
Sample04
Sample05
>> Add to the list >>
<< Remove <<
Videos to process: (5 listed)
Select all
Deselect all
Sample01
Sample02
Sample03
Sample04
Sample05
No swim well defined: (0 remaining)
Swim well defined: (5 found)
Sample01
Sample02
Sample03
Sample04
Sample05
Left click = add a point on the border of the well --- Right click = remove the latest point
Close
Process all the videos listed above
Processing: on-going
Videos processed: 1 / 5
Current video:
Sample01
Frames processed: 6 / 544
Wait until all videos are processed

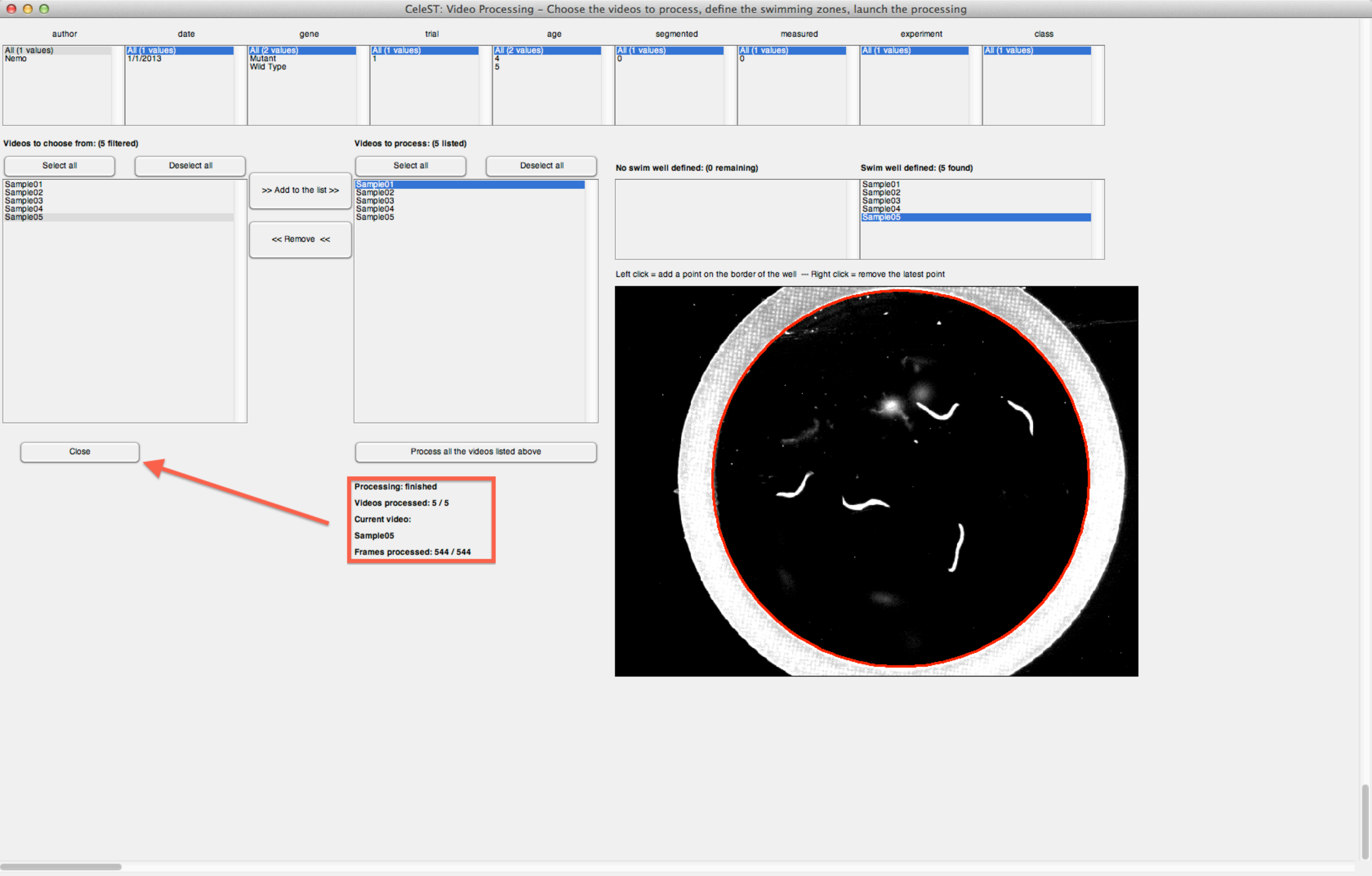
CeleST: Video Processing – Choose the videos to process, define the swimming zones, launch the processing
author
date
gene
trial
age
segmented
measured
experiment
class
All (1 values)
Nemo
All (1 values)
1/1/2013
All (2 values)
Mutant
Wild Type
All (1 values)
1
All (2 values)
4
5
All (1 values)
0
All (1 values)
0
All (1 values)
All (1 values)
Videos to choose from: (5 filtered)
Select all
Deselect all
Sample01
Sample02
Sample03
Sample04
Sample05
>> Add to the list >>
<< Remove <<
Videos to process: (5 listed)
Select all
Deselect all
Sample01
Sample02
Sample03
Sample04
Sample05
No swim well defined: (0 remaining)
Swim well defined: (5 found)
Sample01
Sample02
Sample03
Sample04
Sample05
Left click = add a point on the border of the well --- Right click = remove the latest point
Close
Process all the videos listed above
Processing: finished
Videos processed: 5 / 5
Current video:
Sample05
Frames processed: 544 / 544

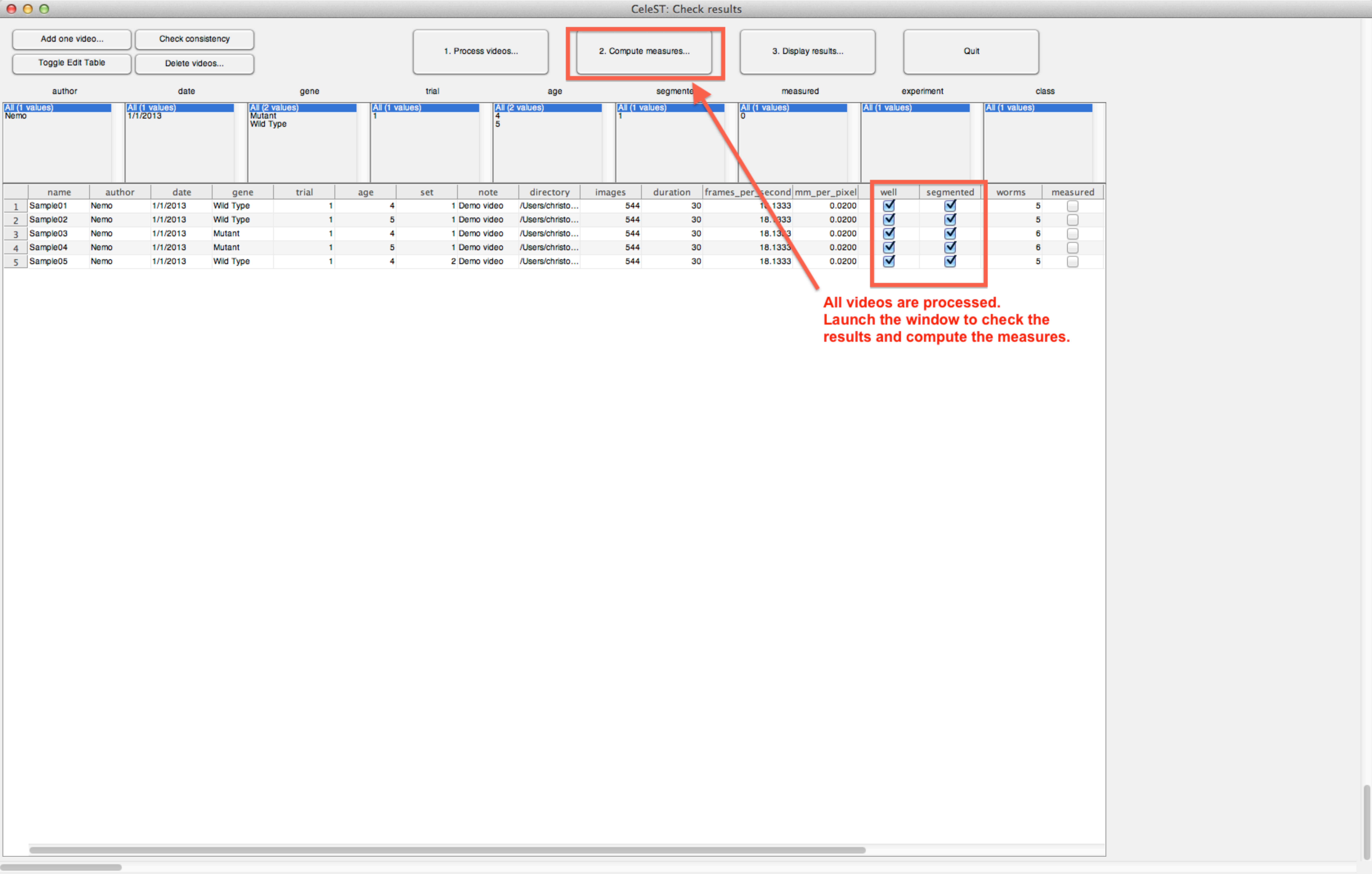

CeleST: Check results
Add one video...
Check consistency
Toggle Edit Table
Delete videos...
1. Process videos...
2. Compute measures...
3. Display results...
Quit
author | date | gene | trial | age | segmented | measured | experiment | class
All (1 values) Nemo
All (1 values) 1/1/2013
All (2 values) Mutant Wild Type
All (1 values) 1
All (2 values) 4 5
All (1 values) 1
All (1 values) 0
All (1 values)
All (1 values)
name | author | date | gene | trial | age | set | note | directory | images | duration | frames_per_second | mm_per_pixel | well | segmented | worms | measured
1 Sample01 Nemo 1/1/2013 Wild Type 1 4 1 Demo video /Users/christo... 544 30 .1333 0.0200 5
2 Sample02 Nemo 1/1/2013 Wild Type 1 5 1 Demo video /Users/christo... 544 30 .333 0.0200 5
3 Sample03 Nemo 1/1/2013 Mutant 1 4 1 Demo video /Users/christo... 544 30 0.0200 6
4 Sample04 Nemo 1/1/2013 Mutant 1 5 1 Demo video /Users/christo... 544 30 18.1333 0.0200 6
5 Sample05 Nemo 1/1/2013 Wild Type 1 4 2 Demo video /Users/christo... 544 30 18.1333 0.0200 5
All videos are processed.
Launch the window to check the results and compute the measures.

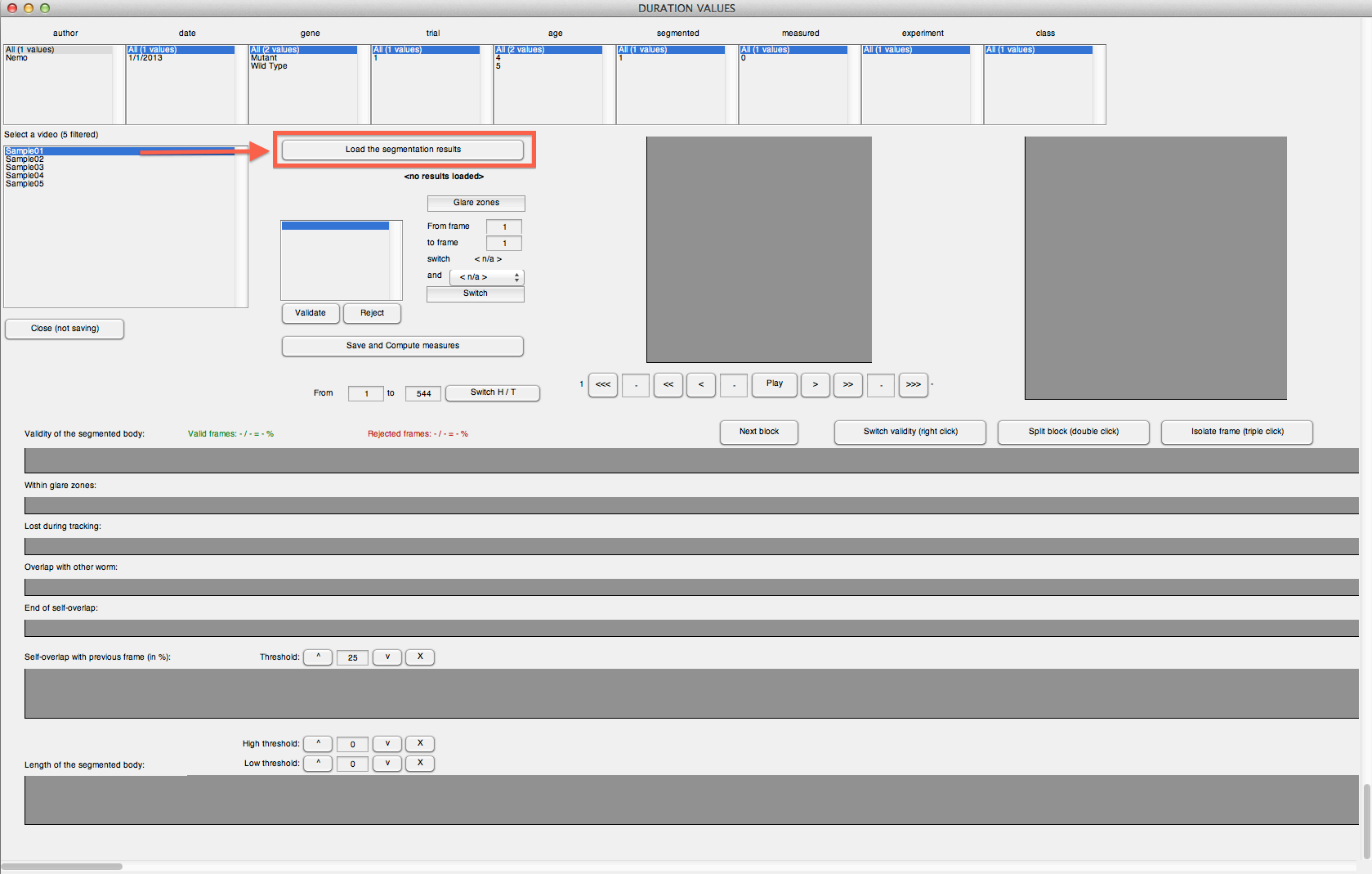
DURATION VALUES
author
date
gene
trial
age
segmented
measured
experiment
class
All (1 values)
Nemo
All (1 values)
1/1/2013
All (2 values)
Mutant
Wild Type
All (1 values)
1
All (2 values)
4
5
All (1 values)
1
All (1 values)
0
All (1 values)
All (1 values)
Select a video (5 filtered)
Sample01
Sample02
Sample03
Sample04
Sample05
Load the segmentation results
<no results loaded>
Glare zones
From frame 1
to frame 1
switch < n/a >
and < n/a >
Switch
Validate
Reject
Close (not saving)
Save and Compute measures
From 1 to 544
Switch H / T
1
<<<
<<
<
Play
>
>>
>>>
Validity of the segmented body:
Valid frames: - / - = - %
Rejected frames: - / - = - %
Next block
Switch validity (right click)
Split block (double click)
Isolate frame (triple click)
Within glare zones:
Lost during tracking:
Overlap with other worm:
End of self-overlap:
Self-overlap with previous frame (in %):
Threshold: ^ 25 v X
High threshold: ^ 0 v X
Low threshold: ^ 0 v X
Length of the segmented body:

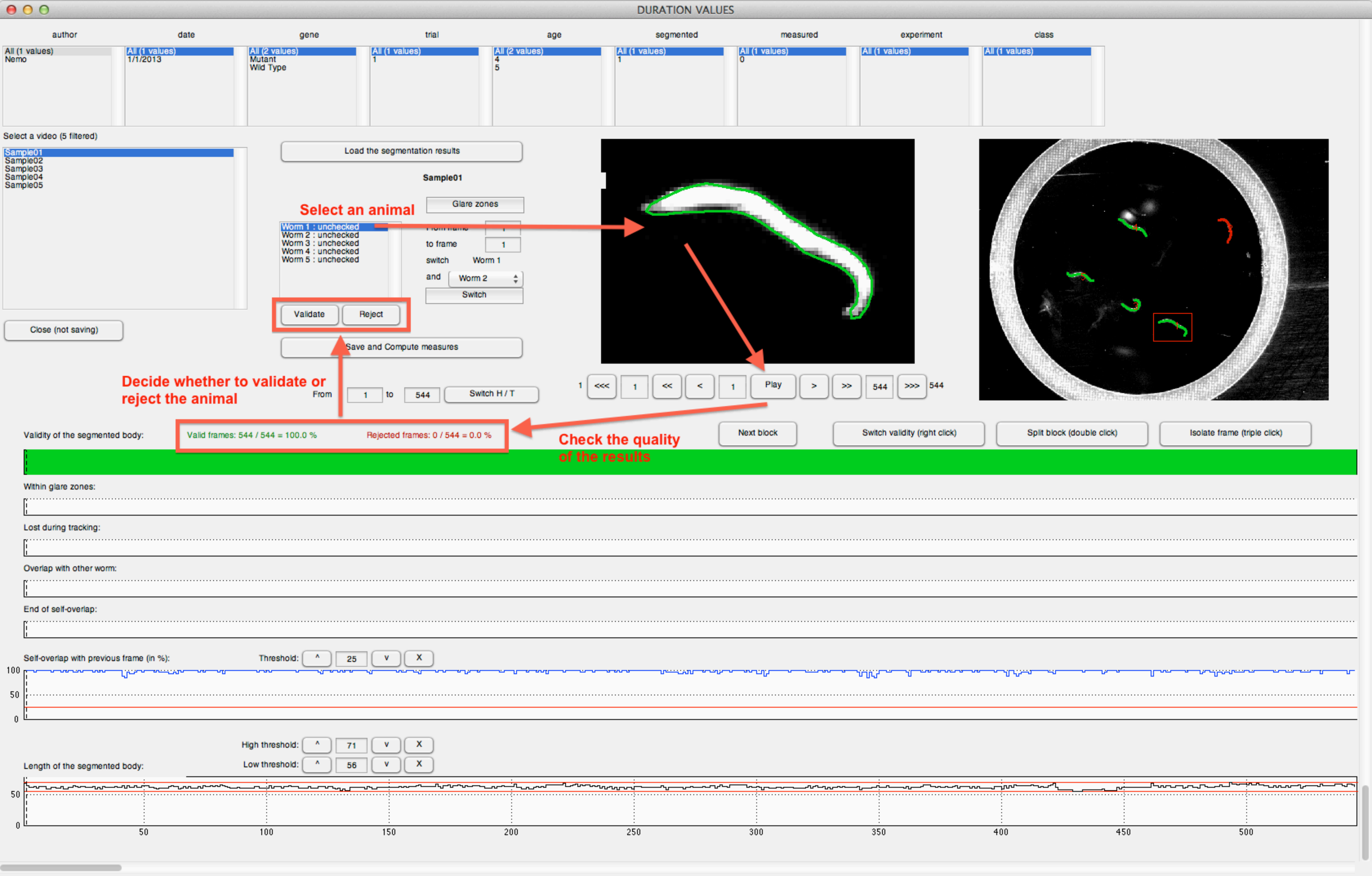
DURATION VALUES
author
date
gene
trial
age
segmented
measured
experiment
class
All (1 values)
Nemo
1/1/2013
All (2 values)
Mutant
Wild Type
Select a video (5 filtered)
Sample01
Sample02
Sample03
Sample04
Sample05
Load the segmentation results
Sample01
Glare zones
Select an animal
Worm 1 : unchecked
Worm 2 : unchecked
Worm 3 : unchecked
Worm 4 : unchecked
Worm 5 : unchecked
to frame
switch
Worm 1
and
Worm 2
Switch
Validate
Reject
Close (not saving)
Save and Compute measures
Decide whether to validate or reject the animal
From
to
Switch H / T
Play
Validity of the segmented body:
Valid frames: 544 / 544 = 100.0 %
Rejected frames: 0 / 544 = 0.0 %
Check the quality of the results
Next block
Switch validity (right click)
Split block (double click)
Isolate frame (triple click)
Within glare zones:
Lost during tracking:
Overlap with other worm:
End of self-overlap:
Self-overlap with previous frame (in %):
Threshold:
High threshold:
Low threshold:
Length of the segmented body:

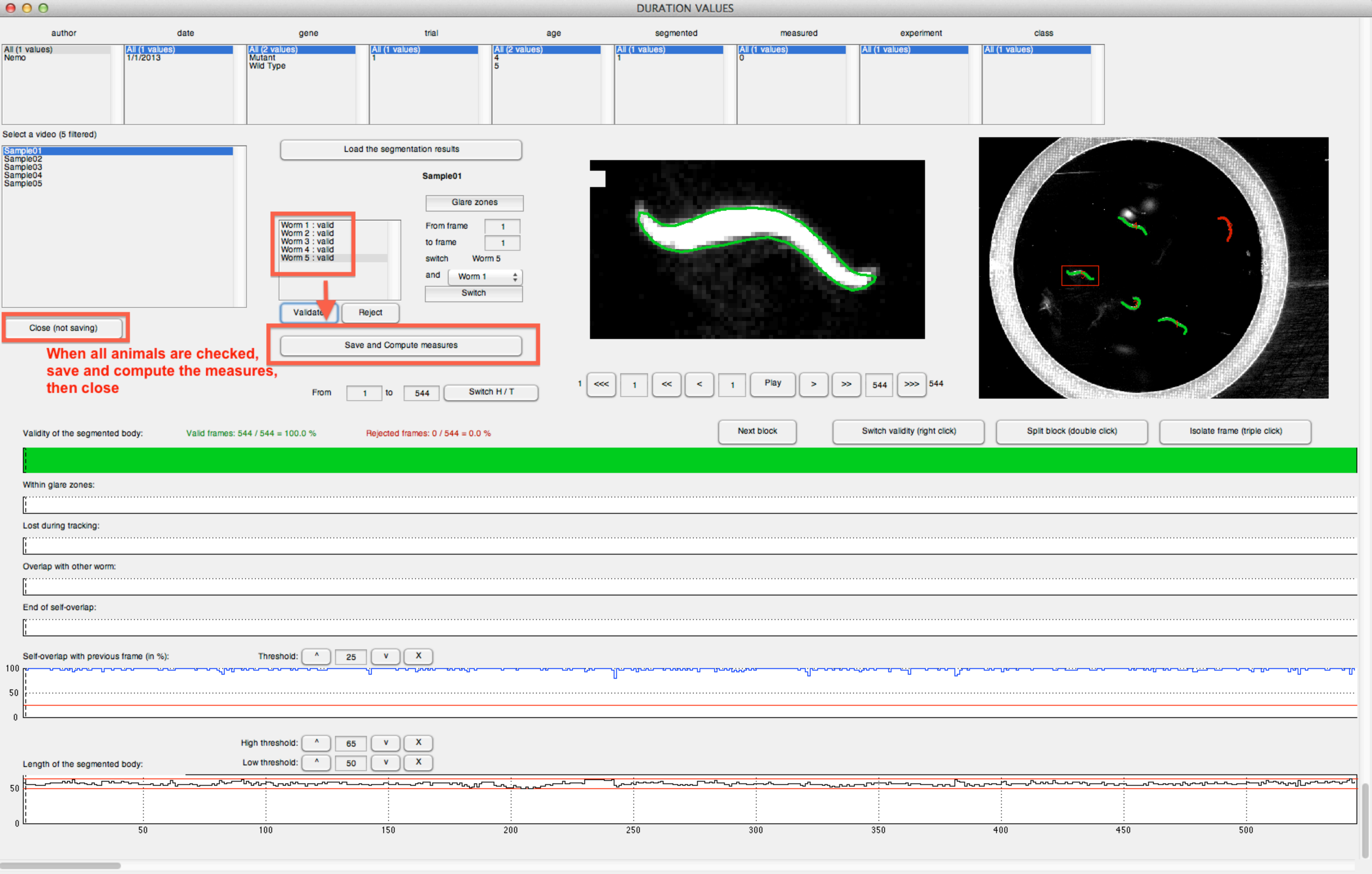
DURATION VALUES
author
date
gene
trial
age
segmented
measured
experiment
class
All (1 values)
Nemo
1/1/2013
All (2 values)
Mutant
Wild Type
Select a video (5 filtered)
Sample01
Sample02
Sample03
Sample04
Sample05
Load the segmentation results
Sample01
Glare zones
From frame
to frame
switch
Worm 5
and
Worm 1
Switch
Worm 1 : valid
Worm 2 : valid
Worm 3 : valid
Worm 4 : valid
Worm 5 : valid
Validate
Reject
Close (not saving)
Save and Compute measures
When all animals are checked, save and compute the measures, then close
From
to
Switch H / T
Play
Validity of the segmented body:
Valid frames: 544 / 544 = 100.0 %
Rejected frames: 0 / 544 = 0.0 %
Next block
Switch validity (right click)
Split block (double click)
Isolate frame (triple click)
Within glare zones:
Lost during tracking:
Overlap with other worm:
End of self-overlap:
Self-overlap with previous frame (in %):
Threshold:
High threshold:
Low threshold:
Length of the segmented body:

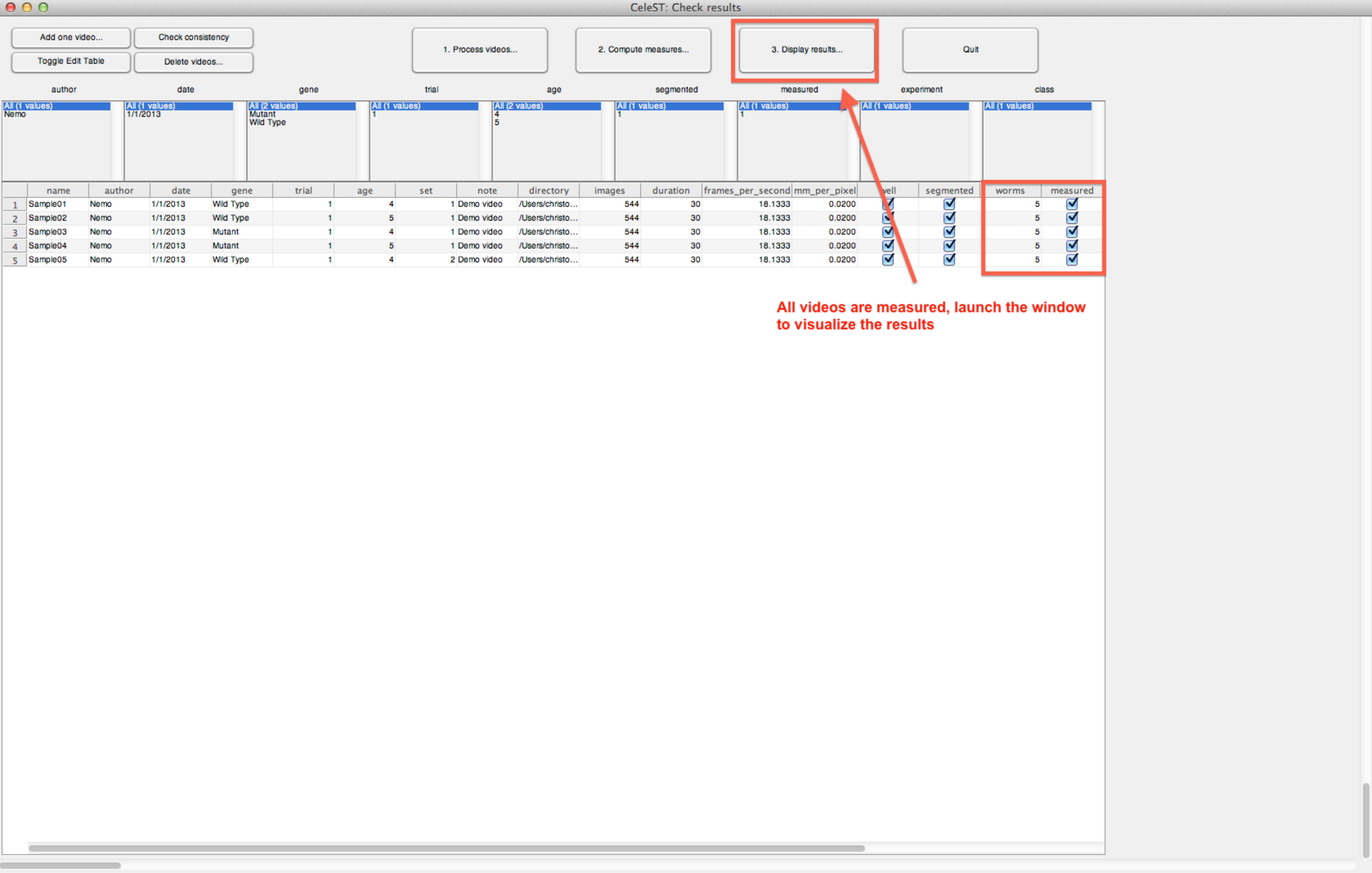
CeleST: Check results
Add one video...
Check consistency
Toggle Edit Table
Delete videos...
1. Process videos...
2. Compute measures...
3. Display results...
Quit
author
date
gene
trial
age
segmented
measured
experiment
class
All (1 values)
Nemo
All (1 values)
1/1/2013
All (2 values)
Mutant
Wild Type
All (1 values)
1
All (2 values)
4
5
All (1 values)
1
All (1 values)
1
All (1 values)
All (1 values)
name author date gene trial age set note directory images duration frames_per_second mm_per_pixel well segmented worms measured
1 Sample01 Nemo 1/1/2013 Wild Type 1 4 1 Demo video /Users/christo... 544 30 18.1333 0.0200 5
2 Sample02 Nemo 1/1/2013 Wild Type 1 5 1 Demo video /Users/christo... 544 30 18.1333 0.0200 5
3 Sample03 Nemo 1/1/2013 Mutant 1 4 1 Demo video /Users/christo... 544 30 18.1333 0.0200 5
4 Sample04 Nemo 1/1/2013 Mutant 1 5 1 Demo video /Users/christo... 544 30 18.1333 0.0200 5
5 Sample05 Nemo 1/1/2013 Wild Type 1 4 2 Demo video /Users/christo... 544 30 18.1333 0.0200 5
All videos are measured, launch the window to visualize the results

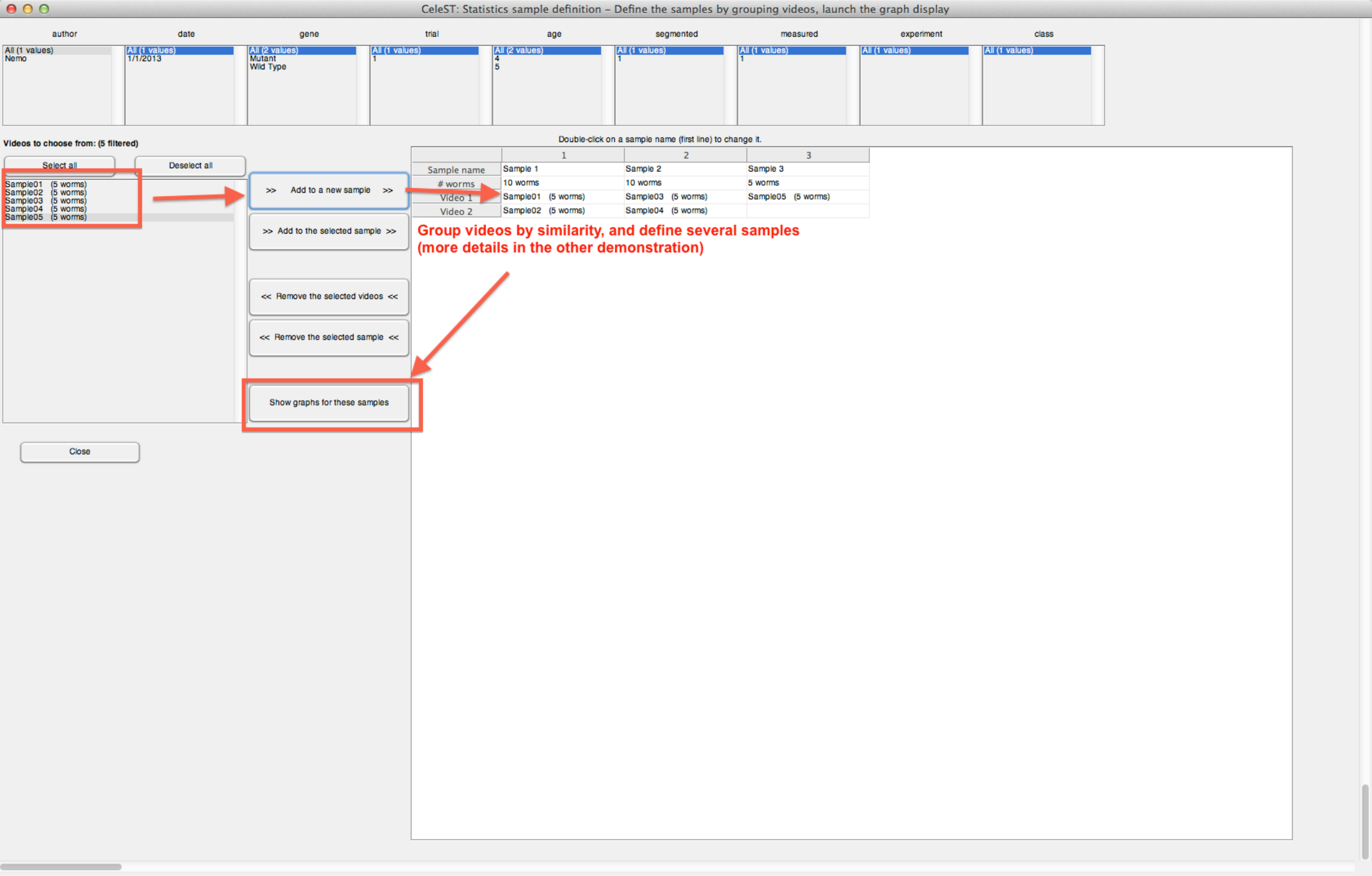
CeleST: Statistics sample definition – Define the samples by grouping videos, launch the graph display
author
date
gene
trial
age
segmented
measured
experiment
class
All (1 values)
Nemo
All (1 values)
1/1/2013
All (2 values)
Mutant
Wild Type
All (1 values)
1
All (2 values)
4
5
All (1 values)
1
All (1 values)
1
All (1 values)
All (1 values)
Videos to choose from: (5 filtered)
Select all
Deselect all
Sample01 (5 worms)
Sample02 (5 worms)
Sample03 (5 worms)
Sample04 (5 worms)
Sample05 (5 worms)
>> Add to a new sample >>
>> Add to the selected sample >>
<< Remove the selected videos <<
<< Remove the selected sample <<
Show graphs for these samples
Close
Double-click on a sample name (first line) to change it.
1
2
3
Sample name
Sample 1
Sample 2
Sample 3
worms
10 worms
10 worms
5 worms
Video 1
Sample01 (5 worms)
Sample03 (5 worms)
Sample05 (5 worms)
Video 2
Sample02 (5 worms)
Sample04 (5 worms)
Group videos by similarity, and define several samples
(more details in the other demonstration)

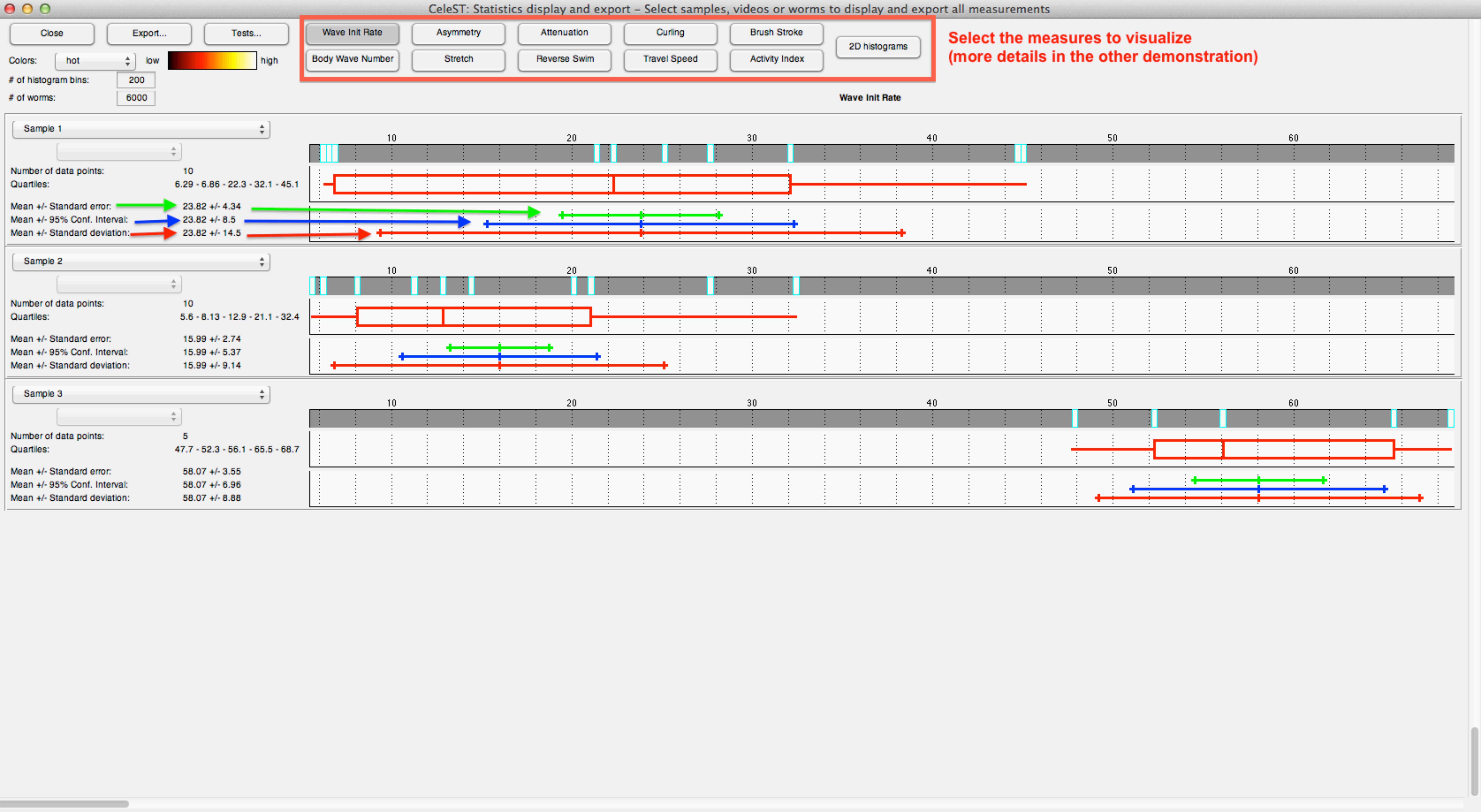
CeleST: Statistics display and export – Select samples, videos or worms to display and export all measurements
Close
Export...
Tests...
Wave Init Rate
Asymmetry
Attenuation
Curling
Brush Stroke
2D histograms
Body Wave Number
Stretch
Reverse Swim
Travel Speed
Activity Index
Select the measures to visualize
(more details in the other demonstration)
Colors:
hot
low
high
# of histogram bins:
200
# of worms:
6000
Wave Init Rate
Sample 1
Number of data points: 10
Quartiles: 6.29 - 6.86 - 22.3 - 32.1 - 45.1
Mean +/- Standard error: 23.82 +/- 4.34
Mean +/- 95% Conf. Interval: 23.82 +/- 8.5
Mean +/- Standard deviation: 23.82 +/- 14.5
Sample 2
Number of data points: 10
Quartiles: 5.6 - 8.13 - 12.9 - 21.1 - 32.4
Mean +/- Standard error: 15.99 +/- 2.74
Mean +/- 95% Conf. Interval: 15.99 +/- 5.37
Mean +/- Standard deviation: 15.99 +/- 9.14
Sample 3
Number of data points: 5
Quartiles: 47.7 - 52.3 - 56.1 - 65.5 - 68.7
Mean +/- Standard error: 58.07 +/- 3.55
Mean +/- 95% Conf. Interval: 58.07 +/- 6.96
Mean +/- Standard deviation: 58.07 +/- 8.88
10
20
30
40
50
60
